# Fly palaeo-evo-devo: immature stages of bibionomorphan dipterans in Baltic and Bitterfeld amber

**DOI:** 10.7717/peerj.7843

**Published:** 2019-10-10

**Authors:** Viktor A. Baranov, Mario Schädel, Joachim T. Haug

**Affiliations:** 1 Biology II, Ludwig-Maximilians-Universität München, Planegg, Bayern, Germany; 2 Geobio-Center, Ludwig-Maximilians-Universität München, München, Bayern, Germany

**Keywords:** Diptera, Larvae, Pupae, Development, Amber, Anisopodidae, Pachyneuridae, Bibionidae, Eocene

## Abstract

Larvae of flies and gnats (Diptera) form a crucial component of many terrestrial and freshwater ecosystems in the extant biosphere. Larvae of Diptera play a central role in water purification, matter and energy transfer in riparian ecosystems in rivers, carbon cycling in lakes and forests as well as being major decomposers of dead organic matter. Despite all these important roles, dipteran larvae are most often ignored in palaeoecological studies, due to the difficulty of the taxonomic identification of fossil larvae, but also due to the perceived importance of adult dipterans in palaeoentomological and taxonomic studies. Despite that, much information on palaeoecosystems can be gained from studying fossil dipteran larvae, in particular for well preserved specimens from fossil resins (ambers and copals). Since ambers are selectively preserving fauna of trunks and leaf litter, it allows us to learn a lot about xylophages and saprophages of amber forests, such as Eocene Baltic amber forest. Here we present immature stages (larvae and pupae) of the dipteran ingroup Bibionomorpha, from Baltic and Bitterfeld amber forests. We have recorded at least four different larval morphotypes, one with four distinct instars, and at least three pupal morphotypes. One larva is recognised as a new species and can be interpreted either as a representative of a highly derived ingroup of Bibionidae or as a sister species to Bibionidae. Also represented by single larval specimens are the groups *Pachyneura* (Pachyneuridae) and *Sylvicola* (Anisopodidae). The majority of the recorded specimens are representatives of the group *Mycetobia* (Anisopodidae). Due to the abundance of immature stages of *Mycetobia*, we have been able to reconstruct the number of larval stages (4) and relative growth rate of these fossil dipterans. We discuss implications of these finds.

## Introduction

Holometabola is a hyperdiverse group of organisms, representing the dominant part of animal life in terrestrial ecosystems (Grimaldi & Engel, 2005). Representatives of the group such as bees, butterflies, beetles and mosquitoes are therefore the best known forms of Insecta to most people. The dominance of holometabolans has led researchers to consider Holometabola as one of the largest groups of Metazoa (Grimaldi & Engel, 2005; Engel, 2019). The evolution of niche differentiation between the larva and the adult (see Haug, in press) has been interpreted as one of the driving factors of their success. The evolutionary independence of different life stages and phases (see Scholtz, 2005) has allowed holometabolans to utilize a very wide spectrum of habitats and ecological niches (Grimaldi & Engel, 2005).

Larvae of flies and midges (representatives of the group Diptera) are successful in diverse habitats, from glaciers at the Antarctic mainland to the fast-drying rock pools of central Africa (Armitage, Pinder & Cranston, 2012; Marshall, 2012). Due to such variety of habitats occupied, larvae of Diptera have become involved in numerous critical ecosystem functions (Marshall, 2012). Dipteran larvae are crucial saprophages, recycling dead organic matter in both aquatic and terrestrial ecosystems, and therefore heavily influence biogeochemical cycles of matter and energy, for example in riparian ecosystems (Marshall, 2012; McAlister, 2017). This ecological role of larval forms of Diptera became especially important about 80 million years ago, in the Upper Cretaceous, when due to the Cretaceous Terrestrial Revolution (CTR) angiosperm plants have become the dominant players in the ecosystem (Fastovsky et al., 2004; McKenna et al., 2015).

The emergence of angiosperm plants in terrestrial ecosystem probably led to an increased load of dead organic matter into terrestrial and freshwater ecosystems (Kalugina, 1974a; Kalugina, 1974b; McKenna et al., 2015). Such a drastic ecosystem change has led to shifts in the communities of various lineages of Insecta (Kalugina, 1974a; Kalugina, 1974b). Such shifts included the extinction or decline of certain systematic and ecological groups. Among them were nectic and benthic oxyphilic forms living in dystrophic lakes. Vice versa, other groups, such as specialized pollinators or saprophages, have experienced an enormous diversification (Sinichenkova & Zherikhin, 1996). Among the groups experiencing a pronounced diversification were many ingroups of Diptera (Grimaldi & Engel, 2005). Numerous groups of dipterans with terrestrial larvae are associated with decaying organic material, such as dead wood, fungal fruit bodies, dead leaves, or animals corpses (Keilin & Tate, 1940; Marshall, 2012). Among the most abundant extant saprophagous forms of Diptera (with predominantly terrestrial larvae) are representatives of Bibionomorpha (Marshall, 2012; Ševčík et al., 2016).

**Figure 1:**
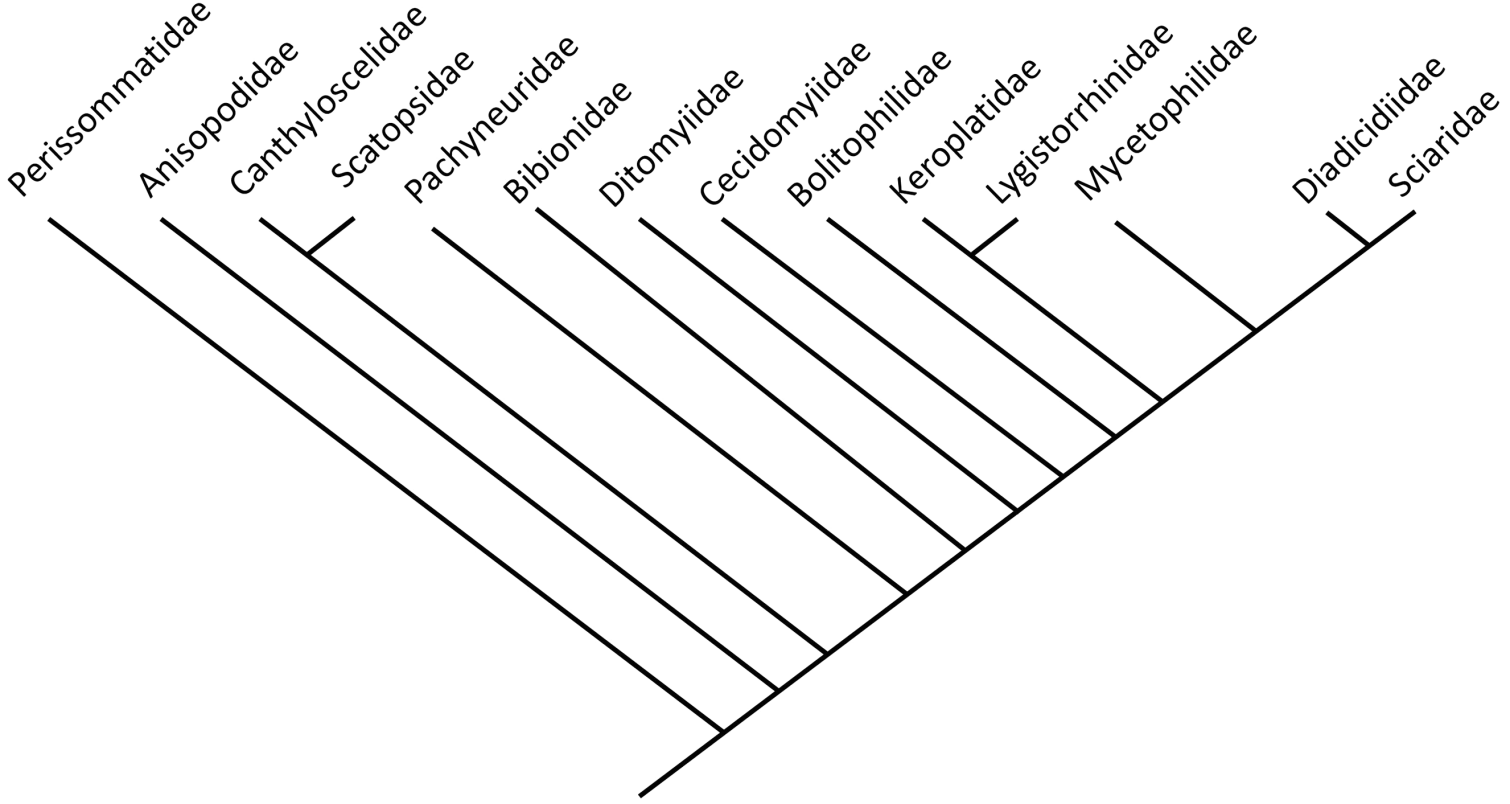
Phylogenetic relationship among different lineages of Bibionomorpha sensu lato, modified from Ševčík et al., 2016. Bayesian hypothesis for relationships among selected taxa of Bibionomorpha based on DNA sequence data (18S, 28S, CAD, 12S, 16S, and COI), 5,018 characters. Position of the Perissommatidae were inferred from comparing phylogeny from (Marshall, 2012 with Ševčík et al., 2016).

Bibionomorpha includes numerous ingroups diverse representatives. However, larvae of Bibionomorpha are predominantly restricted to terrestrial habitats (Fig. 1, modified and simplified from Ševčík et al., 2016).

**Table 1:** List of material examined.

ID-Number	Taxa	Specimens	Syninclusions	Deposited	Origin
GPIH-Schlee-0024	Dinobibio hoffeinseorum	1	Acalyptrata	GPIH	Baltic
Dip-00642	larvae Sylvicola (?)	1	Plant material+ stellate hairs	DEI	Baltic
PED-4395	Mycetobia connexa	male, female, pupal exuvia	partial inclusion of an adult beetle	PED	Baltic
BI-2350	Mycetobia larvae	1	none	GPIH	Bitterfeld
GPIH-3706 W	Mycetobia larvae	1	Phoridae adult+stellate hairs	GPIH	Baltic
Dip-00639	Mycetobia larvae	1		DEI	Baltic
PED-4965	Mycetobia larvae	1		PED	Baltic
PED-4970	Mycetobia larvae	1		PED	Baltic
PED-5695	Mycetobia larvae	1	Cicadellidae nymph, larva of Coccidoidea, worker ant and non-biting midge female (Diptera: Chironomidae: Tanytarsini)	PED	Baltic
GPIH-L-7592	Mycetobia larvae	2	Fragment of the Diptera Brachycera female, mites, stellate hairs	GPIH	Baltic
Dip-00640	Mycetobia larvae	3	2 males, 1 female Rheosmittia pertenuis	DEI	Baltic
PED-4748	Mycetobia larvae	4		PED	Baltic
GPIH-Schlee-0247	Mycetobia larvae	9	“Lepidoptera” (Trichoptera), + fragment of a beetle	GPIH	Baltic
AKBS-00071	Mycetobia pupa mt 1	1	Lasius schiefferdeckeri+Ctenobethylus geopperti	GPIH	Baltic
GPIH-1851DN	Mycetobia pupa mt 1	1	2 Keroplatidae males, Sciaridae male+ probably male of Anisopodidae	GPIH	Baltic
Dip-00641	Mycetobia pupa mt 1	1	Plant material, insect tarsi fragment	DEI	Baltic
GPIH-N-7095	Mycetobia pupa mt 1	1	Neurothidae larvae, ants 2, Dolichopodidae flies x2, Trichoptera adult, insects i.s. x2	GPIH	Baltic
PED-4998	Mycetobia pupa mt 1	1	spider webs	PED	Baltic
GPIH-L-7514	Mycetobia pupa mt 2	1	Plant material +stellate hair	GPIH	Baltic
PED-4866	Mycetobia pupa mt 2	1	dult rove beetle (Coleoptera: Staphylinidae) and two adult gall midges (Diptera; Cecidomyiidae)	PED	Baltic
GPIH -7516	Pachyneuridae larvae	1	stellate hairs	GPIH	Baltic
Dip-00649	Mycetobia larvae	5	Orthocladiinae female	DEI	Baltic
Dip-00650	Mycetobia pupa	1		DEI	Bitterfeld
Dip-00651	Mycetobia pupa	1		DEI	Baltic
Dip-00652	Mycetobia pharrate adult	1		DEI	Baltic
Dip-00653	Mycetobia pupa	1		DEI	Baltic
Dip-00654	Nematocera larvae sp	3		DEI	Baltic
Dip-00655	Mycetobia pup 2, 2 larvae	4	Adult Sciaroidea, adult Limoniidae	DEI	Baltic
Dip-00656	Mycetobia larvae	3	Ants, Cecidomyiidae	DEI	Baltic
Dip-00657	Mycetobia pupa mt1	1		DEI	Baltic
Dip-00658	Mycetobia larvae	1		DEI	Baltic
Dip-00659	Mycetobia pupa mt1	1		DEI	Baltic
Dip-00660	Mycetobia pharrate adult	1		DEI	Baltic
Dip-00661	Mycetobia pupa mt1	1		DEI	Bitterfeld
Dip-00662	Mycetobia pupa mt2	2		ZSM	extant
Dip-00663	Mycetobia pupa mt3	3		ZSM	extant
Dip-00664	Mycetobia pupa mt4	4		CeNak	extant
MB.I.7295	Mycetobia pupa mt1	1		MfNB	Baltic
NA	Mycetobia pallipes Meigen, 1818	>50		ZSM	Ober-Bayern, Fürstenfeldbruck, Roßkastanie, Wundausfluß, Bayern, Germany, 29.5-4.7.1994, leg. W. Schlacht.
NA	Penthetria funebris Meigen, 1804.	>50		ZSM	Augsburg, Lechau nördl. St. Stephan, Barb-F., Auwald- Ruderal, 440 m, 27.05.1981, Schmidt.
NA	*Bibio varipies* Meigen, 1830	1		CeNak	NA

The geological history of Bibionomorpha spans more than 220 million years (Blagoderov, Grimaldi & Fraser, 2007). Many representatives are known from the late Triassic (Blagoderov, Grimaldi & Fraser, 2007) and Jurassic (Kalugina & Kovalev, 1985). Despite the long evolutionary history of the group and the ecological importance of their larval stages, very little attention has been paid to the fossil record of immature stages of Bibionomorpha (Harris, 1983; Skartveit, 2009). This is surprising, as immature representatives of Bibionomorpha, especially those of Anisopodidae, seem to be quite common in amber, as we will demonstrate. Despite such abundance, Anisopodidae larvae from amber were only mentioned in a single study focused on specimens from Dominican amber (Grimaldi, 1991).

Here, we present a first overview of the immature stages of Bibionomorpha from amber, including larvae and pupae of Anisopodidae, larvae of Pachyneuridae and a species that seems closely related to Bibionidae. All specimens in focus of this study are preserved in Eocene Bitterfeld amber and Baltic ambers (Table 1). We also discuss the implications of the morphological and ecological diversity of immature representatives of Bibionomorpha in relation to the ecology and biogeochemistry of the Eocene amber forests.

## Materials & Methods

### Material

All specimens in the center of this study, in total 56, are preserved in amber and come from various collections. A full list of the examined material is given in Table 1.

Part of the material (see Table 1, material marked as “Material from Hoffeins collection”) was obtained commercially in 2005 and stems from Yantarnyj, Kaliningrad district (formerly Palmnicken, Königsberg); specimens have temporarily been part of the collection of Christel and Hans-Werner Hoffeins (CCHH). All specimens from this source are now deposited at the Senckenberg Deutsches Entomologisches Institut (SDEI; with inventory numbers listed in Table 1).

Another part of the material comes from the private collection of Carsten Gröhn and is now deposited in the collection of the Center for Natural History in Hamburg (Centrum für Naturkunde, CeNak, formerly Geological-Paleontological Institute and Museum of the University of Hamburg, Geologisch-Paläontologisches Institut und Museum der Universität Hamburg, GPIH).

Part of the material has been commercially obtained from Jonas Damzen (“amberinclusions.eu”) by one of the authors (Joachim T. Haug). This material is now permanently housed in the research collection of the Palaeo-Evo-Devo Research Group, Ludwig-Maximilians-Universität, Munich, Germany (PED). One specimen is part of the collection of the Museum für Naturkunde Berlin (MfNB).

Further material was retrieved from the collection of the Center for Natural History in Hamburg (CeNak).

Information on syninclusions is provided in Table 1 as well. All abbreviations of the collection names are according to the “The insect and spider collections of the world” website (Evenhuis, 2019).

For comparative purposes, we used extant larval representatives of Anisopodidae and Bibionidae (larvae, pupae, and adult) from the collection of the Zoological State Collection, Munich (Zoologische Staatssammlung München, ZSM), in particular, *Sylvicola fenestralis* (Scopoli, 1763) (adult and pupa, no collection number available), *Mycetobia pallipes* Meigen, 1818 (larvae, pupae and adult, no collection number available) and *Penthetria funebris* Meigen, 1804 (larvae, pupae and adult, no collection number available) as well as *Bibio varipies* Meigen 1830, (Centrum für Naturkunde Hamburg—CeNak, no collection number assigned).

The morphological terminology largely follows Borkent & Sinclair (2017). Yet, to enhance the understandability for non-experts, we amended some of the special morphological terms with more general terms. As Insecta is an accepted ingroup of Crustacea s.l. “crustacean”-terms given in square brackets were necessary to provide wider frame correspondence.

### Imaging methods

The specimens were imaged using a Keyence VHX-6000 Digital microscope, with ring-light type illumination and/or cross-polarised, co-axial illumination. All photographic images presented in this paper are composite images. Images were assembled using panoramic stitching to overcome the limitation of the field of view at higher magnifications. For each single image a stack of images of shifting focus was recorded to overcome the limitation of the depth of field (Haug, Haug & Ehrlich, 2008; Haug et al., 2011a; Haug et al., 2011b; Haug, Müller & Sombke, 2013a). Fusion into sharp images and panoramic stitching was performed with the software implemented in the digital microscope (e.g., Haug, Müller & Haug, 2018; Haug et al., 2019). We also used the implemented HDR function of the digital microscope; therefore every single frame is a composite from several images taken under different exposure times (cf. Haug et al., 2013b).

In addition to that, extant and fossil material was imaged using a Keyence BZ-9000 fluorescence microscope with either a 2×, 4×, 10×or 20×objective depending on the size of the objects. Observations were conducted at an emitted wavelength of 532 nm since it was the most compatible with the fluorescence capacities of the fossil specimens (Haug et al., 2011a; Haug et al., 2011b). To counteract the limitation in the depth of the focus we recorded stacks of images which than were digitally fused to single in-focus images using CombineZP (GNU). Extant specimens were imaged using a ZEISS Stemi 508 Stereo Microscope (with 8:1 Zoom with double LED spot K and additional ring light) in combination with a DCM 510 ocular camera. Adobe Photoshop Elements 11 was used to stitch different images to single panoramic images. The resulting images were post-processed in Adobe Photoshop Elements 11 to optimize the histogram and sharpness as well as to amend the images with color markings to highlight morphological structures.

Two specimens (Dip-00653, Dip-00660) were scanned using X-ray computer tomograph Zeiss Xradia XCT-200 in the Zoological Institute and Museum of University of Greifswald. Volume rendering images of the scans were created using Drishti (GNU) (e.g., Hörnig et al., 2016).

Micro-CT scanning of one specimen (MB.I.7295) was performed using a Nanotom m Phoenix (GE Sensing & Inspection Technologies GmbH). Scans were reconstructed to tiff stacks with the built-in software. Tiff stacks were further processed with ImageJ and Osirix 5.8.2 (e.g., Haug et al., 2011a; Haug et al., 2011b; Nagler, Hyžný & Haug, 2017).

### Morphometry

Maximum head capsule length (in dorsal view) and width of some larvae were measured, as suggested by Coombs, Cleworth & Davies (1997), from photos, using ImageJ (public domain; Schneider, Rasband & Eliceiri, 2012). Statistical analysis of the data was performed in R (GNU), using the mblm-function of the mblm-package, with a Theil-Sen single median method as a baseline method for applying Sen slopes to the data (Komsta, 2013). Not all specimens of the Mycetobia larvae had well preserved head capsules, therefore measurements of the width and length were performed for 25 specimens.

### Taxonomy

Wherever possible we decided not to use Linnean ranks (“rankless taxonomy”). Ranks represent arbitrary constructs in a way that they do not hold “comparative values” (Mayr, 1942, p. 291, line 3) and, in our view, do not contribute to an easier understanding of phylogenetic relations among species and higher groups. However, the rank of the genus is not as easy to dismiss as the ranks of higher (broader) systematic groups. This is solely due to its function as part of binomial species names. Even though there are ways to avoid this dilemma such as the application of uninomial nomenclature for species (Lanham, 1965) or the use of any higher systematic group (regardless ranked as genus or not) as part of the species name (Haug & Haug, 2016 following Béthoux, 2010), the traditional, rank based, application of binomial names is still required by the International Code of Zoological Nomenclature (ICZN, Chapter 2, Article 5 & App. B, 6.). To be consistent with the “Code” we establish a new generic name, even though there is only one species assigned to this name and thus the sole purpose of this name is to serve as part of the binomial species name. Hence, until a sister taxon (species or group) to the herein described species is found, the generic name is that of a monotypic taxon and thus no diagnosis can be given for it.

For the sake of consistency, reproducibility and to increase the speed of fossil biodiversity discovery, we applied a matrix-based description scheme, proposed by Haug, Briggs & Haug (2012). We think that such form of description, based on the alternating characters states, entered in the excel sheet are useful in providing consistent, streamlined description, albeit with numerous repetitions of the same phrases.

A single new species is described herein. The electronic version of this article will represent a published work according to the International Commission on Zoological Nomenclature (2012) (ICZN), and hence the new names contained in the electronic version are effectively published according to the ICZN from the electronic edition alone. This published work and the nomenclatural acts it contains have been registered in ZooBank, the online registration system of the ICZN. The ZooBank LSIDs (Life Science Identifiers) can be resolved and the associated information viewed through any standard web browser by appending the LSID to the prefix http://zoobank.org/ The LSID for this publication is: urn:lsid:zoobank.org:pub:7E6FFA31-9DA8-44A6-BE7D-55E6AE34B660. The online version of this work is archived and available from the following digital repositories: PeerJ, PubMed Central and CLOCKSS.

## Results

### Taxonomy

**Table:** 

Diptera Linnaeus, 1758
Bibonomorpha *sensu lato* sensu Ševčík et al., 2016
*Dinobibio* gen. nov.
Life Science Identifier: urn:lsid:zoobank.org:act:8C8DCD9A-1A44-473E-9692-54C7AE204B91.

*Etymology*: from Ancient Greek *δ*ε*ιν*ó*ς* (deinos), meaning ‘terrible, potent or fearfully great’, due to the imposing nature of the larva, which bears large protuberances, and Bibio (ingroup of Bibionidae).

*Type species*: *Dinobibio hoffeinseorum* sp. nov. by present designation.

Life Science Identifier: urn:lsid:zoobank.org:act:80D4F834-D0D4-404F-AE02-C8FF184D4943

*Remark:* no diagnosis can be given, since the new generic name does not refer to a natural group but is only put up to provide a binomial name (see explanation above). However, for the purposes of consistency we are providing putative diagnosis, identical, but abbreviated in comparison to the type species. Larva characterized by cylindrical body-shape; maxillary palp with additional strong process distally on the element 1; trunk protuberances expanding towards mid length and then tapering again; terminal abdominal spiracle, situated dorso-laterally, not larger than the rest of the spiracles.

**Table:** 

**Figure 2:**
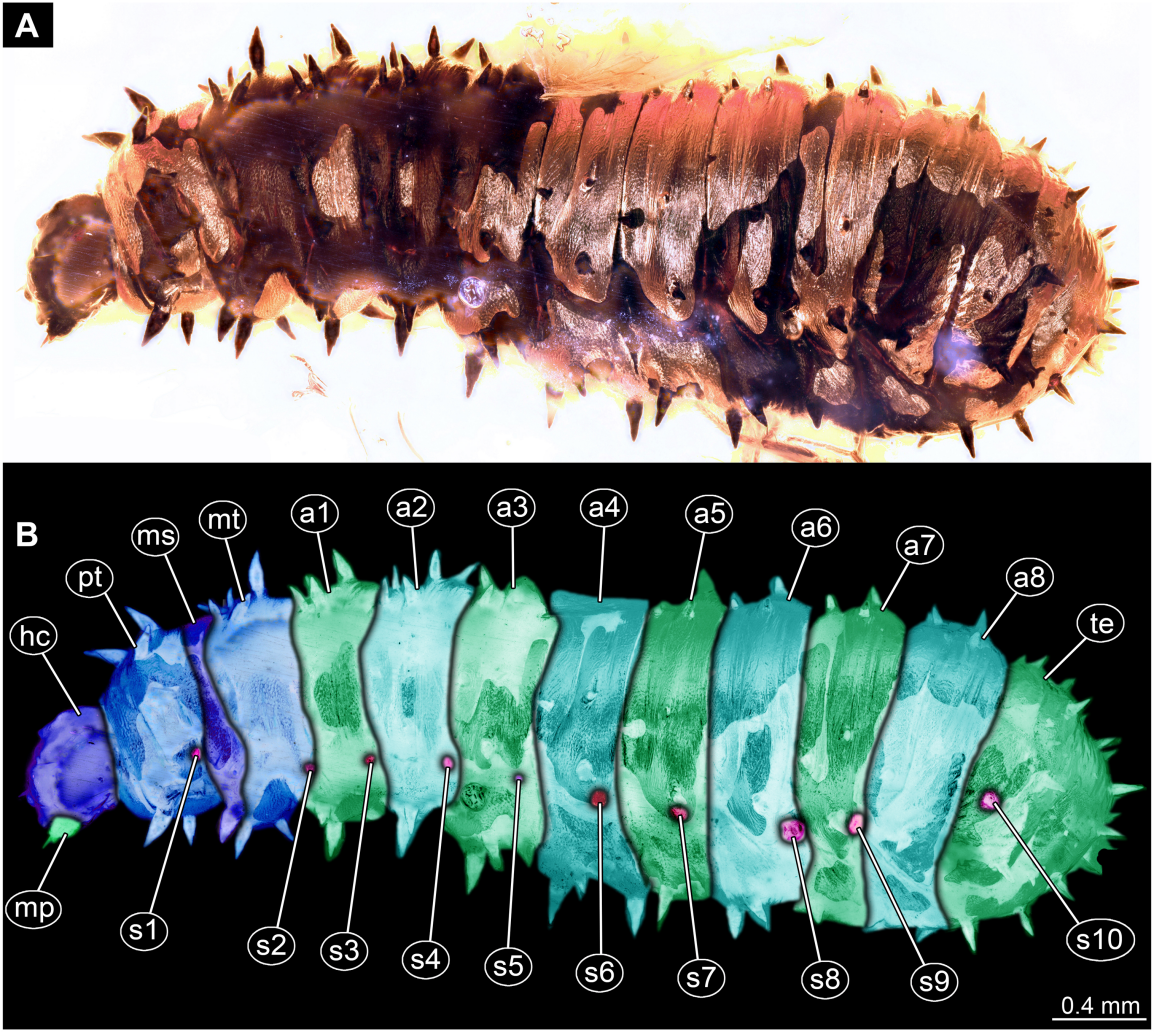
Dipteran larva, holotype of *Dinobibio hoffeinseorum* sp.n. GPIH, accession number (GPIH-0024) in lateral view. (A) Overview, composite image. (B) Colored version of A above. Abbreviations: a1–a8, abdominal segment 1–8; hc, head capsule; mp, maxillary process; ms, mesothorax; mt, metathorax, pt, prothorax; s1-s10, spiracle 1–10; te, trunk end.

**Figure 3:**
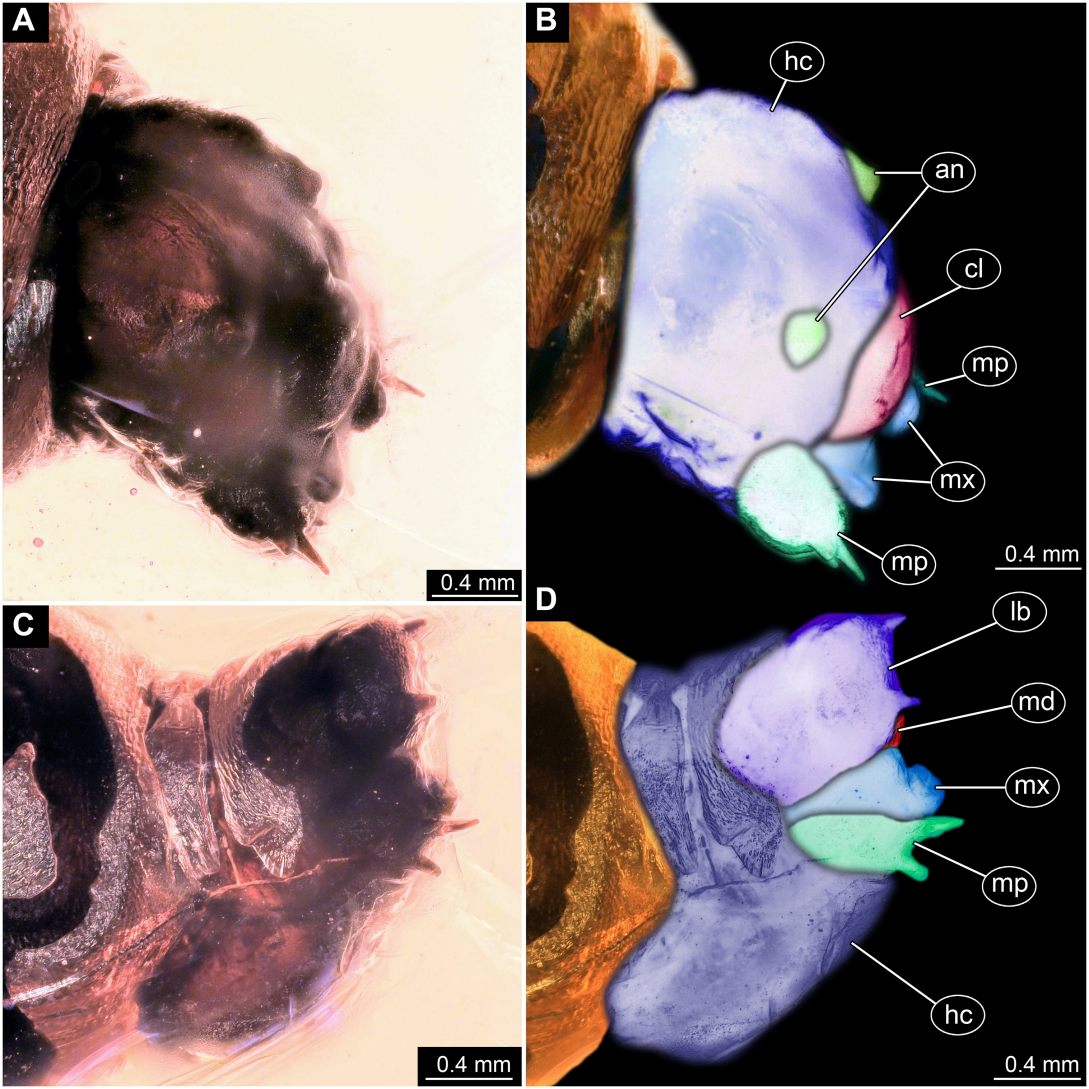
Fossil dipteran larva, holotype of *Dinobibio hoffeinseorum* sp.n. GPIH, accession number (GPIH-0024). (A) Head capsule, latero-dorsal view; (B) Colored version of A. (C) Head capsule, ventrolateral view. (D) Colored version of C. Abbreviations: an, antennae; cl, clypeus; hc, head capsule; lb, labium; md, mandible; mp, maxillary palp; mx, maxilla.

*Dinobibio hoffeinseorum* sp. nov.
(Figs. 2A, 2B; 3A–3D, Fig. S1)

*Holotype*: a single fossil larva, GPIH-0024. The larva is well preserved, but lateral aspects are obscured by a silvery film (probably air bubbles) covering parts of the trunk.

*Etymology*: named after Christel and Hans-Werner Hoffeins for their immense contribution to the general study of dipterans preserved in Baltic amber and Bibionidae in particular.

*Syninclusions:* a single “acalyptrate” fly (“Acalyptrata” = non-monophyletic assemblage of lineages within Brachycera that are not part of Calyptrata). Syninclusion too poorly preserved to identify more precisely.

Description:

**Habitus**. Medium sized larva with a bowling-pin shaped body. Total length 6.4 mm. Body differentiated into presumably 20 segments, ocular segment plus 19 post-ocular segments.

**Head.** Ocular segment and post-ocular segment 1–5 (presumably) forming distinct capsule (head capsule). Head capsule longer than wide. Head capsule in dorsal view not accessible due to orientation of the specimen. Hind part of head capsule partly retracted into anterior trunk. Dimensions of head capsule: 860 µm long, width hard to access. Surface of head capsule with “warty” appearance, bearing numerous bulbous protrusions and smaller spine-like protrusions.

**Ocular segment** without apparent stemmata (larval eyes). Ocular segment recognizable by its appendage derivative, clypeo-labral complex. Clypeus (clypear sclerite) dome-shaped, with several bulbous expansions on the top, total length 260 µm, oval in general shape (Figs. 3A, 3B). Labrum not discernible.

**Post-ocular segment 1** recognizable by its appendages, antennae [antennulae]. Antenna arising from head capsule postero-laterally to the clypeus. Antennae sitting on large piedestal (socket); no subdivision of antenna into elements apparent. (Figs. 3A–3D).

**Post-ocular segment 2** (intercalary segment) without externally recognizable structures (Figs. 3A–3D).

**Post-ocular segment 3** recognizable by its pair of appendages, mandibles. Mandible only accessible at the distal tip, proximal part obscured (Figs. 3A–3D).

**Post-ocular segment 4** recognizable by its appendage, maxilla [maxillula]. Maxilla massive, organised into proximal part and distal part, palp [endopod]. Proximal part differentiated into two lobes, outer lobe and inner lobe. Inner lobe, possible lacinia [endite]. Possible lacinia rectangular in outline. Possible lacinia 100 µm long, 200 µm wide. Palp arising from outer lobe, cylindrical, with two elements [palpomeres]. Element 1 170 µm long. Element 1 distally with strong conical outgrowth. Outgrowth 80 µm long. Element 2 conical, 45 µm long, without apparent armature. (Figs. 3A–3D).

**Post-ocular segment 5** recognizable by its appendages, forming the labium [conjoined left and right maxillae]. Labium massive, heavily sclerotized, with proximal and distal parts, palps [endopods]. Labium occupying over 60% of the total length of the head capsule ventrally. Palp cylindrical, total length 35 µm (Figs. 3C, 3D). Total length of the labium (without palp) 310 µm, width 200 µm.

**Trunk.** Trunk roughly bowling-pin shaped, diameter increasing posteriorly, diameter of the trunk always larger than that of the head capsule (Figs. 2A, 2B). Trunk with 12 visible units, interpreted as 3 thorax segments plus 8 abdominal units and a trunk end representing a conjoined structure of undifferentiated abdominal segments (9–11?). Trunk with abdominal units, progressively increasing in lateral aspect towards the posterior part of the body. Segment 1 1,400 µm high, while 7th 1,790 µm high. Trunk lacks parapodia and/or creeping welts. Trunk bears dozens of conical protuberances on the entire surface. Each segment of the trunk, with the exception of the trunk end, carries 8 prominent, fleshy protuberances dorso-laterally and ventrolaterally in groups of two, four at each side of the body. Protuberances widest at the mid-length, slightly narrower proximally and tapering distally, mean length ca. 270 µm. Trunk surface with numerous small spines (Figs. 2A, 2B; Fig. S1). Trunk bears 10 pairs of spiracles (openings of the tracheal system) (Figs. 2A, 2B). Each spiracle situated in the centre of an elevated ridge (Figs. 2A, 2B).

**Thorax** consists of three segments, pro-, meso- and metathorax.

**Prothorax** sub-equal in width to the head capsule, 670 µm. Prothorax bears a pair of large spiracles. Prothorax carries 8 prominent, fleshy protuberances dorso-laterally and ventrolaterally in groups of two, four at each side of the body.

**Mesothorax** 580 µm long. Mesothorax carries 8 prominent, fleshy protuberances dorso-laterally and ventrolaterally in groups of two, four at each side of the body. Mesothorax with no spiracle openings present.

**Metathorax** 560 µm long. Metathorax carries 8 prominent, fleshy protuberances dorso-laterally and ventrolaterally in groups of two, four at each side of the body. Metathorax bears a pair of spiracles (Figs. 2A, 2B; Fig. S1).

**Abdomen (posterior trunk)** Height of abdominal units progressively increasing in lateral aspect towards the posterior part of the body.

Abdominal units 1–8 each carrying 8 prominent fleshy protuberances dorso-laterally and ventrolaterally in groups of two, four at each side of the body. Abdominal units 1–7 each carrying a pair of spiracles laterally.

Abdominal unit 8 lacks spiracles.

**Trunk end** (undifferentiated abdomen segments 9–11?) shorter than abdominal unit 8. Trunk end bears anus on the posterior part. Trunk end bears more than a dozen of conical protuberances on the entire surface. No protuberances present in the immediate vicinity of the anus, on the postero-dorsal surface of the trunk end. Trunk end bears posterior spiracles with a single ecdysial scar (a site where the previous larval stage cuticle breaks from the spiracle). Posterior spiracle is sub-equal to the rest of the spiracles.

*Differential diagnosis*: The larva is clearly different from any modern representative of Bibionidae, of which immature stages are known based on the combination of the following characters: cylindrical body-shape; a maxillary palp with additional strong process distally on the element 1; trunk protuberances which are expanding towards mid length and then tapering again; terminal abdominal spiracle (abdominal segment 10), situated dorso-laterally, not larger then the rest of the spiracles; (Figs. 2A, 2B; Figs. 3A, 3D).

**Figure 4:**
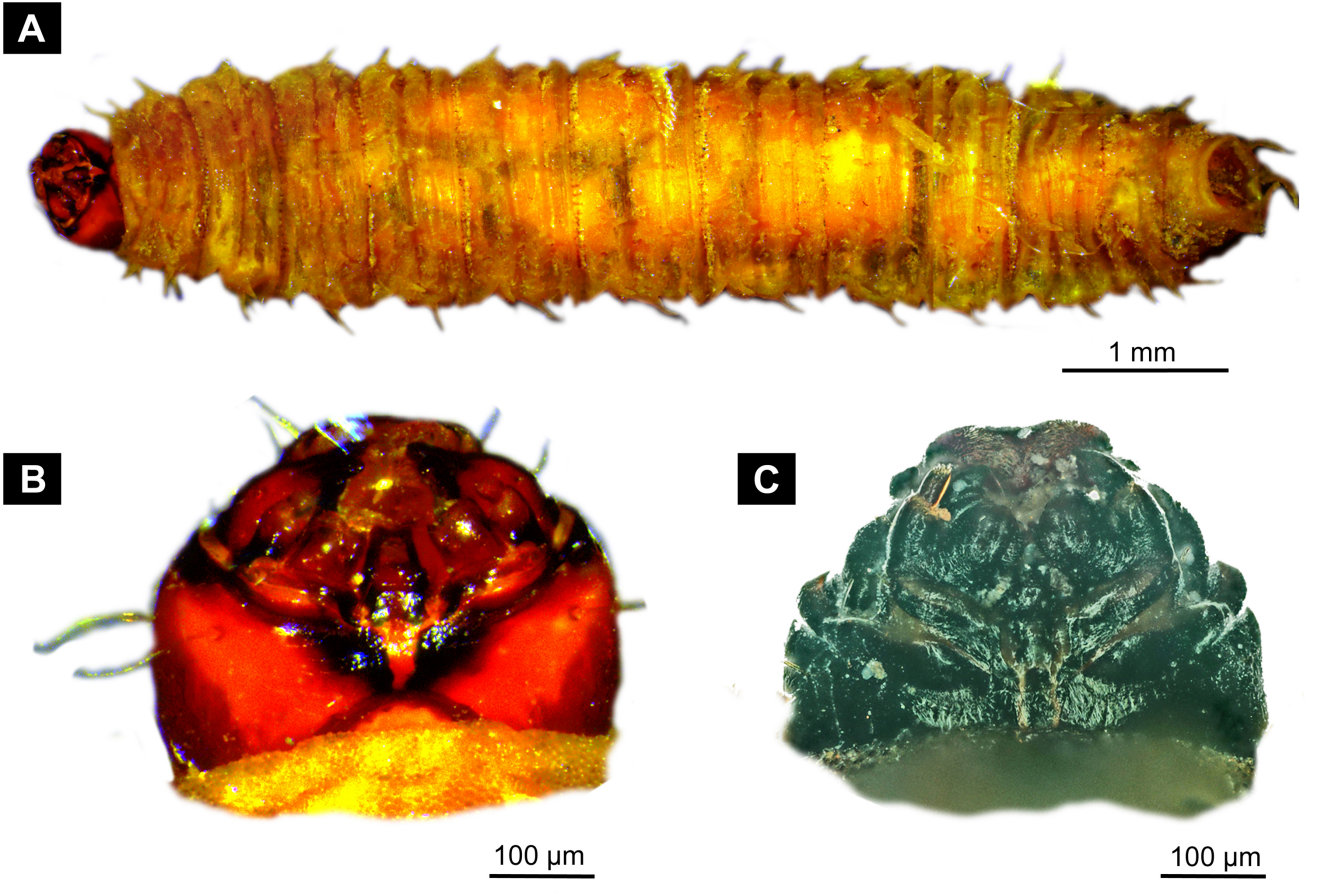
Extant larvae of Bibionidae. (A–B) *Bibio varipies* Meigen, 1830, CeNak, no collection number assigned. (C) *Penthetria funebris* (Meigen, 1804), ZSM, no collection number assigned. (A) habitus ventral. (B) head capsule, ventral. (C) head capsule of fourth instar larva, ventral.

**Figure 5:**
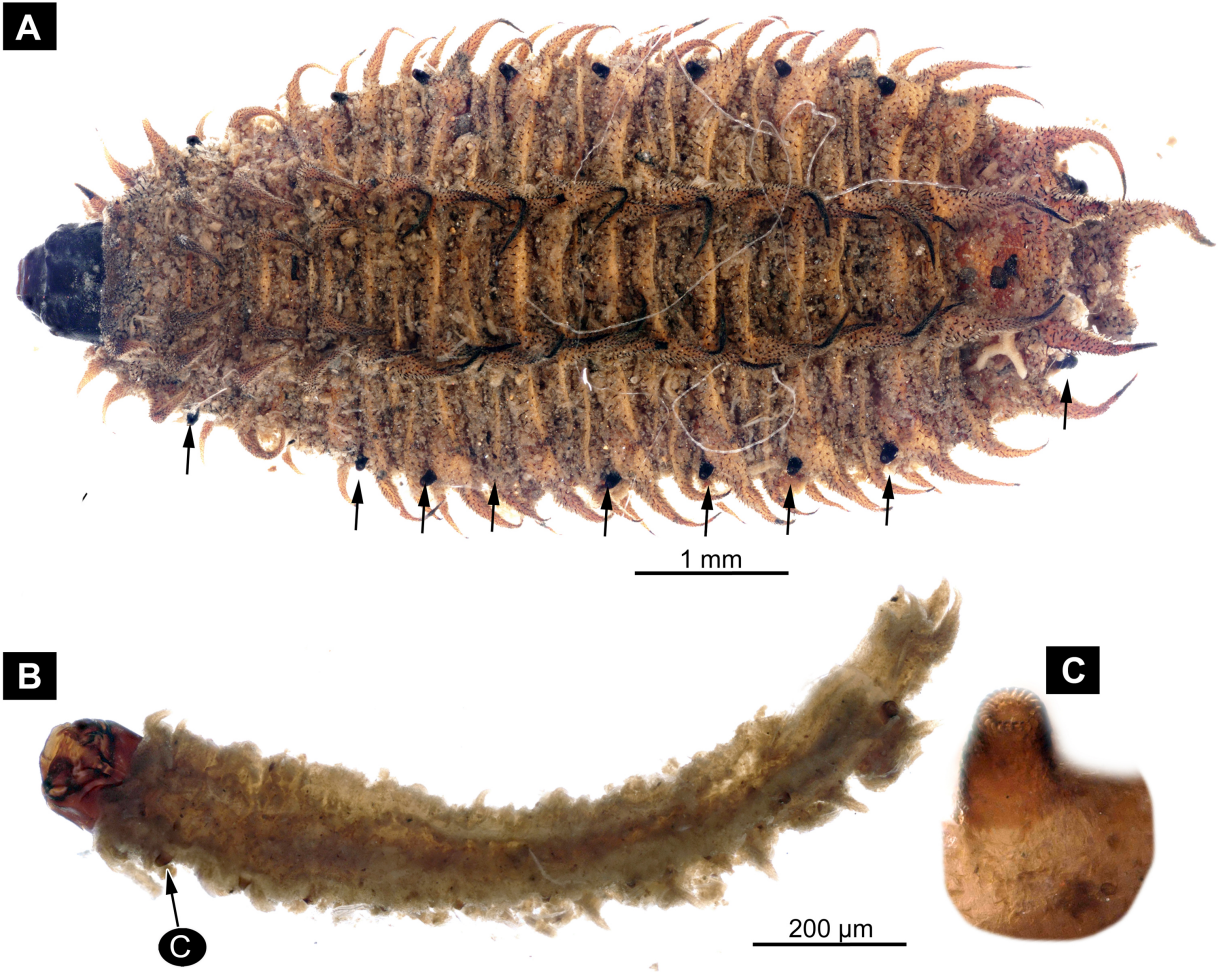
Extant larvae of Bibionidae. (A–C) *Penthetria funebris*Meigen, 1804, ZSM, no collection number assigned. (A) Fourth instar larva, habitus dorsal, arrows indicate the position of spiracles. (B) First instar larva, habitus ventral. (C) First instar larva, spiracle 1 (arrow in B).

*Systematic interpretation, general body features:* The general body shape, the absence of ambulatory legs on the thorax, as well as the spiracle arrangement is consistent with this larvae being an immature stage of the group Diptera. The larval specimen GPIH-0024 is interpreted to be clearly related to Bibionidae based on the following combination of characters (see Figs. 4A–4C; Figs. 5A, 5C): Head capsule fully sclerotized, posterior part of it is retracted into the prothorax; maxilla very short and stocky, with short and strong maxillary palp, head capsule black and shiny; eyes absent, antenna rudimentary; tracheal system holopneustic (“type 1” spiracles on the prothorax and metathorax, as well as on abdominal segments 1–7 & 9). Body heavily sclerotized, yet head capsule is sclerotized even more than the body. Prothorax is the longest segment of the trunk (Skartveit, 2017).

The very long and robust labium, the body with fleshy protuberances, bearing two rows of the protuberances dorsally and a single ecdysial scar on the posterior spiracle specimen, roughly resembles the condition in larvae of *Penthetria* (Meigen, 1804) (Hennig, 1968; Skartveit, 2002), an ingroup of Bibionidae (Figs. 4C, 5A–5C).

*Systematic interpretation, head structures:* The head capsule of the fossil larva is similar to that of larvae of Bibionidae. The antennae of the fossil larva are reduced as in larvae of Bibionidae. They are only represented by an undifferentiated conical piedestal in the fossil, similar to the condition in larvae of *Bibio* or *Penthetria* (both ingroups of Bibionidae; Figs. 5B, 5C). The maxilla of the fossil is robust, as it is in most larvae of Bibionidae. Yet, the larva differs in the structure of the maxillary palp (Figs. 4B, 4C): it is robust and cylindrical in general shape, similar to the representatives of *Penthetria* or *Bibio* (Figs. 4A–4C & 5A, 5C), but differs drastically from the representatives of both groups by bearing a conical outgrowth distally on the first element of the palp (Figs. 3A–3D, 4A, 4C & 5A–5C). This outgrowth is somewhat similar to the structure on the palpi of some extant larvae of Bibionidae. In particular, larvae of the ingroup of Bibionidae *Dilophus* possess large, conical sensillae on the palpi. The outgrowth of the fossil larva is however much larger proportionally to the maxilla than that of larvae of *Dilophus.* Also it is situated on the distal part of the first element, not on the second element of the palp as it is the case for *Dilophus* (Krivosheina & Mamaev, 1967).

Other larval forms of Bibionomorpha that possess large sensillae on the maxillary palps are larvae of fungus-gnats Mycomyinae (Mycetophilidae; (Krivosheina & Mamaev, 1967): fig 31:1, 31:6). In contrast to larvae of Mycomyinae, however, the outgrowths of the fossil larva are not articulated. We therefore argue that this is an unique character which is a putative autapomorphy of *Dinobibio hoffeinseorum* sp. nov.

The labium, in particular its proximal part, the mentum, is of the typical shape for larvae of Bibonidae (Figs. 3C, 3D), yet much broader and more robust than in any known larva of Bibionidae (s. Figs. 5A–5C). The labium is occupying up to 60% of the entire width of the ventral area of the head, while the labium in larvae of Bibionidae is much narrower, occupying about 20% of the ventral area of the head (Figs. 3C, 3D, Figs. 5B, 5C) (Skartveit, 2002). Mandibles and labrum are unavailable for a detailed examination due to being obscured by the other structures of the head.

*Systematic interpretation, trunk structures:* The general shape the body of the fossil larva is cylindrical with no parapods or other organs of locomotion (Figs. 2A, 2B). Fleshy protuberances are protruding from the cuticle of the abdomen of the fossil larva*.* Numerous larvae of Bibionidae are exhibiting this condition as well. In particular, cuticular protuberances are typical for larvae of *Plecia* or *Penthetria* (both ingroups of Bibionidae) (Figs. 5A–5C).

The protuberances of *D. hoffeinseorum* sp. nov. however differ from the protuberances of known larvae of Bibionidae, by their characteristic shape. The proximal attachment of the protuberances is relatively narrow expanding towards midlength, and narrowing towards conical distal end. (Figs. 2A, 2B). That character is differentiating *D. hoffeinseorum* sp. nov. from larvae of Bibionidae. In the latter the protuberances are simply tapering towards the tip (Fig. 2B). Additionally, the largest protuberances of *D. hoffeinseorum* sp. nov. are situated at the thorax and abdominal segments 1 and 2, in contrast to most larvae of Bibionidae, in which the length of the protuberances is increasing towards the posterior (Figs. 2A, 2B, Fig. S1). It is also possible, based on appearance, that the protuberances of *D. hoffeinseorum* sp. nov. are much more rigid than those of the known extant larvae of Bibionidae.

The tracheal system of the fossil larva is of the holopneustic type (“type 1”, 10 pairs of spiracles: one on the prothorax, one on the metathorax, one pair at abdominal units 1–7, and one pair at the trunk end; sensu Hennig, 1968). A holopneustic tracheal system is characteristic for larvae of Bibionidae.

The spiracle openings of the fossil larva are sitting on small elevated discs, representing a character state similar to that of some ingroups of Bibionidae. In larvae of Plecinae spiracle openings sit on conical outgrowths (Figs. 4A, 5B, 5C; cf. Skartveit, 2017). Most of the spiracles in the fossil are obscured by a silvery film, which, as it appears, formed by air, forced out from the tracheal system of the larva upon the entrapment in amber. Despite the obstruction of the view, the last tracheal spiracle pair (on abdominal unit 9) clearly has a single ecdysial scar, similar to larvae of *Penthetria* (Figs. 2A, 2B vs. Figs. 5A). In larvae of Bibionidae, the posterior spiracles are positioned posterior-laterally on the trunk end (Skartveit, 2002; Skartveit, 2017; Skartveit & Willassen, 1996). In contrast to them, the posterior spiracles of the new larva are situated at the anterio-dorsal part of the trunk end. Also, the posterior spiracles of the new larva are not larger than the other spiracles of the same larva. This is in contrast to known larvae of Bibionidae.

In, summary the fossil larva, here described as *D. hoffeinseorum* sp. nov. differs from any known larva of Bibionidae in three key characters: (1) a strong process at the distal end of element I of the maxilar palp, (2) a dorso-laterally position of spiracle 10 (on the trunk end); (Fig. S1); (3) protuberances of unique shape.

*Systematic interpretation, summary:* In fact, the larva described as *Dinobibio hoffeinseorum* sp. nov. is so different from known larval forms of Bibionidae concerning the general body pattern and the arrangement of the spiracles in the tracheal system, that it cannot be easily interpreted as an ingroup of Bibionidae (Skartveit, 2009; Skartveit, 2017). We can think of two possible explanations for the distinctiveness of the *D. hoffeinseorum* sp. nov. in comparison to larvae of Bibionidae (1) *D. hoffeinseorum* sp. nov. is not an ingroup of Bibionidae, but rather a sister species to the group. (2) *D. hoffeinseorum* sp. nov. is representing a highly derived branch of Bibionidae, that is now extinct.

Neither of these explanations can be conclusively excluded, until further specimens of *D. hoffeinseorum* sp. nov. will become available, but it is beyond any doubt that this new species is very distinct from the rest of the known larvae of Bibionomorpha. The larva of *D. hoffeinseorum* sp. nov. is exhibiting a curious mixture of traits, in the combination not known from any other larva of Diptera (cf. Kirk-Spriggs & Sinclair, 2017). It does however possess the characters known from larvae of Bibionidae and Mycetophilidae, yet in an unusual combination (i.e., see the discussion of the maxilla palpi element one outgrowth).

In fact, such “impossible” character combinations are quite common in the fossil record, representing an “experimental” phase of evolution, when a number of traits were independently evolving in different lineages (e.g., Haug et al., 2019). Occurrence of such an unusual combination of characters might be a natural result of the “Push of the Past” effect, caused by the fact that most of the lineages surviving until the present have done so as a result of the initial diversification (Budd & Mann, 2018). On the other hand the unique combination of characters in *D. hoffeinseorum* sp. nov. might be indicative of the active diversification in Bibionomorpha in the Eocene, which challenges the common view of the representatives of Insecta in the Baltic amber fossils as being “mostly modern” (Ponomarenko, 2003).

We would like to note that some colleagues have expressed reservations about describing new taxa based on immature stages. Yet, when it is possible to provide proper comparative diagnostics it is perfectly valid (according to ICZN) and also common to do this. In the present case the larva is so distinct that it is well possible to recognise the larva as a separate taxonomic entity.

**Table:** 

**Figure 6:**
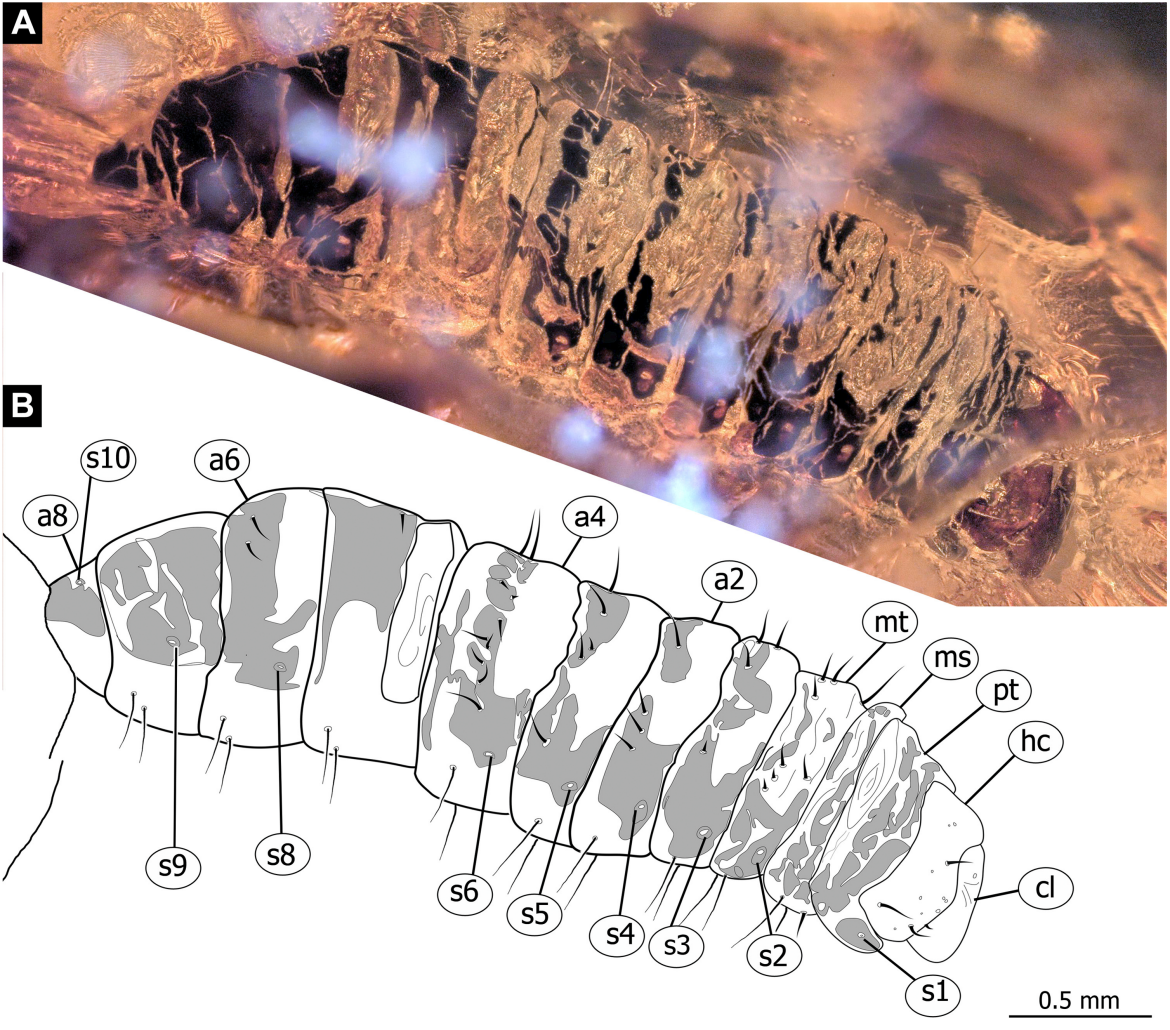
Fossil dipteran larva, *Pachyneura*, collection of GPIH, accession number (L-7617). (A) Habitus, dorsal. (B) Schematic drawing of habitus, dorsal. Abbreviations: a2–a8, abdominal segment 2–8; cl, clypeus; hc, headcapsule; ms, mesothorax; mt, metathorax; pt, prothorax; s1–s10, spiracle 1–10.

**Figure 7:**
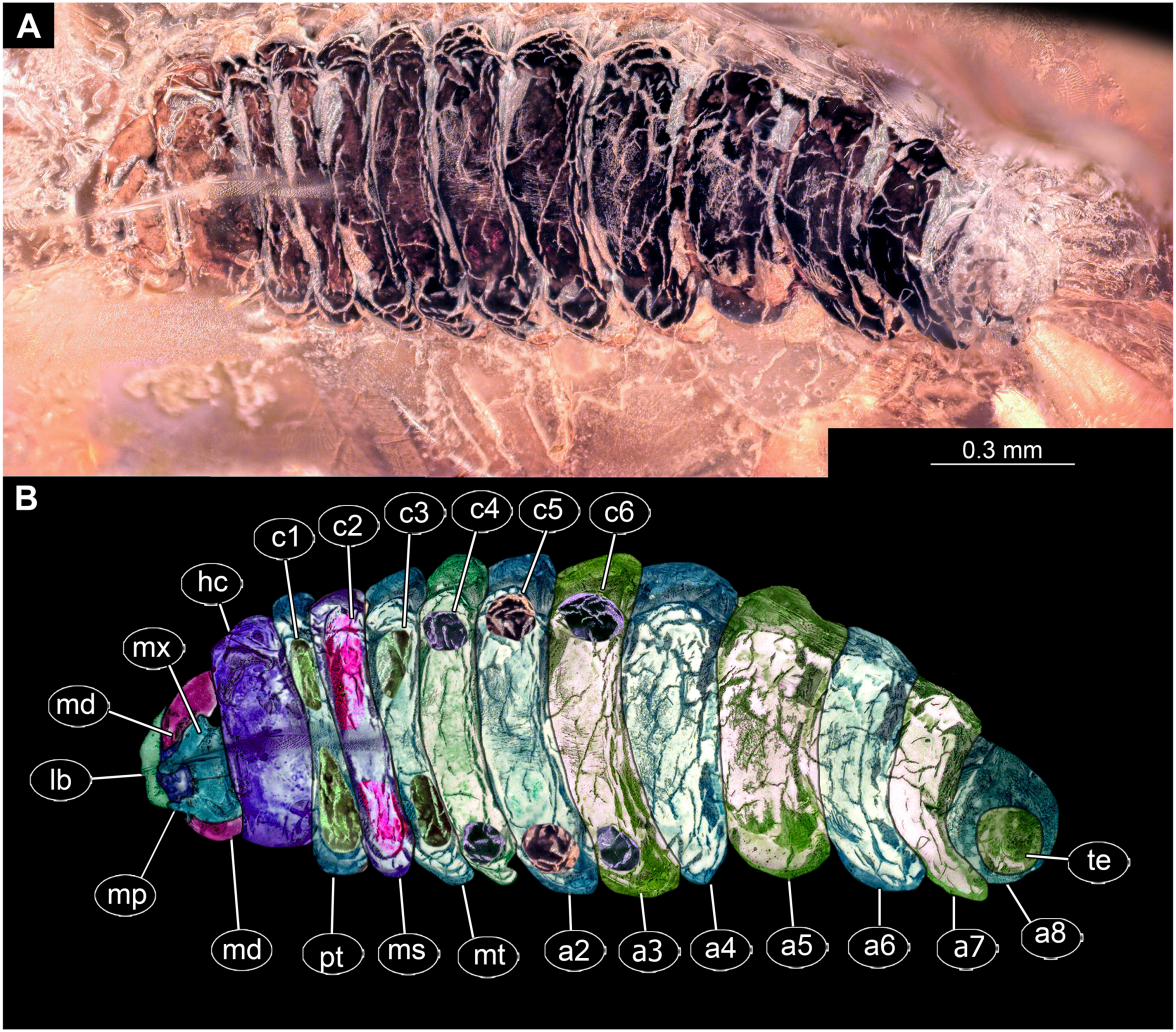
Fossil dipteran larva, *Pachyneura*, collection of GPIH (L-7617). (A) Habitus, ventral. (B) Colored version of A. Abbreviations: a1–a8, abdominal segments 1–8; c1–c6, creeping welts 1–6; hc, headcapsule; lb, labrum; md, mandibles; mp, maxillar palp; ms, mesothorax; mt, metathorax; mx, maxilla; pt, prothorax; te, trunk-end.

**Figure 8:**
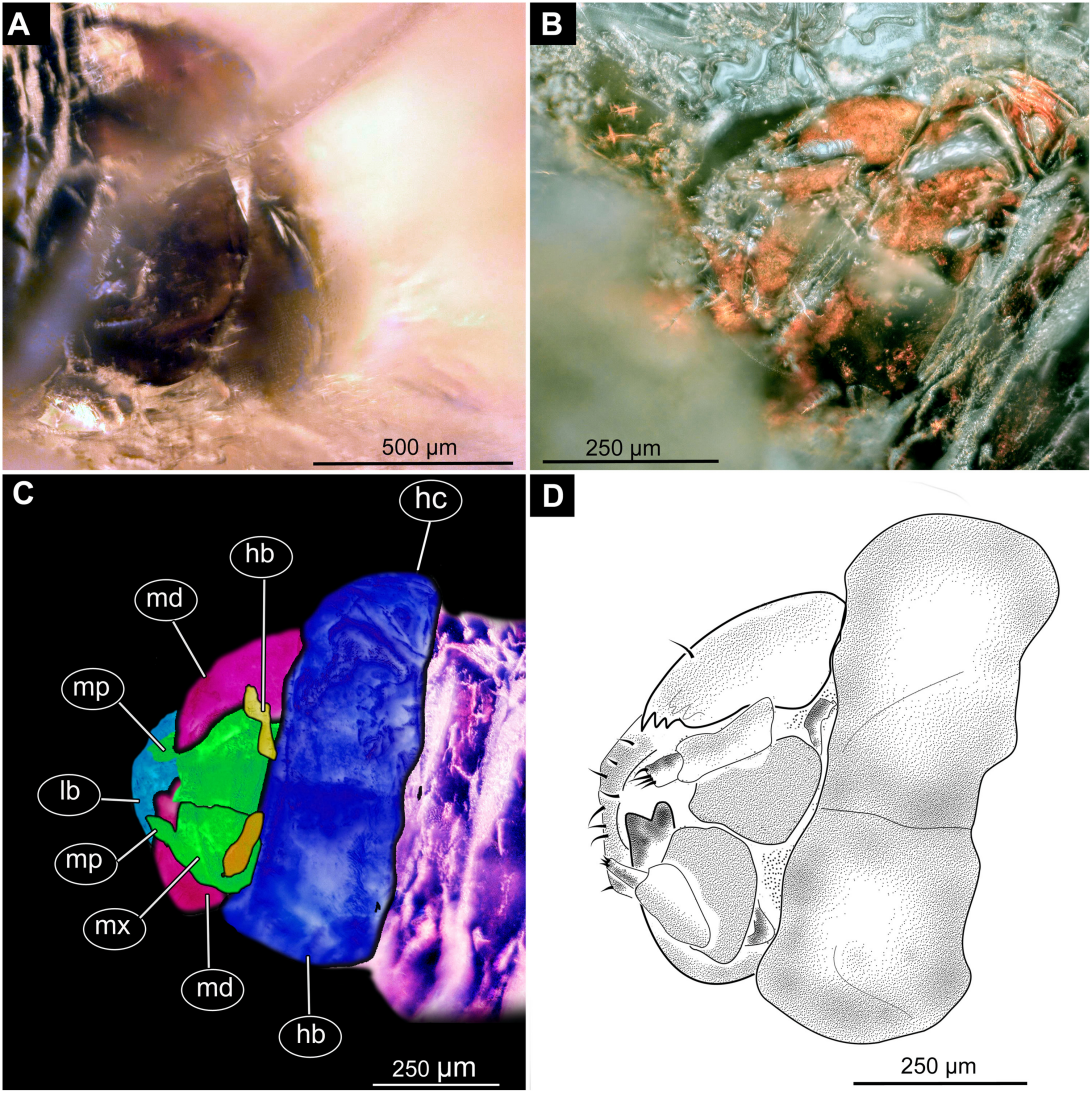
Fossil dipteran larva, *Pachyneura*, collection of GPIH, accession number (L-7617). (A) Head capsule, dorsal view. (B) Head capsule, ventral view. (C) Colored version of B. (D) Head capsule, ventral view, schematic drawing. Abbreviations: hb, hypostomal bridge; hc, head capsule; lb, labrum; md, mandibles; mp, maxilary palps; mx, maxillae.

Pachyneuridae + Hesperinidae (unnamed monophyletic group, Krivosheina, 2012)
Pachyneuridae Schiner, 1864
*Pachyneura*Zetterstedt, 1838
(Figs. 6A, 6B, 7A, 7B, 8A–8D)

*Material*: A single fossil larva from the collection of Carsten Gröhn, which is now part of the CeNak collection (Hamburg) with the collection number GPIH-L-7516. Specimen moderately well preserved, with posterior parts of the trunk obscured by cracks, lateral view not available. It appears that the specimen was desiccated before being encased in amber as evident from its somewhat distorted appearance.

*Syninclusions:* “Stellate hairs” (oak leaf trichomes).

Description:

**Habitus.** Medium sized larva with a dorso-ventrally flattened, spindle-shaped body. Total length 2.8 mm. Body differentiated into presumably 20 segments, ocular segment plus 19 post-ocular segments (Fig. 6A, 6B, 7A, 7B).

**Figure 9:**
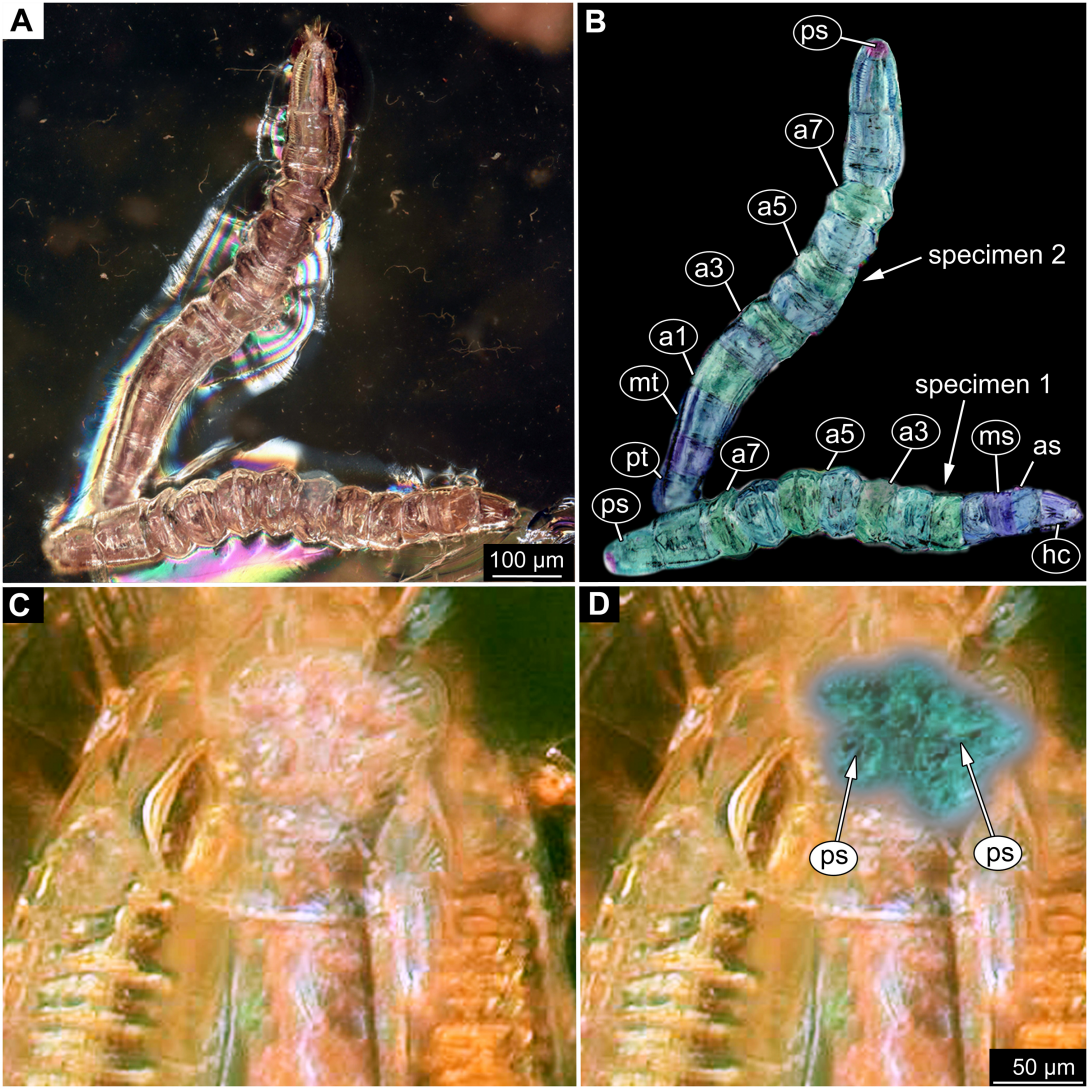
Fossil dipteran larva, *Mycetobia*, DEI, accession number Dip-00640. (A) Habitus, dorsal view. (B) Colored version of A. (C) Posterior spiracles, specimen 2 of B. (D) Colored version of C. Abbreviations: a2–a8, abdominal segments 2–8; as, anterior spiracle; hc, head capsule; ms, mesothorax; mt, metathorax; ps, posterior spiracle; pt, prothorax.

**Head.** Ocular segment and post-ocular segment 1–5 (presumably) forming a distinct capsule (head capsule). Head capsule wider than long. Hind part of head capsule not retracted into anterior trunk. Dimensions of head capsule: 450 µm long, 770 µm wide. Surface of head capsule smooth and glossy. Ocular segment without apparent stemmata (larval eyes) (Figs. 9A–9D).

**Ocular segment** recognizable by its appendage derivative, clypeo-labral complex. Clypeus (clypear sclerite) roughly rectangular, 200 µm long, 380 µm wide. Labrum small, weakly sclerotized (Fig. 8C).

**Post-ocular segment 1** without externally recognizable structures. Antenna not discernible, probably reduced. (Fig. 8A).

**Post-ocular segment 2** (intercalary segment) without externally recognizable structures (Fig. 8C).

**Post-ocular segment 3** recognizable by its pair of appendages, mandibles. Mandible total length 220 µm, with 3 strong teeth on the apex, apical and subapical teeth sub-equal (all ca. 22 µm in length), molar tooth shorter (16 µm) (Fig. 8C).

**Post-ocular segment 4** recognizable by its appendage, maxilla [maxillula]. Maxilla massive, organized into proximal part and distal part or palp [endopod]. Very proximal region with sclerite (hypostomal bridge). Further distal proximal part differentiated into two lobes, outer lobe and inner lobe. Inner lobe wth, possible lacinia [endite]. Possible lacinia rectangular in outline, 100 µm long, 70 µm wide. Palp arising from outer lobe, cylindrical, with two elements (palpomeres). Element 1 104 µm long, element 2 45 µm long, with 4 hair-like setae distally (Fig. 8C).

**Post-ocular segment 5** recognizable by its appendages, forming the labium [conjoined left and right maxillae]. Labium largely obscured by the large possible lacinia (Fig. 8C).

**Trunk** with 12 visible units, interpreted as 3 thorax segments plus 8 abdominal units and a trunk end, representing a conjoined structure of possibly undifferentiated abdominal segments (9–11?) (Fig. 6A, 6B; 7A, 7B). Trunk widest at about half of the length with 910 µm, diameter decreasing posteriorly to 280 µm. Trunk with elevated ridges (possible creeping welts) at units 1-6 (three thorax units, and first three units of the abdomen). Trunk surface with numerous small spines. Trunk bears 10 pairs of spiracles (openings of the tracheal system). Spiracles surrounded by lightly-colored fields on the otherwise heavily sclerotized trunk units. Spiracles appear to have single ecdysial scars.

**Thorax** consists of three segments, pro-, meso- and metathorax.

**Prothorax** 80 µm long. Prothorax bears a pair of large spiracles. Prothorax subdivided into two parts by annular constriction.

**Mesothorax** 95 µm long. No spiracle openings present. Mesothorax bears two lateral setae (ca. 70 µm long ) on each side of the segment.

**Metathorax** 90 µm long. Metathorax bears two groups of dorsal setae (20–40 µm long), and two lateral setae (ca 70 µm long ) on each side of the segment. Metathorax bears a pair of spiracles.

**Abdomen (posterior trunk)** Abdominal units progressively increasing in dorsoventral aspect towards the posterior part of the body, until reaching midlength of the abdomen, then decreasing again, towards the trunk end.

 Abdominal units 1–4, 6 bear two groups of dorsal setae (20-40 µm long), and two lateral setae (ca 70 µm long ) on each side of the segment. Units 1–8 each bearing a pair of spiracles laterally.

**Abdominal unit 5** (abdomen segment 5) bears two lateral setae (ca 70 µm long ) on each side of the segment.

**Abdominal unit 7** (abdomen segment 7) bears two lateral setae (ca 70 µm long ) on each side of the segment.

**Trunk end** (undifferentiated abdomen segments 9–11?) obscured by cracks.

*Systematic interpretation:* The general body shape, as well as absence of ambulatory legs on the thorax, and the spiracle arrangement is consistent with this larva being an immature stage of Diptera. Numerous characters indicate that this is a larval form of Bibionomorpha: The larva possesses a very wide head capsule. The body as a whole is somewhat flattened dorso-ventrally, bearing six pairs of small ridges on the ventral side of the first six segments of the trunk (Figs. 6A, 6B, 7A, 7B).

The specimen is unusual by the combination of a holopneustic tracheal system (“type 2”: spiracles (Hennig, 1968) on the prothorax, metathorax and abdominal segments 1–8, Fig. 6B), presence of long setae on the abdomen, the head capsule being wider than long (Figs. 6A, 6B, 7A, 7B), prothorax being subdivided by a transversal furrow into the two rings (Figs. 6B, 7B). All spiracles are surrounded by a lighter colored area, in contrast to the more sclerotized parts of the segments. There are no other known larvae of Bibionomorpha with this state of characters. It is possible however that the lighter areas are actually taphonomic artefacts, caused by air extrusions from the tracheal system upon the entrapment in amber.

The tracheal system with ten pairs of spiracles on the pro- and metathorax as well as on abdominal units 1–8 (Fig. 6B), is a synapomorphy of the bibionomorphan ingroups Pachyneuridae + Hesperinidae (Krivosheina, 2012). The fossil is however distinct from larvae of Hesperinidae by bearing a large number of long setae (up to 70 µm long) on the abdominal units. Larvae of Hesperinidae possess only very short setae (Krivosheina, 2012). *Pachyneura* (only ingroup of Pachyneuridae sensu Paramonov & Salmela 2015) includes two species, *Pachyneura fasciata* Zetterstedt, 1838 and *P. oculata* Krivosheina & Mamaev, 1972. Due to the suboptimal preservation of the larva, we decided not to formally describe a new species, as the resulting holotype would be not optimal for future comparative work.

In general, based on the combination of morphological characters, the larva appears to be a typical larva of *Pachyneura* (Pachyneuridae see Paramonov & Salmela 2015). This is the first and thus oldest fossil record of Pachyneuridae sensu Paramonov & Salmela (2015). Cramptonomyiidae, the sister group of Pachyneuridae+Hesperinidae, is present in the fossil record with representatives of its ingroups *Tega* Blagoderov, Krzeminska & Krzeminski, 1993 and *Pivus* Blagoderov, Krzeminska & Krzeminski, 1993 from Upper Jurassic respectively the Lower Cretaceous of Asia (Blagoderov, Krzeminska & Krzeminski, 1993).

**Table:** 

Anisopodidae Knab, 1912
*Mycetobia*Meigen, 1818

*Material:* 53 specimens of larvae and pupae in total were examined, see Table 1 for a complete list of the material. We were not able to distinguish distinct morphotypes for the larvae of *Mycetobia*, while for the pupae three distinct morphotypes are apparent.

**Table:** 

Larvae
(Figs. 9A–9D; 10A–10E; Figs. S2–S10)

*Material:* see Table 1 and Figs. 9A–9D; 10A–10E, Figs. S2–S10.

**Figure 10:**
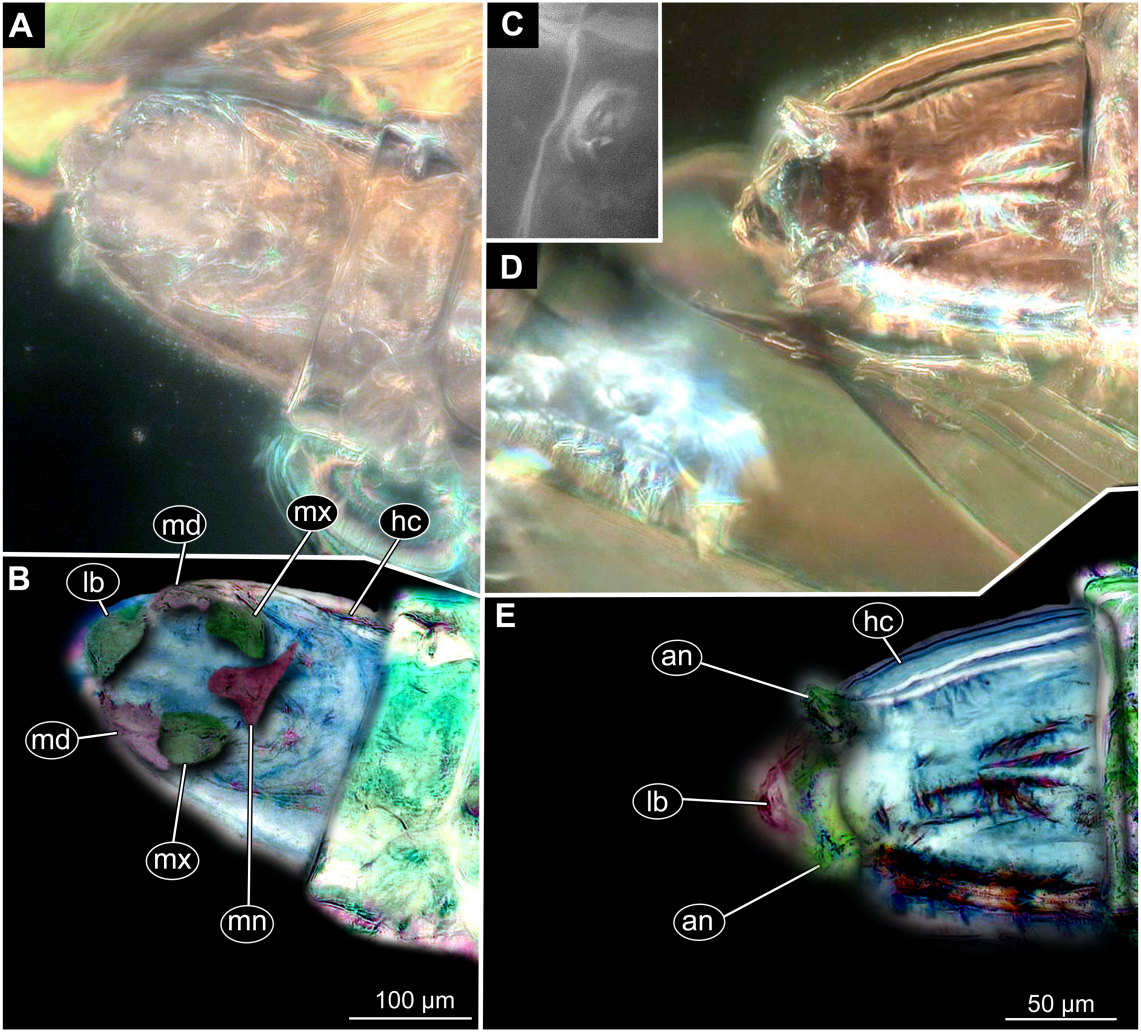
Fossil dipteran larva, *Mycetobia*, DEI accession number Dip-00640, specimen 1 of Fig. 9. (A) Head capsule, dorsal view. (B) Anterior spiracle. (C) Colored version of A. (D) Head capsule, ventral view. (E) Colored version of D. Abbreviations: an, antenna; as, anterior spiracle; hc, head capsule; lb, labrum; md, mandibles; mn, mentum; mp, maxilar palps; mx, maxillae; ps, posterior spiracle.

Description:

**Habitus**. Medium sized larva with roughly vermiform body (Figs. 9A, 9B). Total length 1.8–10.2 mm (all life stages; see Table 2 for the summary of the morphometrics of the studied specimens) (Figs. 10A, 10B).

**Table 2:** Morphometry of the fossil *Mycetobia* larvae from Baltic and Bitterfeld ambers. Number in the parentheses after accession number indicates number of the *Mycetobia* syninclusion (if more than one in the same piece of amber). “L”- length, “W”-width.

**Acession number**	**L total, µm**	**head L, µm**	**head W, µm**	**larval stage**
Dip-00640 (1)	2676.177	145.201	105.129	1
GPIH-0247/8	3346.186	165.624	115.993	1
PED-4748(3)	2283.494	99.005	87.693	1
Dip-00656	2067	166	115	1
Dip-00640 (2)	2151.442	186.238	162.515	2
Dip-00640(3)	2693.354	209.082	178.836	2
Dip-00640 (4)	2405.655	171.311	155.919	2
GPIH-3706 W	2957.863	190.825	180.487	2
BI2350	3909.86	235.719	155.103	2
GPIH-0247(7)	3034.273	195.118	166.481	2
PED-4748(1)	5048.093	309.328	171.883	2
PED-4970	4591.883	233.701	156.178	2
Dip-00656(2)	2784	181	192	2
Dip-00655(1)	2364	139	145	2
Dip-00649(1)	5166	178	181	2
GPIH-0247(9)	3	320.337	259.113	3
PED-4748(2)	5207.932	388.551	246.06	3
PED-4748(4)	10222.51		191.139	3
PED-4965	7027.351	319.331	218.775	3
PED-5695	5503.7	284.294	230.87	3
Dip-00639	7609.245	306.751	295.106	3
Dip-00658	8139	376	239	3
Dip-00656 (1)	5693	266	240	3
Dip-00655(2)	2344	225	227	3
Dip-00649(2)	8385	352	277	3
GPIH-0247(2)	3929.665	512.765	418.808	4
GPIH-0247(1)	5328.197	NA	NA	NA
GPIH-0247(3)	4150.859	NA	NA	NA
GPIH-0247(4)	4898.89	NA	NA	NA
GPIH-0247(5)	1819.851	NA	NA	NA
GPIH-0247(6)	3486.205	NA	NA	NA
GPIH-l-7592(1)	7194.75	NA	NA	NA
GPIH-l-7592(2)	6096.312	NA	NA	NA
GPIH-l-7592(3)	5701.261	NA	NA	NA
GPIH-l-7592(4)	6454.761	NA	NA	NA
GPIH-l-7592(5)	4017.086	NA	NA	NA

Body differentiated into presumably 20 segments, ocular segment plus 19 post-ocular segments (Figs. 9A–9D; 10A–10E).

**Head.** Ocular segment and post-ocular segment 1–5 (presumably) forming distinct capsule (head capsule). Head capsule longer than wide. Head capsule well developed, fully sclerotized dorsally, partially sclerotized ventrally. Hind part of head capsule not retracted into anterior trunk. Dimensions of head capsule: length 99–512 µm (*n* = 25, all life stages), width 85–420 µm (*n* = 26, all life stages). Surface of head capsule smooth and glossy.

**Ocular segment** without apparent stemmata (larval eyes). Ocular segment recognizable by its appendage derivative, clypeo-labrum complex (Figs. 10A, 10D).

**Post-ocular segment 1** recognizable by its appendages, antennae [antennulae]. Antenna represented by a single, cone-shaped element bearing a mushroom-like sensillum distally (Figs. 10A, 10B, 10D, 10E).

**Post-ocular segment 2** (intercalary segment) without externally recognizable structures (Figs. 10A, 10B).

**Post-ocular segment 3** recognizable by its pair of appendages, mandibles. Mandible divided into large, unsclerotized proximal portion, and heavily sclerotized distal portion, bearing numerous teeth (Figs. 10A, 10B, 10D, 10E).

**Post-ocular segment 4** recognizable by its appendage, maxilla [maxillula]. Maxilla massive, organised into proximal part and distal part or palp [endopod]. Maxilla fleshy, very weakly sclerotized, only general outline visible. Proximal part differentiated into two lobes, outer lobe and inner lobe. Palp small, stump-like (Figs. 10A, 10B).

**Post-ocular segment 5** recognizable by its appendages, forming the labium [conjoined left and right maxillae]. Labium, especially proximal part (mentum), narrow and weakly sclerotized, trapezium-shaped. No distal structures (palpi) apparent. Posterior tentorial pits (external anchor point of the internal skeleton of the head capsule) present (Figs. 10A, 10B).

**Trunk.** Trunk composed of 11 visible units: pro-, meso- and metathorax, 7 abdominal units and the trunk end. Trunk worm-like, units sub-equal in diameter (Figs. 9A, 9B). Trunk lacks parapodia and/or creeping welts. Trunk bears two pars of spiracles: one on prothorax (Fig. 9C) and one on trunk end (Figs. 9C, 9D).

**Thorax** consists of three segments, pro-, meso- and metathorax.

**Prothorax** bears small, cone-shaped, anterior spiracles situated on posterolatero-dorsal surface. Prothorax subdivided into two unequal parts by annular constriction.

**Meso- and** **metathorax** subequal to prothorax in length, but without annular constriction (Figs. 9A, 9B).

**Abdomen** (posterior trunk) with abdominal units cylindrical, roughly equal to each other in diameter.

**Abdominal units 1–7** subdivided into two unequal parts by annular constriction

**Trunk end** (undifferentiated abdomen segments 8–11?) subdivided into three unequal parts by two annular constrictions, with perianal shield (modified area of the last unit surrounding the anal aperture) on the ventral side. Trunk end bears posterior spiracles situated on the medio-postero-dorsal surface of the unit. Spiracular field surrounded by 5 short lobes, bearing no apparent hairs (Figs. 9A, 9D).

Systematic interpretation:

The general body shape, as well as the absence of ambulatory legs on the thorax, and the spiracle arrangement are consistent with these larvae being immature stages of the group Diptera. The larvae furthermore show a distinct combination of characters: slender, vermiform body; head sclerotized; dorsal part more strongly sclerotized than ventral one; mandible consists of fleshy proximal part more heavily sclerotised distal part; prothorax and abdominal units 1–7 each subdivided into two unequal parts by an annular constriction; respiratory system amphipneustic; anterior spiracles on a small cone on prothorax; posterior spiracles on spiracular field, on the posterior of the trunk; trunk end covered by a perianal shield; the trunk end further subdivided into three parts.

**Figure 11:**
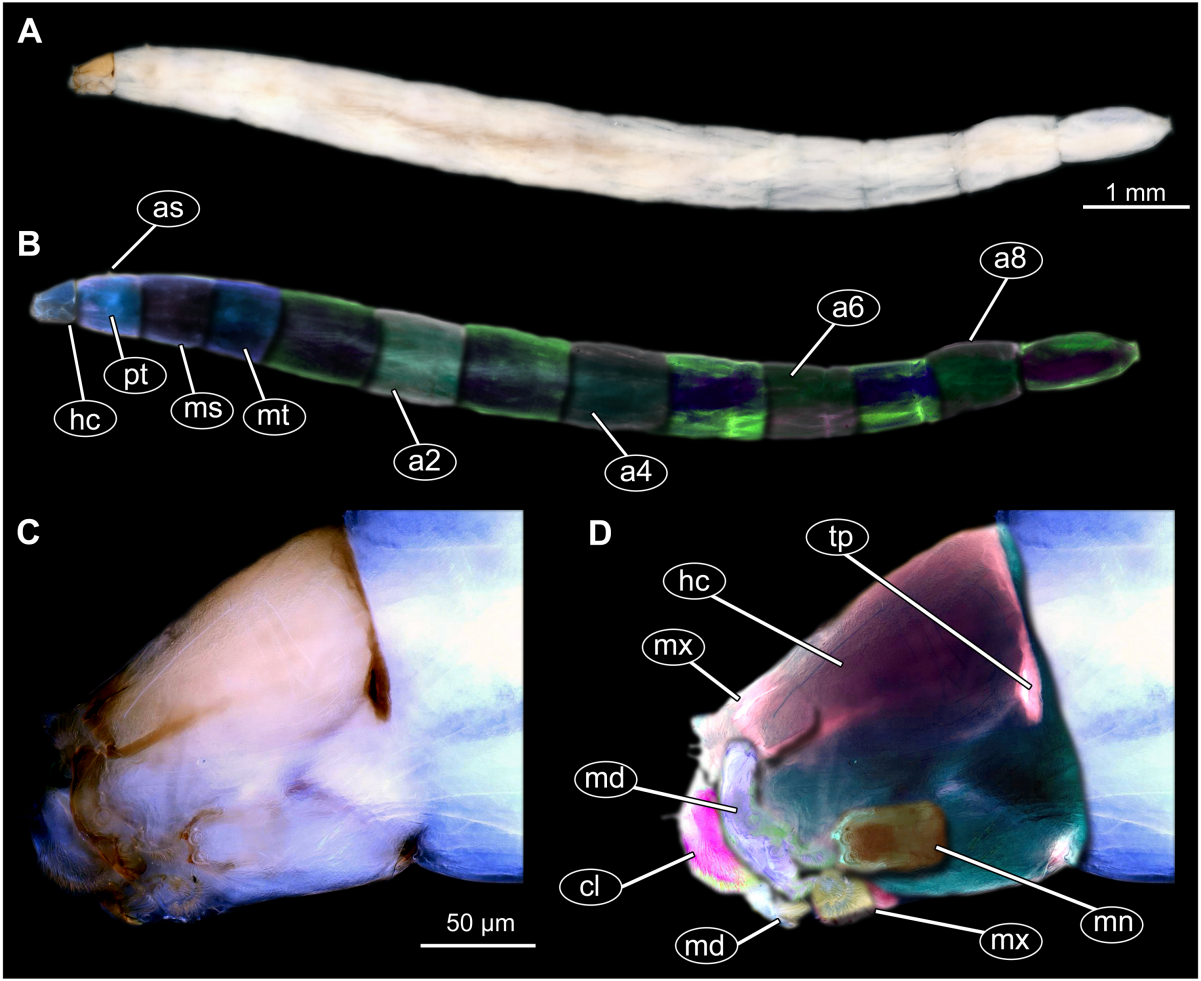
Extant dipteran larva, *Mycetobia pallipes*Meigen, 1818, ZSM, no collection number assigned. (A) Habitus, lateral. (B) Colored version of A. (C) Head capsule, lateral view. (D) Colored version of C. Abbreviations: a2–a8, abdominal segment 2–8; as, anterior spiracle; hc, head capsule; md, mandible; mn, mentum; ms, mesothorax; mt, methathorax; mx, maxillae; pt, prothorax; tp, posterior pit of tentorium.

This character combination matches the condition in larvae of Anisopodidae (window gnats). Furthermore the fossil larvae show a spiracular disc surrounded by only very short lobes and weak setae (Figs. 9A–9D, 11A–11D). This character is an autapomorphy of *Mycetobia* (ingroup of Anisopodidae).

**Table:** 

Pupae
Morphotype 1
(Figs. 12A, 12B; Figs. S11–S26

*Material:* see Table 1 and 12A, 12B; Figs. S11–S26

Description:

**Figure 12:**
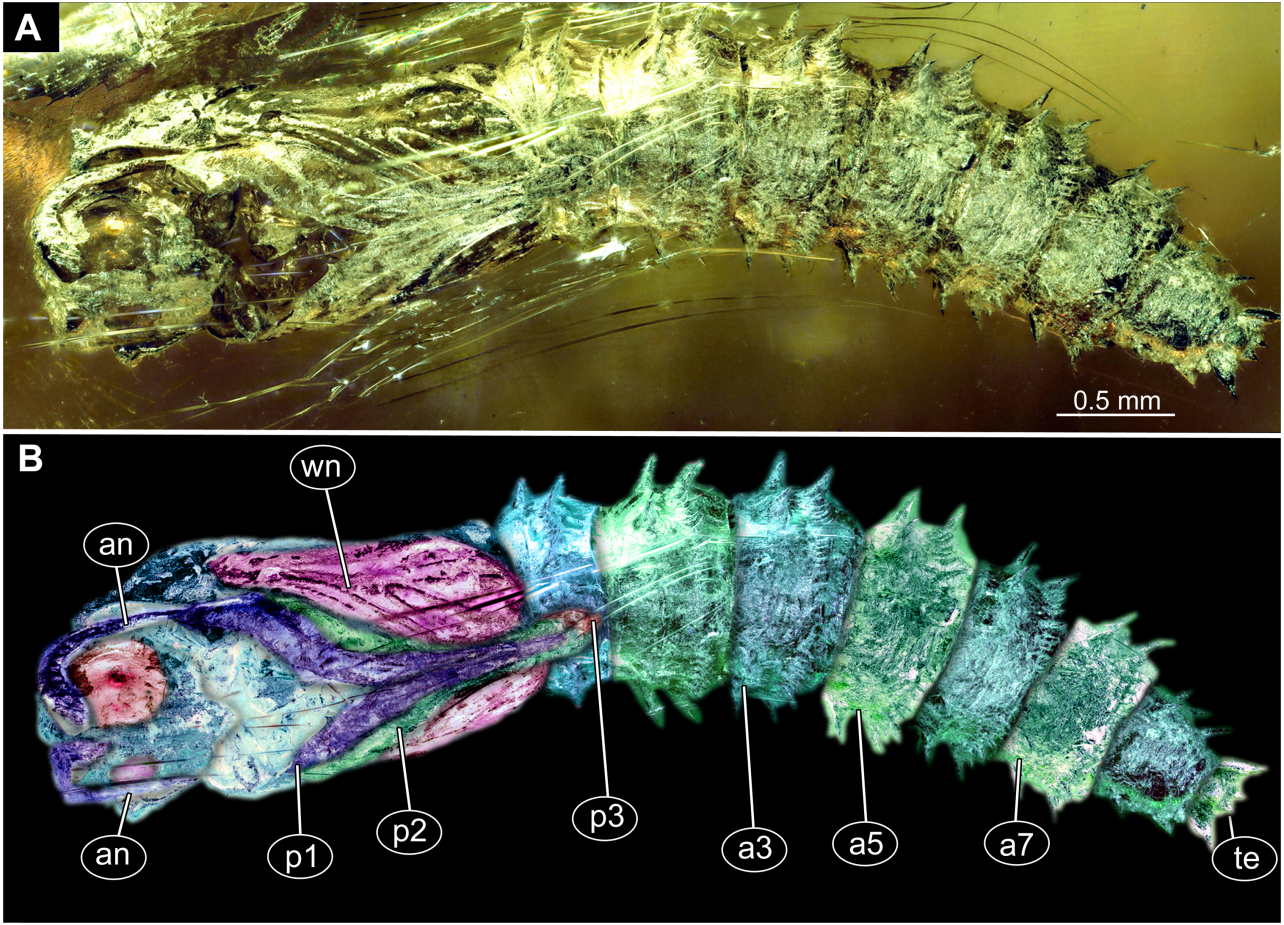
Fossil pupa, *Mycetobia connexa* (Mycetobia “morphotype 1”), GPIH, collection number 1851-DN. (A) Habitus, ventro-lateral view. (B) Colored version of A. Abbreviations: a3–a7, abdominal segments 3–7; an, antennae; fs, frontal setae; p1, front legs; p2, midlegs; p3, hind legs; te, trunk-end; wn, wings.

**Habitus.** Medium sized pupa, with generally comma-shaped body in lateral view (Figs. 12A, 12B; Figs. S11–S26). Pupae colored roughly in the same colour as the matrix of the amber. Total length 2.7–5.1 mm long (*n* = 14). See Table 3 for a summary of the morphometrics. Body differentiated into presumably 20 segments, ocular segment plus 19 post-ocular segments. Ocular segment and post-ocular segment 1–8 (presumably) forming a single globose unit (Figs. 12A, 12B; Figs. S11–S26).

**Table 3:** Morphometry of the fossil *Mycetobia* pupae from Baltic and Bitterfeld ambers.

**Accession number**	**length, µm**	**parameter**	**Morphotype**
Dip-00655	1777.074	abdomen	morphotype 1
Dip-00655	1013.289	thorax+head	morphotype 1
Dip-00655	2679.723	total	morphotype 1
Dip-00655	2484.743	abdomen	morphotype 1
Dip-00655	1614.781	thorax+head	morphotype 1
Dip-00655	3842.338	total	morphotype 1
Dip-00652	362.857	thorax+head	morphotype 3
Dip-00652	527.673	abdomen	morphotype 3
Dip-00652	826.356	total	morphotype 3
Dip-00653	2420.659	abdomen	morphotype 1
Dip-00653	1779.554	thorax+head	morphotype 1
Dip-00653	3919.83	total	morphotype 1
GPIH-1851DN	3021.056	abdomen	morphotype 1
GPIH-1851DN	2266.877	thorax+head	morphotype 1
GPIH-1851DN	5059.427	total	morphotype 1
Dip-00641	2340.723	abdomen	morphotype 1
Dip-00641	1624.223	thorax+head	morphotype 1
Dip-00641	3876.262	total	morphotype 1
Dip-00650	320.106	thorax+head	morphotype 3
Dip-00650	645.888	abdomen	morphotype 3
Dip-00650	864.21	total	morphotype 3
Dip-00660	2935.409	abdomen	morphotype 1
Dip-00660	1924.388	thorax+head	morphotype 1
Dip-00660	4238.969	total	morphotype 1
Dip-00661	3647.714	abdomen	morphotype 1
Dip-00661	2220.334	thorax+head	morphotype 1
Dip-00661	5861.01	total	morphotype 1
Dip-00657	2310.204	abdomen	morphotype 1
Dip-00657	1453.298	thorax+head	morphotype 1
Dip-00657	3835.301	total	morphotype 1
GPIH-N-7095.	2154.926	abdomen	morphotype 1
GPIH-N-7095.	1710.244	thorax+head	morphotype 1
GPIH-N-7095.	3761.555	total	morphotype 1
Dip-00659	2466.357	abdomen	morphotype 1
Dip-00659	1697.196	thorax+head	morphotype 1
Dip-00659	3744.385	total	morphotype 1
Dip-00651	2187.597	abdomen	morphotype 1
Dip-00651	1543.324	thorax+head	morphotype 1
Dip-00651	3343.985	total	morphotype 1
AKBS-00071	2490.055	abdomen	morphotype 1
AKBS-00071	1784.352	thorax+head	morphotype 1
AKBS-00071	3630.701	total	morphotype 1
PED-4395	2081.768	abdomen	morphotype 1
PED-4395	1561.697	thorax+head	morphotype 1
PED-4395	3528.726	total	morphotype 1
PED-4866	2596.66	thorax+head	morphotype 2
PED-4866	3041.19	abdomen	morphotype 2
PED-4866	5379.843	total	morphotype 2
PED-4998	2882.949	abdomen	morphotype 1
PED-4998	2174.641	thorax+head	morphotype 1
PED-4998	4811.619	total	morphotype 1
GPIH-L-7514	1826.663	thorax+head	morphotype 2
GPIH-L-7514	2936.171	abdomen	morphotype 2
GPIH-L-7514	4858.746	total	morphotype 2

**Ocular segment** recognizable by its appendage derivative, clypeo-labrum complex and pair of large compound eyes. Labrum oval, slightly invaginated, membranous. Clypeus continuous with labrum (Figs. 12A, 12B, Fig. S21). Frons (frontal sclerite) with a pair of short setae, situated on top of small conical warts. Setae of frontal sclerite longer than warts (Figs. 12A, 12B; Fig. S21).

**Post-ocular segment 1** recognizable by its appendages, antennae [antennulae]. Antenna consisting of 16 elements. Antennae moderately long, following the dorso-posterior outlines of the compound eyes.

**Post-ocular segment 2** (intercalary segment) without externally recognizable structures (Figs. 12A, 12B; Fig. S21).

**Post-ocular segment 3** without externally recognizable structures (mandibles) (Figs. 12A, 12B; Fig. S21).

**Post-ocular segment 4** recognizable by its appendage, maxilla [maxillula]. Maxilla with proximal part (non-serrated “lacinia”) and distal part, palp [endopod] (Figs. 12A, 12B; Fig. S21).

**Post-ocular segment 5** recognizable by its appendages, forming the labium [conjoined left and right maxillae]. Proximal parts of labium membranous, bears labial palps (Figs. 12A, 12B; Fig. S21).

**Thorax** consists of three segments, pro-, meso- and metathorax. Each bears a pair of (ambulatory) appendages (fore-, mid- and hind legs). Wings on mesothorax; halterae on metathorax. Thorax segments forming a single semiglobose structure, closely enveloping the head (Figs. 12A, 12B; Fig. S21).

**Ambulatory appendages** (legs) U-shaped folded, running between the wings: mid- and hind legs terminating above the mid-length of the first posterior trunk (abdomen) unit. Ambulatory appendages curving between the wing tips, and then, diverging again after passing the tips of the wings (Figs. 12A, 12B; Fig. S21, S25, S26). All ambulatory appendages subdivided into the elements: coxa, trochanter, femur, tibia and tarsus (subdivided into 5 elements).

**Prothorax** bears thoracic horns (modified spiracle 1). Thoracic horns club shaped, situated posterior to the eyes on the dorsal surface of the prothorax (Figs. 12A, 12B). Prothorax bears first thoracic appendage pair (forelegs). Forelegs with femur and tibia forming a U-shaped loop, with anteriormost point of the loop reaching the level at which the maxillae arise.

**Mesothorax** bears a pair of wings. Base of the wing aligned with the tip of the antennae. Midlegs underlying the forelegs, reaching beyond the tip of the wing.

**Metathorax** with a pair of spiracles. Hind legs underlying the forelegs and midlegs, reaching beyond the tip of the wing (Figs. 12A, 12B).

Length of head and thorax combined 1.0–2.3 mm (*n* = 14). Abdomen 1.8–3.6 mm long (*n* = 14).

**Abdomen (posterior trunk).** With 9 units.

**Abdominal units 1–8** each bearing two rings of strong hooklets. 12 hooklets in the first ring, circa 70 hooklets in the second ring (Figs. 12A, 12B). Abdominal units 2–8 each bearing a pair of small spiracles (Figs. 12A, 12B, Fig. S21).

**Trunk end** (undifferentiated abdomen segments 9–11?) bears a pair of the lateral expansions (anal lobes) 8+2 hooklets. Hooklets arranged in 2 rings, two additional hooklets located on the anal lobes (Figs. 12A, 12B; Fig. S21). Abdomen length 1.7–3.6 mm (*n* = 14).

**Table:** 

**Figure 13:**
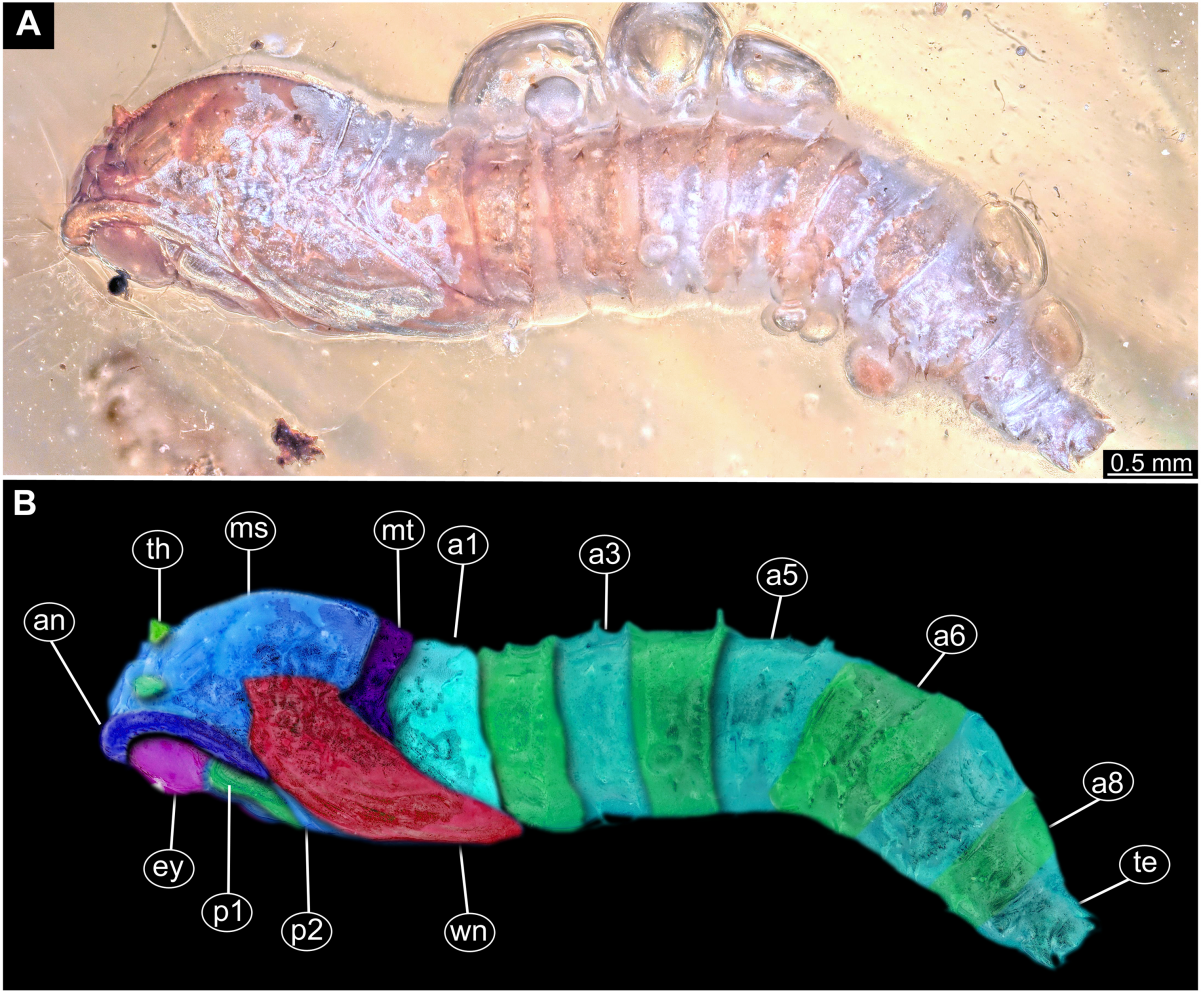
Fossil pupa, *Mycetobia* “morphotype 2”, PED, collection number PED-4866. (A) Habitus, lateral view. (B) Colored version of A. Abbreviations: a1–a8, abdominal segments 1–8; an, antennae; ey, eyes; ms, mesothorax; mt, metathorax; p1, front legs; p2, midlegs; p, prothorax; te, trunk-end; th, thoracic horns; wn, wings.

*Mycetobia* pupae morphotype 2
(Figs. 13A, 13B, Fig. S27)

*Material:* This morphotype is represented by two pupae in our material; one specimen in the amber piece GPIH-7514 (originally from the collection of Carsten Gröhn), a second specimen in the amber piece PED-4866.

Description:

**Habitus**. Medium sized pupa, with generally comma-shaped body in lateral view. Pupa in whitish-green to brown colors. Total length 4.3–5.3 mm long (*n* = 2).

Body differentiated into presumably 20 segments, ocular segment plus 19 post-ocular segments. Anterior part of the body composed of head and thorax, visible as a single globose structure (Figs. 13A, 13B; Fig. S27).

**Ocular segment and post-ocular segment 1–5** (presumably) forming distinct capsule (head capsule).

**Ocular segment and post-ocular segment 1–5** (presumably) forming distinct caspule (head capsule). Ocular segment recognizable by its appendage derivative, clypeo-labrum complex and pair of large compound eyes. Labrum oval, slightly invaginated, membranous. Clypeus continuous with labrum (Figs. 13A, 13B; Fig. S27). Frons (frontal sclerite) of post-ocular segment 1 with a pair of short setae, situated on top of small conical warts. Setae of frontal sclerite shorter than warts.

**Post-ocular segment 1** recognizable by its appendages, antennae [antennulae]. Antenna consisting of 16 elements. (Figs. 13A, 13B; Fig. S27). Antennae moderately long, following the dorso-posterior outlines of the compound eyes.

**Post-ocular segment 2** (intercalary segment) without externally recognizable structures.

**Post-ocular segment 3** without externally recognizable structures (mandibles).

**Post-ocular segment 4** recognizable by its appendage, maxilla [maxillula]. Maxilla organised into proximal part (non-serrated “lacinia”) and distal part, palp [endopod].

**Post-ocular segment 5** recognizable by its appendages, forming the labium [conjoined left and right maxillae]. Proximal part of labium membranous, bears labial palps (Figs. 13A, 13B; Fig. S27).

**Thorax** consists of three segments, pro-, meso- and metathorax. Each bears a pair of (ambulatory) appendages (fore, mid- and hind legs). Wings on mesothorax. Halterae on metathorax.

Thorax segments forming a single semiglobose structure, closely enveloping the head (Figs. 13A, 13B; Fig. S27).

**Ambulatory appendages** (legs) U-shaped folded, running between the wings; mid- and hind legs terminating anterior to the mid-length of the first posterior trunk (abdomen) unit. Ambulatory appendages do not curve between the wing tips, width of the legs stays constant, without divergence distally at the tips (Figs. 13A, 13B; Fig. S27). All ambulatory appendages subdivided into the elements: coxa, trochanter, femur, tibia and tarsus (subdivided into 5 elements).

**Prothorax** bears thoracic horns (modified spiracle 1). Thoracic horns club shaped, situated posterior to the eyes on the dorsal surface. Forelegs superimposed over the thorax appendages 2 and 3, not reaching wings tip. Forelegs with femur and tibia forming a U-shaped loop, with anteriormost point of the loop reaching the level at which maxillae arise.

**Mesothorax** bears a pair of wings. Antennae do not reach the base of the wing. Midlegs underlying the forelegs, reaching beyond the tip of the wing.

**Metathorax** bears a pair of halterae and a pair of spiracles. Hindlegs underlying the forelegs and midlegs, reaching beyond the tip of the wing (Figs. 13A, 13B; Fig. S27).

Length of head and thorax combined 1.9–2.2 mm (*n* = 2).

**Abdomen (posterior trunk).** With 9 units.

Abdominal units 1–8 each bearing two rings of strong hooklets. Four hooklets in the first ring, circa 48 hooklets in the second ring.

**Trunk end** (undifferentiated abdomen segments 9–11?) bears 6 hooklets, two at the anal lobes (Figs. 13A, 13B; Fig. S27). Abdomen 2.7–3.2 mm long (*n* = 2).

**Table:** 

**Figure 14:**
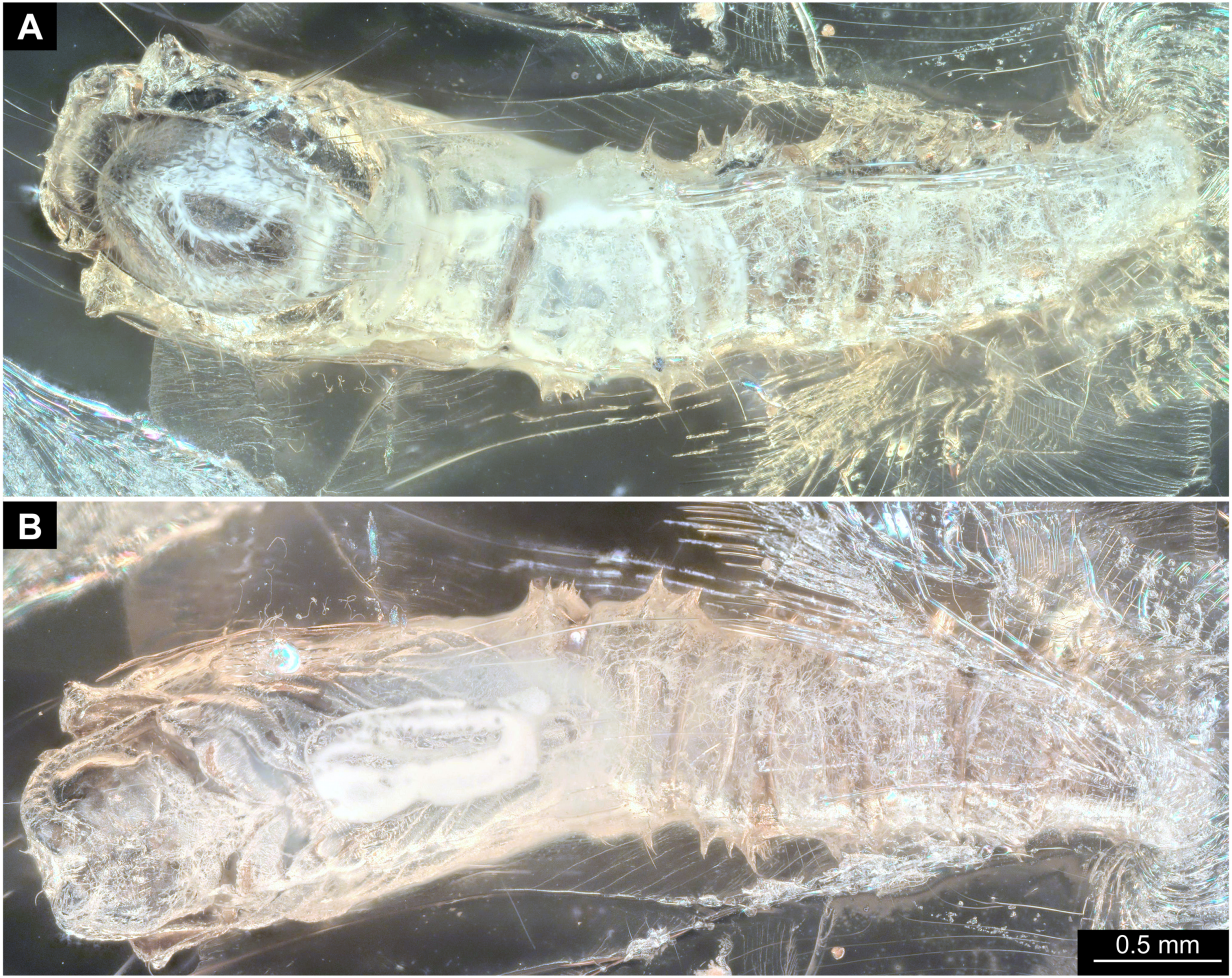
Fossil pupa, *Mycetobia* “morphotype 3”, pharate adult, DEI, collection number CCHH-DEI-608-2. (A) Habitus, dorsal view. (B) Habitus, ventral view.

*Mycetobia* pupae morphotype 3
(Figs. 14A, 14B; Figs. S28–S29)

*Material:* Morphotype 3 is represented by 2 specimens, one actual pupa and one adult emerging from exuvium: Table 1 and Figs. 14A, 14B; Figs. S28, S29.

Description:

**Habitus**. Medium-size insect pupae, with generally comma-shaped body. Pupae brown. Total length 0.82–0.86 mm long (*n* = 2). Body differentiated into presumably 20 segments, ocular segment plus 19 post-ocular segments (Figs. 14A, 14B; Figs. S28, S29). Anterior part of the body composed of head and thorax, visible as a single globose structure.

**Ocular segment and post-ocular segment 1–5** (presumably) forming distinct capsule (head capsule). Ocular segment and post-ocular segment 1–5 (presumably) forming a distinct capsule (head capsule). Ocular segment recognizable by its appendage derivative, clypeo-labrum complex and pair of large compound eyes. Labrum oval, slightly invaginated, membranous. Clypeus continuous with labrum (Figs. 14A, 14B. Frons (frontal sclerite) with a pair of short setae, situated on the top of small conical warts (Figs. 14A, 14B; Figs. S28, S29).

**Post-ocular segment 1** recognizable by its appendages, antennae [antennulae]. Antenna consisting of 16 elements. Antennae moderately long, following the dorso-posterior outlines of the compound eyes.

**Post-ocular segment 2** (intercalary segment) without externally recognizable structures.

**Post-ocular segment 3** without externally recognizable structures (mandibles) (Figs. 14A, 14B; Figs. S28, S29).

**Post-ocular segment 4** recognizable by its appendage, maxilla [maxillula]. Maxilla with proximal part (non-serrated “lacinia”) and distal part, palp [endopod] (Figs. 14A, 14B; Figs. S28, S29).

**Post-ocular segment 5** recognizable by its appendages, forming the labium [conjoined left and right maxillae]. Proximal part of labium membranous, bears labial palps (Figs. 14A, 14B; Figs. S28, S29).

**Thorax** consists of three segments, pro-, meso- and metathorax. Each bears a pair of (ambulatory) appendages (fore, mid- and hindlegs). Wings on mesothorax. Halterae on metathorax.

Thorax segments forming a single semiglobose structure, closely enveloping the head of the pupa.

**Ambulatory appendages** U-shaped folded, running between the wings; mid- and hind legs terminating above the mid-length of the first posterior trunk (abdomen) unit. Ambulatory appendages curving between the wing tips, and then, diverging again after passing the tips of the wings (Figs. 14A, 14B; Figs. S28, S29). All ambulatory appendages subdivided into elements:: coxa, trochanter, femur, tibia and tarsus (subdivided into 5 elements).

**Prothorax** bears thoracic horns (modified spiracle 1). Thoracic horns club shaped, situated posterior to the eyes on the dorsal surface of the prothorax. Prothorax bears first thoracic appendage pair (forelegs). Forelegs superimposed over the thorax appendages 2 and 3, not reaching wings tip. Forelegs with femur and tibia forming a U-shaped loop, with anteriormost point of the loop reaching the level at which maxillae arise (Figs. 14A, 14B; Figs. S28, S29).

**Mesothorax** bears a pair of wings. Midlegs underlying the forelegs, reaching beyond the tip of the wing (Figs. 14A, 14B; Figs. S28, S29). Base of the wing aligned with the tip of the antennae.

**Metathorax** bears a pair of halterae and a pair of spiracles. Hindlegs underlying the forelegs and midlegs, reaching beyond the tip of the wing (Figs. 14A, 14B; Figs. S28, S29). Base of the wing aligned with the tip of the antennae.

**Abdomen (posterior trunk)** with 9 units.

**Abdominal units 1–8** each bearing two rings of strong hooklets.12 hooklets in the first ring, circa 70 hooklets in the second ring.

**Trunk end** (undifferentiated abdomen segments 9–11?) bears a pair of the lateral expansions (anal lobes) and 8+2 hooklets. Hooklets arranged in 2 rings, two additional hooklets sitting on anal lobes (Figs. 14A, 14B, Figs. S28, S29). Abdomen length 0.5–0.6 mm (*n* = 2).

**Figure 15:**
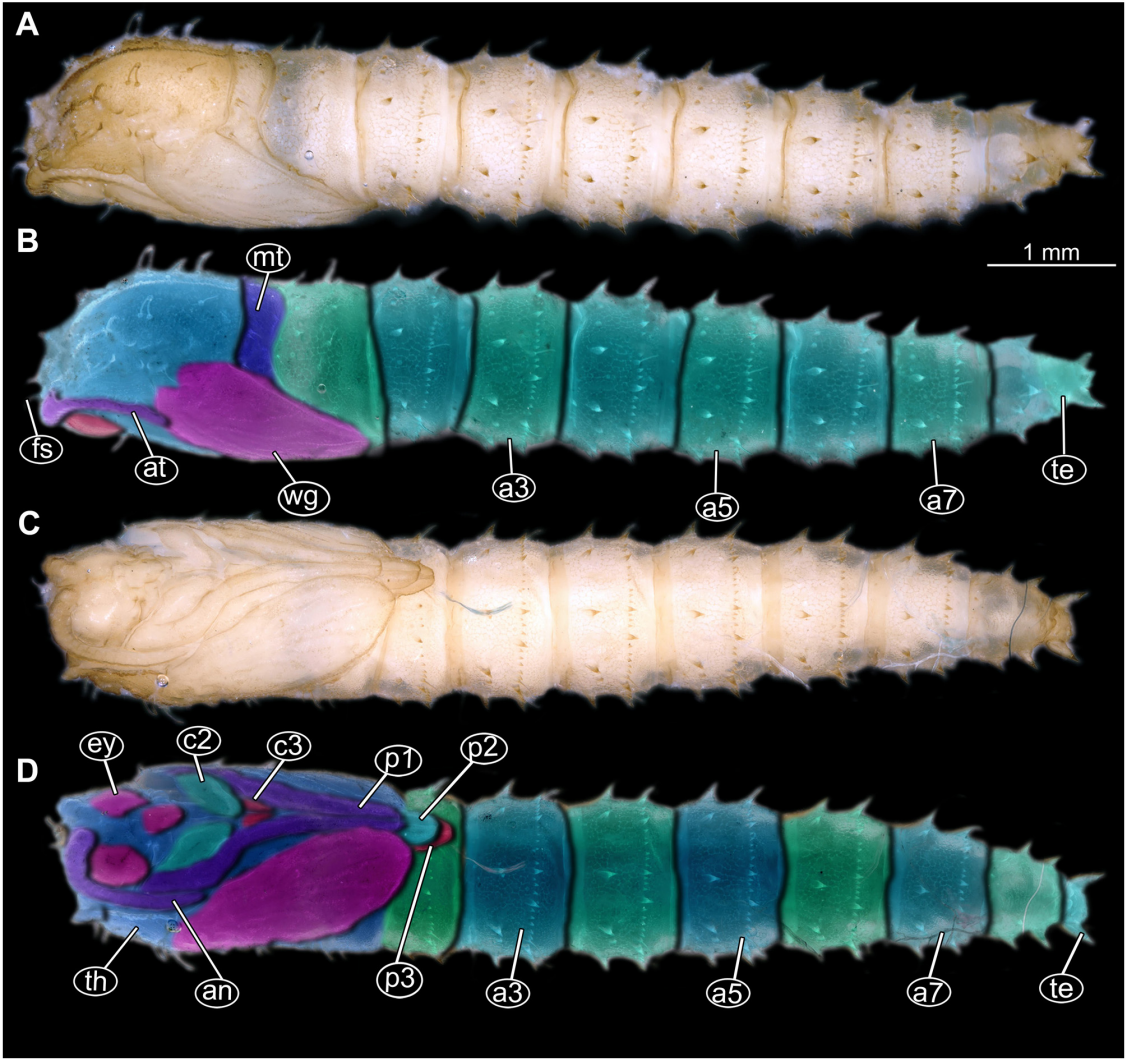
Extant pupa, *Mycetobia pallipes* (Meigen, 1818), ZSM, no collection number assigned. (A) Habitus, dorsal view. (B) Colored version of A. (C) Habitus, ventral view. (D) Colored version of C. Abbreviations: an-antennae; a3–a7, abdominal segments 3–7: ey, eyes; fs, frontal setae; mt, methathorax; p1, front legs; p2, midlegs; p3, hind legs; te, trunk-end; th, thoracic horn; wn, wing.

*Systematic interpretation* (all 3 morphotypes):

Pupae of all three morphotypes possess a single pair of wings on the mesothorax and developing halterae on the metathorax identifying them as pupae of the group Diptera. They are interpreted as representatives of Anisopodidae based on the following combination of characters: slender; antennae long, reaching, at least, until to the wing base; forelegs not reaching tip of wing, but mid and hindlegs reaching beyond the wings; thoracic horns small and oval to mushroom-like; spiracles present on metathorax and abdominal units 2–7. Last unit of abdomen bearing four pairs of strong denticles (Figs. 15A–15D).

Pupae of all three morphotypes possess characters autapomorphic for the group *Mycetobia* (ingroup of Anisopodidae): head bearing short frontal setae on conical warts; anterior and posterior margins of abdominal tergites bear rows of strong denticles.

Pupa morphotypes 1 and 2 can be distinguished from each other based on the number of denticles in the anterior row of the tergites, four in morphotype 2 and twelve in morphotype 1. Morphotype 1 can potentially include numerous species, indistinguishable in this stage and especially degree of preservation. Another diagnostic character differentiating the two morphotypes is the presence of a distal outward curvature of the legs of the morphotype 1, while morphotype 2 legs are of the constant width. Morphotype 3 is highly reminiscent of morphotype 1 but is significantly smaller, only about 30% of the total length of morphotype 1.

It is worth mentioning that the morphotypes might in fact result from sexual dimorphism. Yet, the examination of pupae of the extant species *Mycetobia pallipes* did not show any notable sexual dimorphism among the examined (non-pharrate) pupae, also not concerning size. However, it will require examination of many more species of *Mycetobia* to draw any well-founded conclusions.

*Taxonomic attribution:* The morphology of both the larvae and the pupae is entirely in line with corresponding stages of extant representatives of *Mycetobia.* At least some of the representatives of pupa morphotype 1 are most likely representatives of *Mycetobia connexa*, which is the most abundant species of *Mycetobia* in Baltic amber (Wojton, Kania & Krzeminski, 2019). This is indicated by the common preservation in the amber piece PED-4395, which contains a single exuvium of a pupa of morphotype 1 as well as two adult representatives of Anisopodidae, a male and female (Figs. S11, S15). This male is a representative of *Mycetobia*, based on the following combination of characters: wing without discal cell, medial vein with three branches , radial vein 2+3 ending in costa, radial vein 4+5 ending proximal to the end of the costal vein, anal vein 1 very faint (Hancock, 2017). It can be interpreted as a representative of *Mycetobia connexa* Meunier, 1899 based on the following combination of characters: antenna elements (flagellomeres) 8–13 up to two times as long as wide; distal element of maxillary palp (palpomere) at most 3 times as long as wide, thinned; subcostal vein ending proximal to radial sector bifurcation; radial vein 1 ending on costal vein apex proximally of medial vein 1+2 bifurcation; fork of medial vein 1+2 wide; medial vein 1+2 elongated, as long as medial vein 1; medial vein 2 and medial vein 3+4 separated by a distance at least two times as the distance between ends of the medial vein 1 and medial vein 2; radial vein 2+3 two and 50% as long as radial sector or shorter; tarsus of foreleg 30% of the length of entire leg (including the coxa; Figs. S11, S15) (Wojton, Kania & Krzeminski, 2019). We interpret the male and the female of the *Mycetobia* inclusions in this piece as both being representatives of *M. connexa* based on the identical wing venation and similar antennae. We have associated the pupal exuvium with the adults, based on their proximity in amber (Figs. S11, S15).

It is so far impossible to determine associations of the studied larvae with any of the seven species of *Mycetobia* currently known from Eocene European ambers (Wojton, Kania & Krzeminski, 2019). Future records of pupal exuvia with emerging or pharate adults and/or associated larval exuvia may allow for the association of further life stages. The record of three pupal morphotypes of *Mycetobia* in Baltic and Bitterfield amber is unsurprising, given the relatively high species richness of *Mycetobia* in those Lagerstätten (Wojton, Kania & Krzeminski, 2019).

**Table:** 

**Figure 16:**
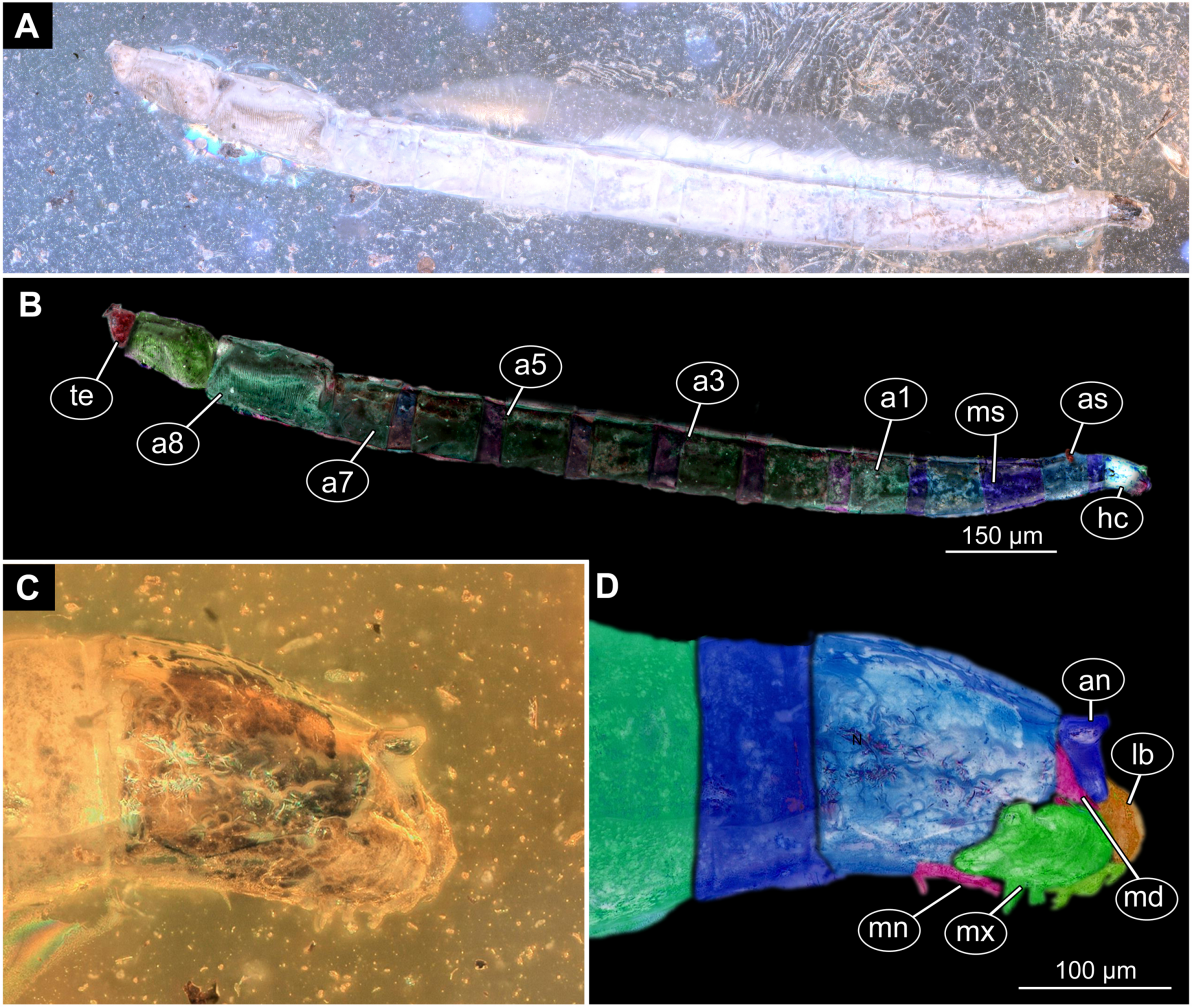
Fossil larva, *Sylvicola*, DEI, collection number Dip-00642. (A) Habitus, lateral view. (B) Colored version of A. (C) Head capsule, lateral view. (D) Colored version of C. Abbreviations: a1–a8, abdominal segments 1–8; an, antennae; as, anterior spiracle; hc, head capsule; lb, labrum; md, mandible, mn, mentum; mx, maxilla; ms, mesothorax; te, trunk end.

Anisopodidae Knab, 1912
*Sylvicola* Fatio, 1867
(Figs. 16A–16D)

*Material:* Single larva, in Baltic amber, DEI Dip-00641.

Description:

Habitus. Medium sized larva with roughly vermiform body. Total length 6.4 mm. Body differentiated into presumably 20 segments, ocular segment plus 19 post-ocular segments (Figs. 16A–16D).

**Head**. Ocular segment and post-ocular segment 1–5 (presumably) forming distinct caspule (head capsule). Head capsule longer than wide. Head capsule well developed, fully sclerotized dorsally, partially sclerotized ventrally. Head capsule in dorsal view not accessible due to orientation of the specimen. Hind part of head capsule not retracted into anterior trunk. Head capsule 280 µm long. Surface of head capsule smooth and glossy (Figs. 16A–16D).

**Ocular segment** without apparent stemmata (larval eyes). Ocular segment recognizable by its appendage derivative, clypeo-labrum complex. Labrum 70 µm long (Figs. 16A–16D).

**Post-ocular segment 1** recognizable by its appendages, antennae [antennulae]. Antenna conical, consisting of one element, 44 µm long.

**Post-ocular segment 2** (intercalary segment) without externally recognizable structures.

**Post-ocular segment 3** recognizable by its pair of appendages, mandibles. Mandible only accessible at the distal tip, proximal part obscured. Mandible divided into large, unsclerotised proximal portion, and heavily sclerotized distal portion, bearing numerous teeth.

**Post-ocular segment 4** recognizable by its appendage, maxilla [maxillula]. Maxilla massive, organised into proximal part and distal part, palp [endopod]. Proximal part of the maxilla fleshy, very weakly sclerotized, only general outline visible. Maxilla bears six cone-like outgrows, probably sensillae. Proximal part differentiated into two lobes, outer lobe and inner lobe (Figs. 16A–16D).

**Post-ocular segment 5** recognizable by its appendages, forming the labium [conjoined left and right maxillae].

**Trunk** composed of 11 visible units: pro-, meso- and metathorax plus 8 abdominal units. Trunk worm-like, units sub-equal in diameter. Trunk lacks parapodia and/or creeping welts. Trunk bears two pars of spiracles, on prothorax and abdominal unit 8.

**Thorax** consists of three segments, pro-, meso- and metathorax.

**Prothorax** bears small, cone-shaped, anterior spiracles situated on postero-latero-dorsal surface. Prothorax subdivided into two unequal parts by annular constriction.

**Meso**-and **Metathorax** subequal to prothorax, but without spiracles.

**Abdomen (posterior trunk)**. Abdominal units are cylindrical, roughly equal to each other in diameter (Figs. 16A–16D).

**Abdominal units 1–7** subdivided into two unequal parts by annular constriction.

**Trunk end** (undifferentiated abdomen segments 8–11?) subdivided into three unequal parts by two annular constrictions. Trunk end covered with perianal shield (modified area of the last unit surrounding the anal aperture) on the ventral side. Trunk end bears posterior spiracles situated on the medio-postero-dorsal surface of the unit. Spiracular field surrounded by five triangular, setose lobes.

*Systematic interpretation*: The general body shape, as well as absence of the ambulatory legs on the thorax, and the spiracle arrangement is consistent with this larva being an immature stage of the group Diptera. Numerous characters indicate that the specimen is a larva of the group Anisopodidae: body slender, vermiform; head fully sclerotized, dorsal part more strongly sclerotized than ventral; mandible with fleshy proximal heavily sclerotized distal part; prothorax and abdominal segments 1–7 subdivided into the two unequal parts by an annular constriction; respiratory system amphipneustic; anterior spiracle forming small cone on prothorax; posterior spiracles on spiracular field, on the posterior end; trunk end with perianal shield; the trunk end subdivided into three parts.

The fossil larva possesses a spiracular disc surrounded by triangular setose lobes. The character is autapomorphic for the group *Sylvicola* (ingroup of Anisopodidae). In larvae of other ingroups of Anisopodidae the spiracle is surrounded by roundish lobes, bare of setae. The structure of the spiracular disc can be used to distinguish between larvae of *Mycetobia* and *Sylvicola* (Hancock, 2017) also in fossilized resin.

The morphology of the fossil (Dip-00642) resembles extant larvae of *Sylvicola* to a high degree (cf. Keilin & Tate, 1940). Due to the preservation of the specimen, no characters could be observed to reliably differentiate between the fossil larva from larvae of the extant species *Sylvicola fenestralis* (Scopoli, 1763). It is also impossible to identify the larvae as a representative of any of the five known species of *Sylvicola* from Baltic amber, as all of them are known from adults only (Wojtoń, Kania & Kopeć, 2018).

*Syninclusions:* stellate hairs and plant detritus are preserved in the same amber piece as the studied specimen.

## Discussion

### Species diversity and morphological diversity

Our investigations of Baltic and Bitterfeld amber material yielded at least four larval and three pupal morphotypes of Bibionomorpha. One larval type is even known from several instars.

There are probably numerous species of *Mycetobia* represented among the larval specimens. Yet, due to the degree of preservation it is impossible to distinguish them. The presence of several species within the material appears to be almost a certainty, taking into account the species diversity of Bibionomorpha in Baltic and Bitterfeld amber represented by adult forms, including at least 12 species of Anisopodidae (Wojtoń, Kania & Kopeć, 2018; Wojton, Kania & Krzeminski, 2019; Wojton et al., 2019). Also, other bibionomorphan lineages show a quite rich fossil record in these amber Lagerstätten, again represented by adults, with at least 3 species of Hesperinidae, 10 species of Bibionidae and numerous species of the group Sciaroidea (Skartveit, 2002; Skartveit, 2009).

It is indeed surprising that the apparently abundant material of larvae and pupae of Bibionomorphan lineages in Eocene European amber has not attracted the attention of the scientific community earlier. There were some brief reports of pupae of Anisopodidae and Cecidomyiidae (Weitschat, Berning & Podenas, 2009), but also these did not seem to attract much further attention. In a study by Haug et al. (2017), dealing with a group of dipteran pupae in a single amber piece, four specimens apparently representing morphotype 2 of *Mycetobia* have been reported (Haug et al., 2017), yet misidentified as pupae of Asilidae, due to the somewhat similar structure of the spines or denticles on the trunk. Other pupae of Anisopodidae, without specification of further reaching taxonomic details have been reported from Miocene Dominican amber (Grimaldi, 1991).

No further immature stages of bibionomorphans have been reported from amber so far (Skartveit, 2017). This is probably a reflection of the fact, that in palaeoentomology, immature stages of the group Insecta often seem to be considered as “inferior material” in comparison to adults. A possible reason for that is the relative difficulty of relating of taxa described based on larvae and pupae to the other taxa, which have been described based on adults. This might act as disincentive in a field, where *α*-taxonomy is still seen as a pinnacle of research achievement (Azar et al., 2018).

Still, taking in account the seeming general scarcity of larval forms of Diptera preserved in amber (Andersen et al., 2015; Baranov et al., 2019a; Baranov et al., 2019b), the high abundance of larvae of Bibionomorpha in Eocene European ambers is remarkable. The taphonomic window of the fossilized resins seems strongly biased towards flying, hence adult representatives of Insecta (or better Pterygota), especially for adult forms of Diptera (Solórzano-Kraemer et al., 2015). Larvae of Diptera often live in aquatic habitats, soil, leaf litter or are internal parasites of plants and animals and thus have limited opportunities for entrapment in plant resins and the subsequent preservation as amber inclusions (Solórzano Kraemer et al., 2018; Kirk-Spriggs & Sinclair, 2017).

Perkovsky et al. (2012) have shown that there is a stable structural cohort of animals preserved in Baltic and Rovno amber, which they termed “Sciara-zone Diptera”, which made up to 20% of all inclusion in representative batches of Baltic and Rovno amber. “Sciara-zone Diptera” is represented mostly by flies of the groups Bibionomorpha and Tipulomorpha, possessing xylophagous or saprophagous larvae, which apparently were associated with the tree-trunks in the Baltic amber forest (Perkovsky et al., 2012). Larval forms of “Sciara-zone Diptera”, and especially those of Anisopodidae, are also living on tree trunks or right beneath them in the upper leaf-litter. This makes their preservation in fact highly likely in comparison to other larval forms of Diptera (Hancock, 2017). The preservation of a large number of immature of *Mycetobia* is in line with recent research on the entrapment bias in amber. This research (Sánchez-García et al., 2017; Solórzano Kraemer et al., 2018) has shown that the taphonomic window of amber deposits is positively selecting towards fauna associated with tree trunks, while negatively selecting against species from the certain other habitats, i.e., hygropetric water films (aquatic habitats formed by the thin layers of water seepagin from the soil) and true aquatic habitats (Sánchez-García et al., 2017).

Such a high abundance of larvae and pupae of Bibionomorpha provides an unprecedented look at the role of immature stages in the European Eocene amber forest. Since most of the immature stages of the Bibionomorpha in the studied material are closely reminiscent of corresponding stages of extant species, we can extrapolate the ecology of the fossil larval forms of Bibionomorpha to have been similar to their extant relatives (Wichard, Gröhn & Seredszus, 2009).

In fact, we have not been able to discern any substantial difference between studied larvae of *Mycetobia*, *Sylvicola* and *Pachyneura* preserved in amber and their extant counterparts. This is partially caused by the relatively low “resolution” of the characters in the fossil material, which does not allow to recognise more subtle differences between fossil larvae and their extant relatives.

Extant larvae of Pachyneuridae are associated with dead wood in pristine forests (Paramonov & Salmela, 2015). We assume a similar life habit for the fossils.

Extant larval representatives of *Mycetobia* and *Sylvicola* are associated with decaying organic material, mostly plant tissue. Yet, dung or animal corpses might also be occasionally exploited (Hancock, 2017). We can therefore assume that abundant larvae of *Mycetobia* (but also the larva of *Sylvicola*) preserved in Eocene amber were originally likewise connected to decaying organic matter. It is quite conceivable that a subtropical, seasonal forest in the Eocene of Europe would yield plenty of decaying organic matter, in the form of leaf litter, dead plant or animal matter, bacterial biofilms and fungi (Hancock, 2017; Wojton et al., 2019).

### Ontogeny of the fossil forms of Mycetobia

**Figure 17:**
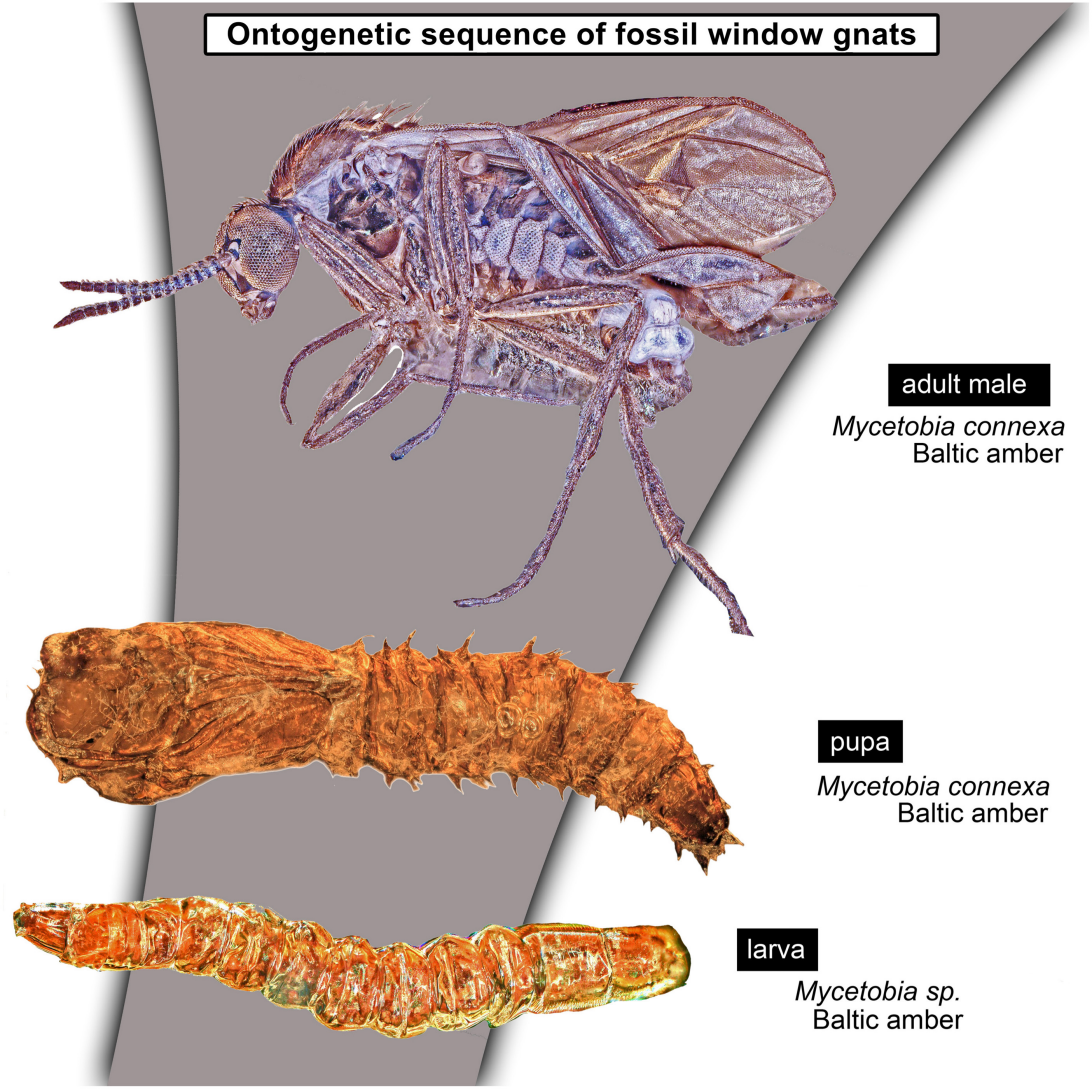
Reconstructed ontogenetic sequence for representatives of *Mycetobia* in the Eocene.

The relatively large amount of immature (“preimaginal”) specimens of the species group (“genus”) *Mycetobia*, allows to do a limited quantitative analysis of the post-embryonic ontogeny of these flies (Fig. 17). Coombs, Cleworth & Davies (1997) have shown that representatives of Anisopodidae have four larval stages in their development. This was not based on rearing larvae in the lab, but rather on looking at the distribution of several morphometric parameters. Head capsule length, head capsule width and body length have been measured for 303 larvae of *Sylvicola fenestralis* (Scopoli, 1763). Coombs, Cleworth & Davies (1997) found that at least the head capsule width distribution followed a distinct four-peak pattern, corresponding to four supposed larval stages for this species.

‘Dyar’s rule’, describes the pattern of larval development in Holometobola (Dyar, 1890). In particular, it describes the inter-moult growth within Holometabola occurring at a similar rate for each larval stage. As a short remark: this pattern is even more general and not only true for Holometabola, but also for other crustaceans (cf. ‘Brook’s law’, e.g., Fowler, 1909). This strict pattern can be used to infer the number of larval stages from the available dataset on larval morphometry (Coombs, Cleworth & Davies, 1997). In particular, mean values for every size cohort of log-transformed datasets should follow a straight line, with high values of R^2^. If the mean values behave differently, deviating from a straight line, this would mean a larval stage (size cohort) missing from the plot (Dyar, 1890; Coombs, Cleworth & Davies, 1997). Coombs, Cleworth & Davies (1997) have shown that the factor, with that the head capsule width increases between the larval stages of *Sylvicola fenestralis*, remains relatively constant (0.57–0.66) and follows Dyar’s rule (Dyar, 1890; Coombs, Cleworth & Davies, 1997).

**Figure 18:**
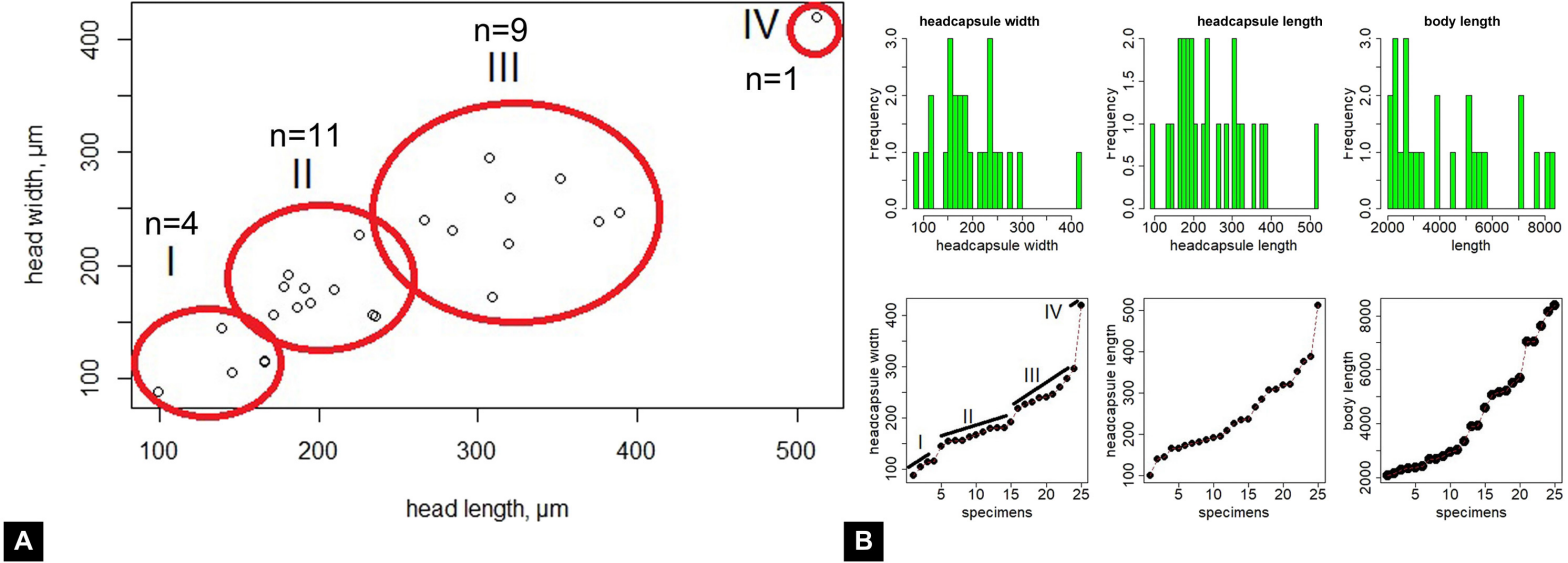
Summary statistics. (A) Biplot of fossil larvae of *Mycetobia* (*n* = 36), head capsule length vs. head capsule width, red circles indicate hypothetical divisions into different larval stages based on the gaps in the data point distribution. I–IV, number of hypothetical larval stages. (B) Distribution of the size cohorts within a sample of the fossil larvae of* Mycetobia*; upper-row-left, histogram of the head capsule width distribution (*n* = 26); upper-row-center, histogram of the head capsule length distribution (*n* = 25); upper-row-right, histogram of the body length distribution (*n* = 36); lower-row-left, ranged plot (values ordered in ascending order) of the head capsule width, hypothetical division into different larval stages based on gaps in data point distribution indicated with I–IV as numbers of supposed larval stages; lower-row-centered, ranged plot (values ordered in ascending order) of head capsule length; lower-row-right, ranged plot (values ordered in ascending order) of body length.

**Figure 19:**
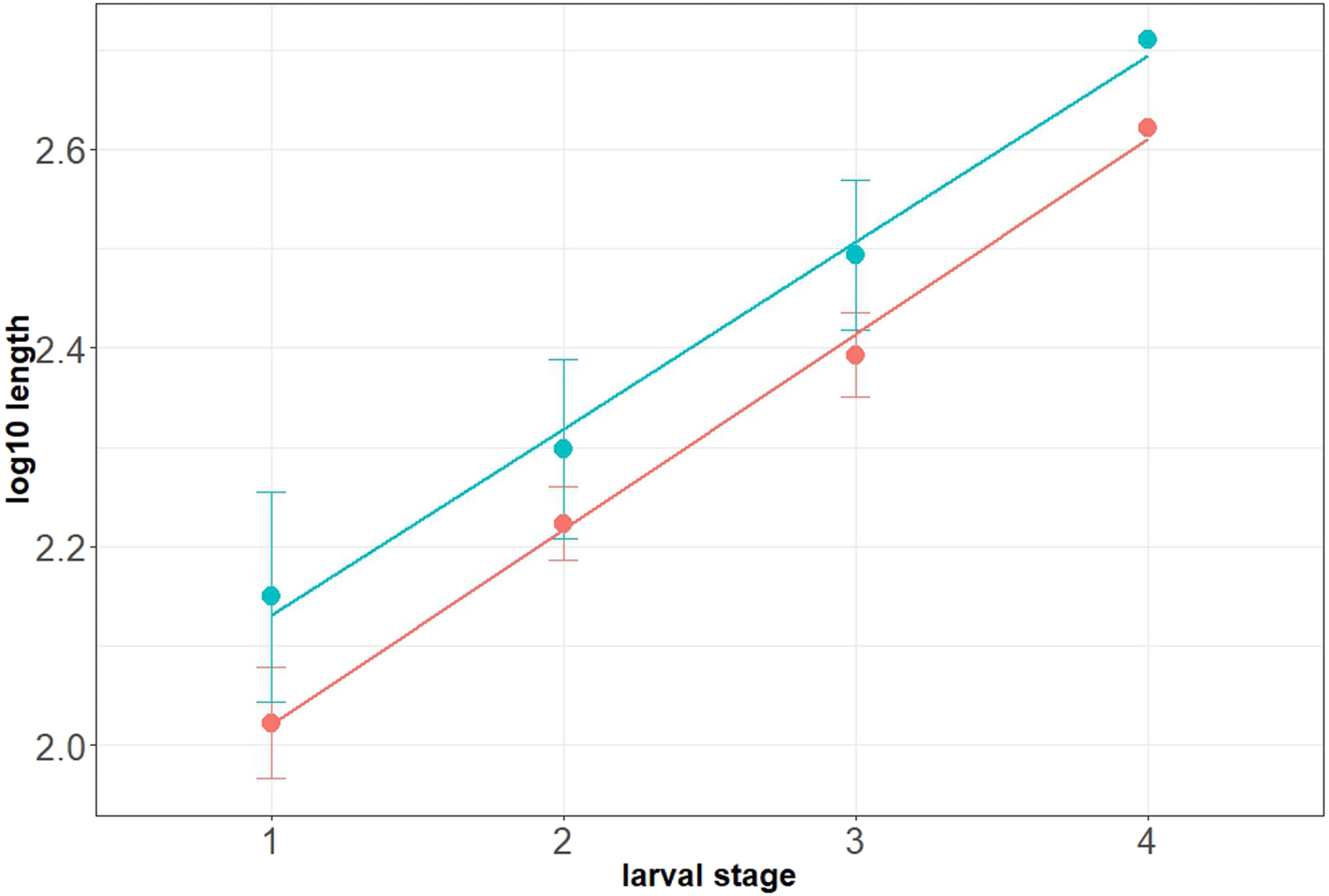
Natural logarithm of the mean larval head capsule width (red) and head capsule length (blue) of fossil larvae of *Mycetobia*, plotted against associated instar number. The fourth larval stage is represented by a single specimen, therefore the actual values are plotted instead of the mean. Red dots and the line representing the head capsule width, while blue represents the head capsule length. Error bars are representative of the value’s standard deviation.

We applied the approach of Coombs, Cleworth & Davies (1997) to our material and found that values of the head width and the head length of the fossil plotted in increasing order as shown in Figs. 18A and 18B are falling into four discrete categories (Figs. 19A, 19B). The line charted through the ordered dot-plot has 3 clear breaks for both the head length and the width of the head, but not for the body length (Fig. 19). This indicates the presence of four larval stages (based on head capsule width). We think that the absence of such breaks in the body length plot, is connected to the taphonomic conditions of the larvae. It is possible that, upon the entrapment in amber, the larvae would shrink, obscuring the reconstruction of the original body length. In fact, McCoy, Soriano & Gabbott (2018) have shown by actuo-taphonomic experiments that the specific type of the fossil resin, desiccation prior to entombment and the composition of the gut microbiota all have a crucial impact on the preservation-quality of fossil insects. They have shown that the combination of the above mentioned factors will determine whether specimens will be preserved with soft tissue, as cuticular fossil only, or not at all (McCoy et al., 2019). Therefore, significant preservation biases can occur based on the identity of the insect and amber deposit. Therefore, it is even more advisable to use only hard-sclerotized structures (such as head capsule), which are less prone to be deformed, for morphometrical purposes.

We proceeded to calculate the mean value of the head width and length for each of the cohorts observed in the plot. Then, those mean values were plotted against the supposed larval stage. Dyar (1890) and Coombs, Cleworth & Davies (1997) have shown that if the values of morphometric parameters plotted against the supposed number of the larval stages are following a linear trend, that means that the studied sample contains all larval stages of the studied species (Fig. 17).

In our case, we have separated the stages based on the width of the head capsule, as Coombs, Cleworth & Davies (1997) have shown it to be the most reliable predictor of the life-stage distribution in the measured larvae (Figs. 17, 18B). In our data the average values for both the head width and the head length follow a perfect linearly increasing trend. The R^2^ value for the head-width trend was 0.98 for the head width and 0.99 for the head length (Fig. 19).

Our data therefore supports the presence of four larval stages in the larval development of the Eocene *Mycetobia* species. The factor of growth between the stages is relatively steady, namely 0.6, and is consistent with Dyar’s rule (Coombs, Cleworth & Davies, 1997; Table 2).

This is the first time that a full ontogenetic post-embryonic series of a dipteran could be reconstructed based on amber material. A more incomplete series of single larval stage, pupa and adult has been presented by Baranov et al. (2019b). The reconstructed ontogeny of *Mycetobia* from amber demonstrates that during the Eocene Anisopodidae had lineages with representatives exhibiting derived morphologies and an ontogenetic development which is indistinguishable from extant forms of Anisopodidae (Wojton et al., 2019).

### Larvae of Bibionomorpha and amber forest ecology

Within the scientific community, a new understanding of the European Eocene amber forest (Seyfullah et al., 2018; Schmidt et al., 2019), as a warm-temperate seasonal forest, is currently emerging. This reconstruction is based on contemporary studies of palaeobotanical species complexes, fungi and microorganisms as well as isotope signatures, preserved in these ambers (Seyfullah et al., 2018; Schmidt et al., 2019). This reconstruction has currently not yet triggered a re-interpretation of insect communities in these ambers, however it will likely cause such a reinterpretation in the future.

The major weakness of the current interpretation of the palaeoecology of Insecta in Eocene amber, is that it is based on a very coarse application of the uniformitarism principle to the ecology of now extinct groups (Grund, 2006; Seredszus & Wichard, 2011; Zelentsov et al., 2012; Baranov, Andersen & Perkovsky, 2015). This means there is a mechanistic phylogenetic inference, in which fossil representatives of species groups (“genera”) are automatically assumed to have the autecological traits of the seemingly closest modern relatives. Yet, this is a mere oversimplification and likely malicious for the results and conclusions of such studies (Grund, 2006). Many authors, have shown that in case of large and ecologically “diverse genera”, or “relic genera” (groups which which were much more diverse in the past), such inferences might lead to the widely inaccurate conclusions (e.g., Stebner et al., 2017; Baranov et al., 2019a; Baranov et al., 2019b). This problem is of course also a result of the (unreflected) use of taxonomical ranks, as a low ranks (such as the genus) appear to suggest a close relationship among the included species. However, the assignment of ranks is a completely arbitrary decision (Mayr, 1942) and neither consistently reflects the age of a group nor the relatedness among species belonging to this group and as much less in a way that this would be comparable on a larger systematic scale (Ereshefsky, 2002; Dubois, 2007).

It is worth noting in this aspect, that the paleoecology of many fossil species with aquatic larvae such as non-biting midges (Diptera, Chironomidae) or caddisflies (Trichoptera) is interpreted based on the larval ecology of their extant relatives, yet inferred by fossils of the adults (for examples see (Wichard, Gröhn & Seredszus, 2009)). It is done in this way, as these groups of Insecta are widely used in aquatic biomonitoring today, and their larval habitats are thought to be rather narrow and well known (Merritt & Cummins, 1996).

The weakness of this approach for palaeohabitat reconstructions, is that it represents a type of double-inference, in case it is based on adults. (1) One infers a close relationship between the fossil (adult) animal and its extant relatives, for the larval ecology is known. (2) One assumes that the larvae of the fossil adult animal behaved similar to their extant counterparts, without access to the larval morphology (Wichard, Gröhn & Seredszus, 2009).

A more direct interpretation of the ecology of larvae, which are more tied to particular habitats (in many lineages of Insecta larvae perform most of the ecological functions) is considered advantageous in comparison to the above mentioned double-inference. Such an advantage arises from the direct observation of the larval morphology, which in combination with the interpretation of the taphonomic situation and the possible presence of syninclusions can tell a lot about the ecology of an animal (Andersen et al., 2015; Baranov et al., 2019b).

Hence, the observed details of immature forms of Bibionomorpha eliminate one level of assumptions and provide more direct indications of the palaeohabitat. The high abundance of immatures of Anisopodidae in Eocene European amber forests, may indicate moist conditions and a large amount of decaying organic matter on the forest floor, a habitat characteristic for extant representatives of Anisopodidae (Hancock, 2017). This is reaffirming similar conclusions made based on the abundant co-occurence of non-biting midges (Diptera, Chironomidae) with terrestrial larvae in Baltic amber (Andersen et al., 2015; Baranov et al., 2019a; Baranov et al., 2019b). Secondly, the presence of a larva of Pachyneuridae (xylobiont-xylophages, living in the deep layer of xylem of old, still living trees) is indicative for pristine temperate forests in extant conditions (Krivosheina, 2006; Paramonov & Salmela, 2016). Therefore, in the Eocene it might translate to mature forest communities with large quantities of the dead wood. Hence, the findings of larval forms of Diptera provide a new independent source of information that can be used for palaeohabitat reconstruction.

## Conclusions

This first examination of immatures of Bibionomorpha from Baltic and Bitterfeld amber is based on more than 60 specimens, representing three major ingroups of Bibionomorpha: Bibionidae (or a possible sister species to it), Pachyneuridae and Anisopodidae. Bibionidae (or its sister species) and Pachyneuridae are both represented by a single larval morphotype; Anisopodidae is represented by at least two larval morphotypes and at least three pupal morphotypes.

The larva of Pachyneura is the first fossil record for this group. The presence of this larva, indicates pristine, temperate forest conditions, with abundant old trees. This lines up well with the emerging new interpretation of the Baltic amber forest as a warm-temperate, seasonal ecosystem (Schmidt et al., 2019).

Window gnats (Diptera, Anisopodidae), are the most abundant immature stages of bibionomorphans in Bitterfeld and Baltic amber. A large number of fossil immatures allowed us to reconstruct the full post-embryonic ontogenetic series of fossil representatives of *Mycetobia* (Anisopodidae). This reconstruction is only the second one for dipterans in amber (first in Baranov et al., 2019b), and also the most complete. It demonstrates that in the Eocene representatives of *Mycetobia*, just as their extant counterparts, had four larval stages.

This study shows the large potential of future studies on fossil larvae of flies in amber. Contrary to the widespread opinion, these larvae are relatively abundant. Their abundance, and ecological information associated with them (plus the additional information from syninclusions and other clues about the taphonomy), might be crucial to further elucidate the new, emerging picture of the palaeoecosystems that are preserved by Baltic and Bitterfeld amber.

##  Supplemental Information

10.7717/peerj.7843/supp-1 Figure S1 Fossil larva, holotype of *Dinobibio hoffeinseorum* sp.n. GPIH, accession number (GPIH-0024) (A) ventro-lateral view. (B) dorso-lateral view; (C1–C2) spiracle 10. (D1–D2) spiracle 2. (E1–E2) spiracle 1.

10.7717/peerj.7843/supp-2 Figure S2 Fossil larvae, *Mycetobia* with syninclusions, GPIH, collection number GPIH-0247 (A) overview of the amber piece. (B) caddisfly male, Polycentropodidae. (C) partial syninclusion of an adult beetle. 1–4, larvae of *Mycetobia*; 5, beetle; 6–10 larvae of *Mycetobia*; 11, caddisfly male, Polycentropodidae

10.7717/peerj.7843/supp-3 Figure S3 Fossil larvae, *Mycetobia* with syninclusions, collection number Dip-00640 (A) Overview of the inclusions. (B–D) dipterans, non-biting midges (Chironomidae)*.* (B) *Rheosmittia pertenuis*,** male. (C) Orthocladiinae, female. (D) *Rheosmittia pertenuis*,** male, second specimen*.* (E) partial inclusions of *Mycetobia* sp. larvae. 1–4 Mycetobia larvae; 5–6 *R. pertenuis*, males; 7 Orthocladiinae, female.

10.7717/peerj.7843/supp-4 Figure S4 Fossil larvae, *Mycetobia*, DEI, collection number Dip-00639 (A) habitus. (B) trunk end, with posterior spiracles. (C) head capsule, ventral view.

10.7717/peerj.7843/supp-5 Figure S5 Fossil larvae, *Mycetobia* (A) PED-5695. (B) DEI, collection number Dip-00654. (C) GPIH (BI-2350). (D) PED-4965.

10.7717/peerj.7843/supp-6 Figure S6 Fossil larva, *Mycetobia* with syninclusions, collection of GPIH, collection number 3706-W (A) mite. (B) fly, Phroidae. (C, D) larval specimen of *Mycetobia*. (C) ventral view. (D) dorsal view.

10.7717/peerj.7843/supp-7 Figure S7 Fossil larvae, *Mycetobia* A) Two specimens, GPIH (L-7592). (B) two specimens, GPIH (L-7592). (C) four specimens (1–4), PED, collection number PED-4748. (D) larva with syninclusions, PED, collection number PED-4970. 1, scale insect, (Coccoidea), nymph; 2, leaf hopper (Cicadellidae), nymph; 3, larva, *Mycetobia*; 4, non-biting midge (Chironomidae), female.

10.7717/peerj.7843/supp-8 Figure S8 Fossil larvae, *Mycetobia*, DEI, collection number Dip-00649 (A) large larva. (B) specimens 1–3. (C) large larva.

10.7717/peerj.7843/supp-9 Figure S9 Fossil larvae, *Mycetobia* with syninclusions A) Overview of the amber piece Dip-00656from the collection of DEI. (B–D) larvae, *Mycetobia. (B) specimen 1. (C) specimen 2. (D) specimen 3.* 1, 2, 5, larva, *Mycetobia*; 3, 8, 10, 14 gall midges (Cecidomyiidae); 4, mite (Acari); 6, fly (“Acalyptrata”); 7, beetle (Coleoptera); 9, 11–13, ants (Fromicidae).

10.7717/peerj.7843/supp-10 Figure S10 Fossil larvae, *Mycetobia*, DEI, collection number Dip-00655 (A) specimen 1. (B) specimen 2.

10.7717/peerj.7843/supp-11 Figure S11 Fossil pupa (exuvium), *Mycetobia* “morphotype 1” with syninclusions, collection number PED-4395 (A) pupal exuvim of *Mycetobia* “morphotype 1”. (B) *Mycetobia connexa*,** female. (C) partial beetle (Coleoptera).

10.7717/peerj.7843/supp-12 Figure S12 Fossil pupa, *Mycetobia connexa* (*Mycetobia* “morphotype 1”), GPIH collection number AKBS-00071 (A) habitus, ventro-lateral view. (B) abdomen, dorsal view.

10.7717/peerj.7843/supp-13 Figure S13 Fossil pupa, *Mycetobia* “morphotype 1” with syninclusions, DEI, collection number Dip-00651 (A) habitus, lateral view. (B) dipteran non-biting midge (Chrionomidae, Orthocladiinae). (C) fly (Sciaroidea).

10.7717/peerj.7843/supp-14 Figure S14 Fossil pupa,* Mycetobia connexa* (*Mycetobia*“morphotype 1”) with syninclusions, GPIH, collection number 1851-DN (A) pupa (exuvium), *Mycetobia* “morphotype 1” and fungus gnat (Keroplatidae) male. (B) fly (Sciaridae) male. (C) fly (Bibionomorpha, probably Anisopodidae).

10.7717/peerj.7843/supp-15 Figure S15 Fossil pupa (exuvium), *Mycetobia connexa* (*Mycetobia*“morphotype 1”) with syninclusions, collection number PED-4395 (A) Overview. (B) *Mycetobia connexa* male. (C) *Mycetobia connexa* male, distal part of metathoracic tibia. 1, *Mycetobia connexa* male; 2, *Mycetobia connexa* female; 3, pupal exuvium of* M. connexa*.

10.7717/peerj.7843/supp-16 Figure S16 Fossil pupae, *Mycetobia* “morphotype 1” (A) DEI, collection number Dip-00657, dorsal view. (B) DEI, collection number Dip-00659, lateral view.

10.7717/peerj.7843/supp-17 Figure S17 Fossil pupa, *Mycetobia* “morphotype 1”, DEI, collection number Dip-00657 (Bitterfeld amber) (A) habitus, dorsal view. (B) habitus, ventral view.

10.7717/peerj.7843/supp-18 Figure S18 Fossil pupa, *Mycetobia* “morphotype 1”, DEI, collection number Dip-00655 (A) habitus, dorsal view. (B) habitus, ventro-lateral view.

10.7717/peerj.7843/supp-19 Figure S19 Fossil pupa, *Mycetobia* “morphotype 1” with syninclusion, DEI, collection number Dip-00655 (specimen 2) (A) habitus, lateral view. (B) habitus, ventro-lateral view. (C) fly (Diptera, Sciaridae).

10.7717/peerj.7843/supp-20 Figure S20 Fossil pupa (exuvium), *Mycetobia* “morphotype 1” collection number PED-4998 (A) habitus, ventral view. (B) habitus, dorsal view.

10.7717/peerj.7843/supp-21 Figure S21 Fossil pupa (exuvium), *Mycetobia* “morphotype 1” (Bitterfeld amber), collection number Dip-00661 (A) habitus, ventral view. (B) habitus, dorsal view, (C) habitus, lateral view.

10.7717/peerj.7843/supp-22 Figure S22 Fossil pupa, *Mycetobia* “morphotype 1” (Bitterfeld amber), DEI, collection number Dip-00650 (A) habitus, dorsal view. (B) habitus, ventral view.

10.7717/peerj.7843/supp-23 Figure S23 Fossil pupa, *Mycetobia* “morphotype 1” and syninclusions, GPIH, N-7095 A) overview. (B) pupa (upper left) *Mycetobia* “morphotype 1”, (upper left) and larva of Neuroptera; lower right). (C, D) adult long-legged fly (Dolichopodidae). (C) specimen 1 (D) specimen 2.

10.7717/peerj.7843/supp-24 Figure S24 Fossil pupa (exuvium), *Mycetobia* “morphotype 1”, DEI, collection number Dip-00653 (A) habitus, dorsal view. (B) habitus, ventral view. (C) habitus, lateral view.

10.7717/peerj.7843/supp-25 Figure S25 Fossil pupa (exuvium), *Mycetobia* “morphotype 1”, rendering of µ-CT scans, DEI, collection number Dip-00653 (A) habitus, dorsal view. (B) habitus, ventral view. (C) habitus, lateral view. MicroCT scanning credit: Marie Hörnig.

10.7717/peerj.7843/supp-26 Figure S26 Fossil pupa (exuvium), *Mycetobia* “morphotype 1”, rendering of µ-CT scans , MfNB, collection number MB.I.7295 (A) habitus, dorsal view. (B) habitus, lateral view. (C) habitus, ventral view. (D) habitus, lateral view. All images red-blue stereo anaglyphs, please use red-cyan glasses to view.

10.7717/peerj.7843/supp-27 Figure S27 Fossil *Mycetobia* pupa** and syninclusions Fossil pupae, *Mycetobia* and syninclusions. (A)“morphotype 1” and syninclusions, GPIH, collection number AKBS-00071. 1, largely unidentifiable (Insecta); 2, 3, 5–9, 13, 15 ant worker (*Lasius schiefferdeckeri* Mayr, 1868); 4, Fossil pupa, *Mycetobia* “morphotype 1”; 10 ant worker (*Ctenobethylus goepperti* (Mayr, 1868)). (B) syninclusions to “morphotype 2”, PED, collection number PED-4866; adult rove beetle (Coleoptera: Staphylinidae), two adult gall midges (Diptera; Cecidomyiidae). (C) pupa of *Mycetobia* “morphotype 2”, GPIH, collection number L-7514, habitus, ventral view.

10.7717/peerj.7843/supp-28 Figure S28 Fossil pupa (pharate adult), *Mycetobia* “morphotype 3”, rendering of µ-CT scans, DEI, collection number Dip-00660 (A) habitus, lateral view, right body side, mirrored. (B) habitus, lateral view, left body side. (C) habitus, dorsal view. (D) habitus, ventral view. MicroCT scanning credit: Marie Hörnig.

10.7717/peerj.7843/supp-29 Figure S29 Fossil pupa (pharate adult), *Mycetobia* “morphotype 3” DEI, collection number Dip-00652 (A) habitus, dorsal view. (B) habitus, ventral view.

10.7717/peerj.7843/supp-30 Data S1 Morphometric data of Mycetobia larvae

10.7717/peerj.7843/supp-31 Data S2 Morphometric data of Mycetobia pupae

## Additional Information and Declarations

### Data Availability

The following information was supplied regarding data availability: The raw morphometric data are available in the Supplemental Files. The locations and the accession numbers of all specimens are as follows: Specimens PED-4395, PED-4748, PED-4866, PED-4965, PED-4970, PED-4998, PED-5695 are held at the collection of the Palaeo-Evo-Devo Research Group, Ludwig-Maximilians-Universtät, Munich, Germany (PED); specimens Dip-00639 through Dip-00642 and Dip-00649 through Dip-00664 are held in the collection of the Entomologische Institut Senckenberg- Münchenberg (DEI); specimens GPIH-Schlee-0024, BI-2350, GPIH-3706 W, GPIH-L-7592, GPIH-Schlee-0247,AKBS-00071, GPIH-1851DN, GPIH-N-7095, GPIH-L-7514, GPIH -7516 are housed in the Center for Natural History in Hamburg (CeNak/ GPIH), specimen MB.I.7295 is housed in Museum for Natural History/Museum für Naturkunde Berlin (MfNB). Extant specimens from CeNak and ZSM are not given accession numbers because it is outside the operational practice of the institutions in question. Specimens can be identified by the combination of the taxon name and label, which are provided in the text. While we can get some numbers assigned, it would be rather strange to have these specimens as only non-type insect specimens in these collections bearing numbers. Labels as follows: ZSM: Mycetobia pallipes Meigen, 1818 >50 ZSM Ober-Bayern, Fürstenfeldbruck, Roßkastanie, Wundausfluß, Bayern, Germany, 29.5-4.7.1994, leg. W. Schlacht; Penthetria funebris Meigen, 1804. >50 ZSM Augsburg, Lechau nördl. St. Stephan, Barb-F.,Auwald- Ruderal, 440 m, 27.05.1981,Schmidt; CeNak: Bibio varipies Meigen, 1830. Extant specimens from CeNak and ZSM are not given acession numbers because it is outside operational practice of the institutions in question. Specimens can be identified by the combination of the taxon name and label, which are provided in the text. While we can get some numbers assigned, it would be rather strange to have this specimens as only non-type insect specimens in this collections bearing numbers.

### New Species Registration

The following information was supplied regarding the registration of a newly described species: Publication LSID: urn:lsid:zoobank.org:pub:7E6FFA31-9DA8-44A6-BE7D-55E6AE34B660 Dinobibio LSID: urn:lsid:zoobank.org:act:8C8DCD9A-1A44-473E-9692-54C7AE204B91 Dinobibio hoffeinseorum sp. nov. LSID: urn:lsid:zoobank.org:act:80D4F834-D0D4-404F-AE02-C8FF184D4943.
