# Supplementary figures and images for: Fly palaeo-evo-devo: immature stages of bibionomorphan dipterans in Baltic and Bitterfeld amber
Source: PeerJ. 2019 Oct 10;7:e7843. doi: 10.7717/peerj.7843 (PMC6790230; doi:10.7717/peerj.7843)

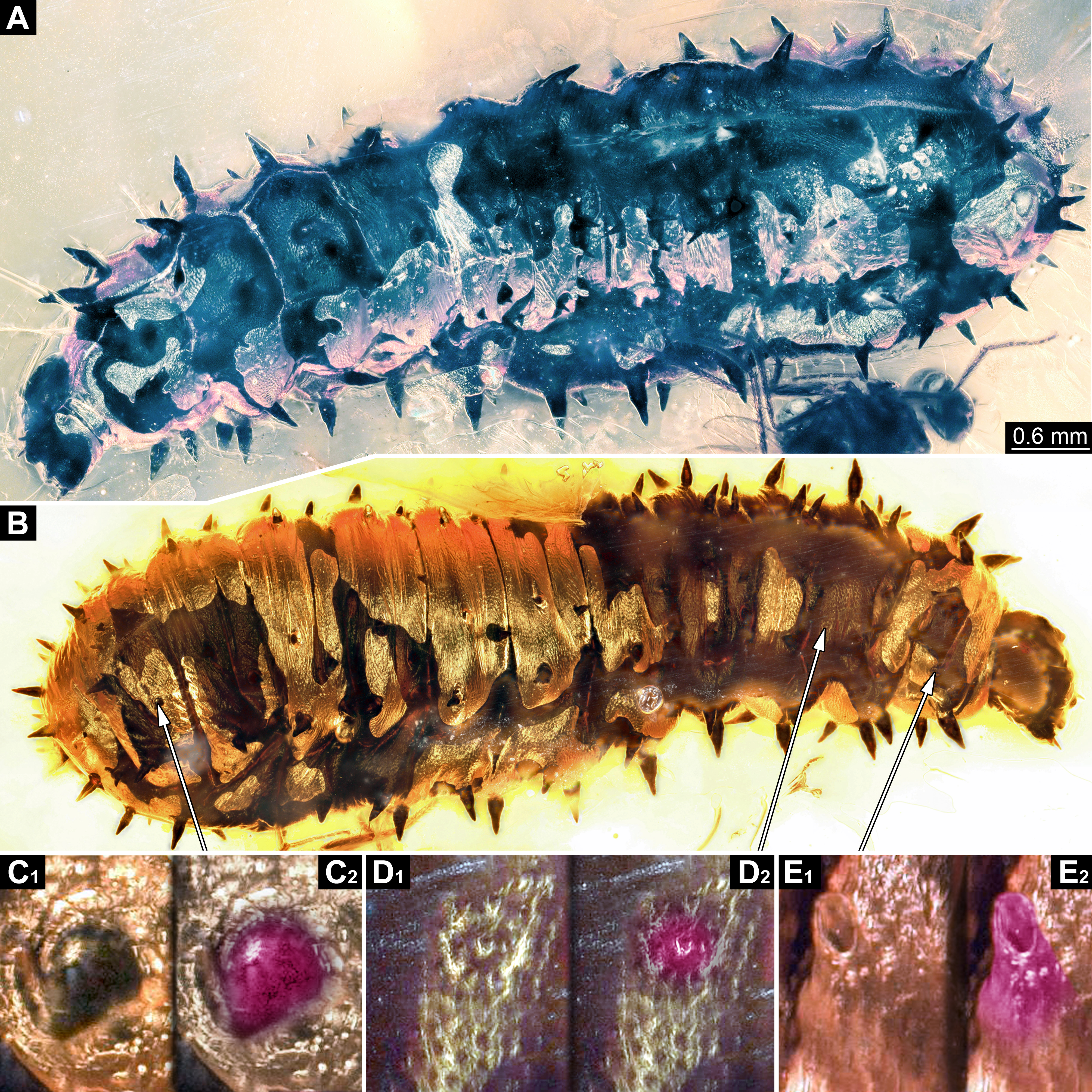

Supplement: Figure S1 — (A) ventro-lateral view. (B) dorso-lateral view; (C1–C2) spiracle 10. (D1–D2) spiracle 2. (E1–E2) spiracle 1. [file peerj-07-7843-s001.jpg]

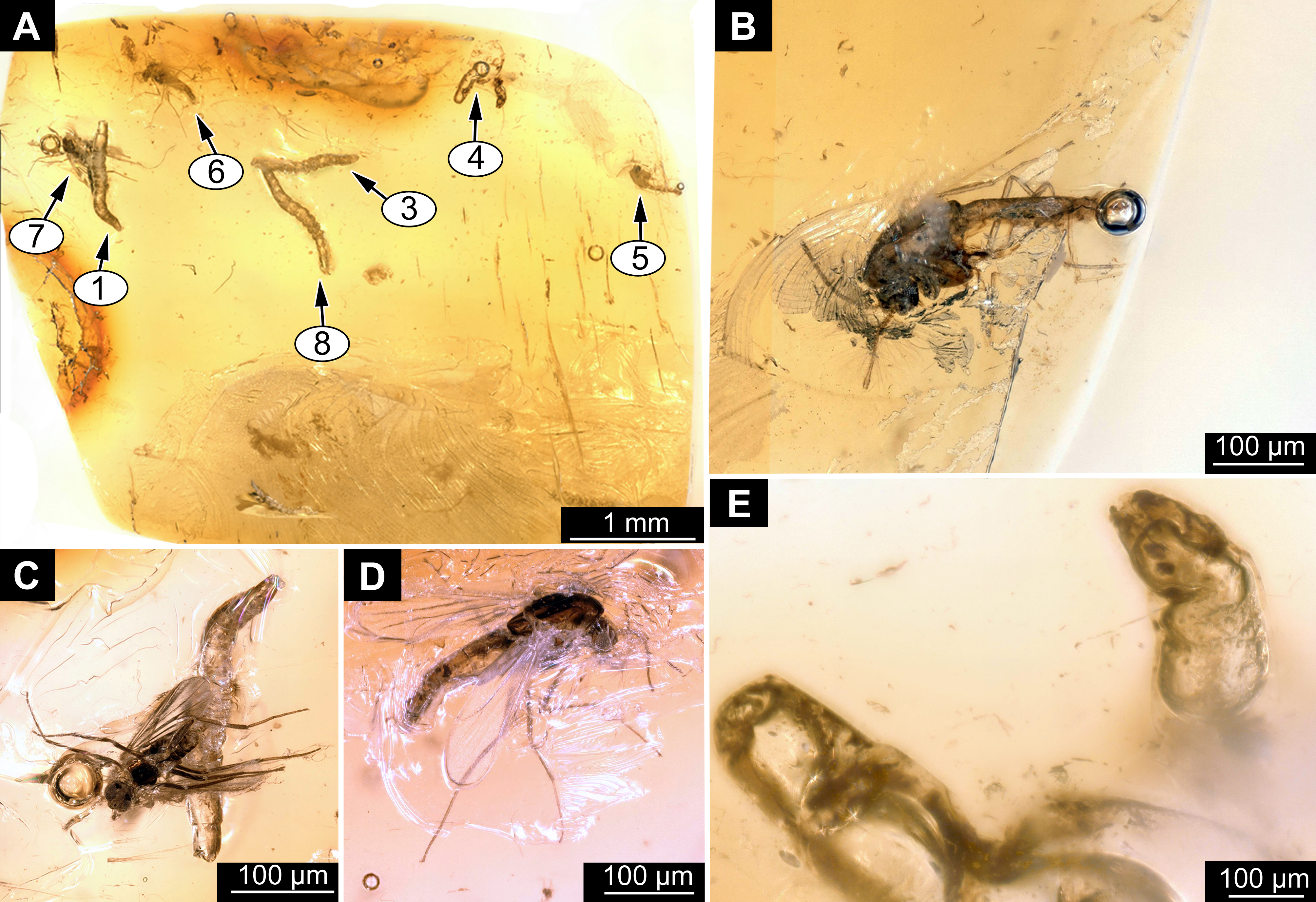

Supplement: Figure S2 — (A) overview of the amber piece. (B) caddisfly male, Polycentropodidae. (C) partial syninclusion of an adult beetle. 1–4, larvae of Mycetobia; 5, beetle; 6–10 larvae of Mycetobia; 11, caddisfly male, Polycentropodidae [file peerj-07-7843-s002.jpg]

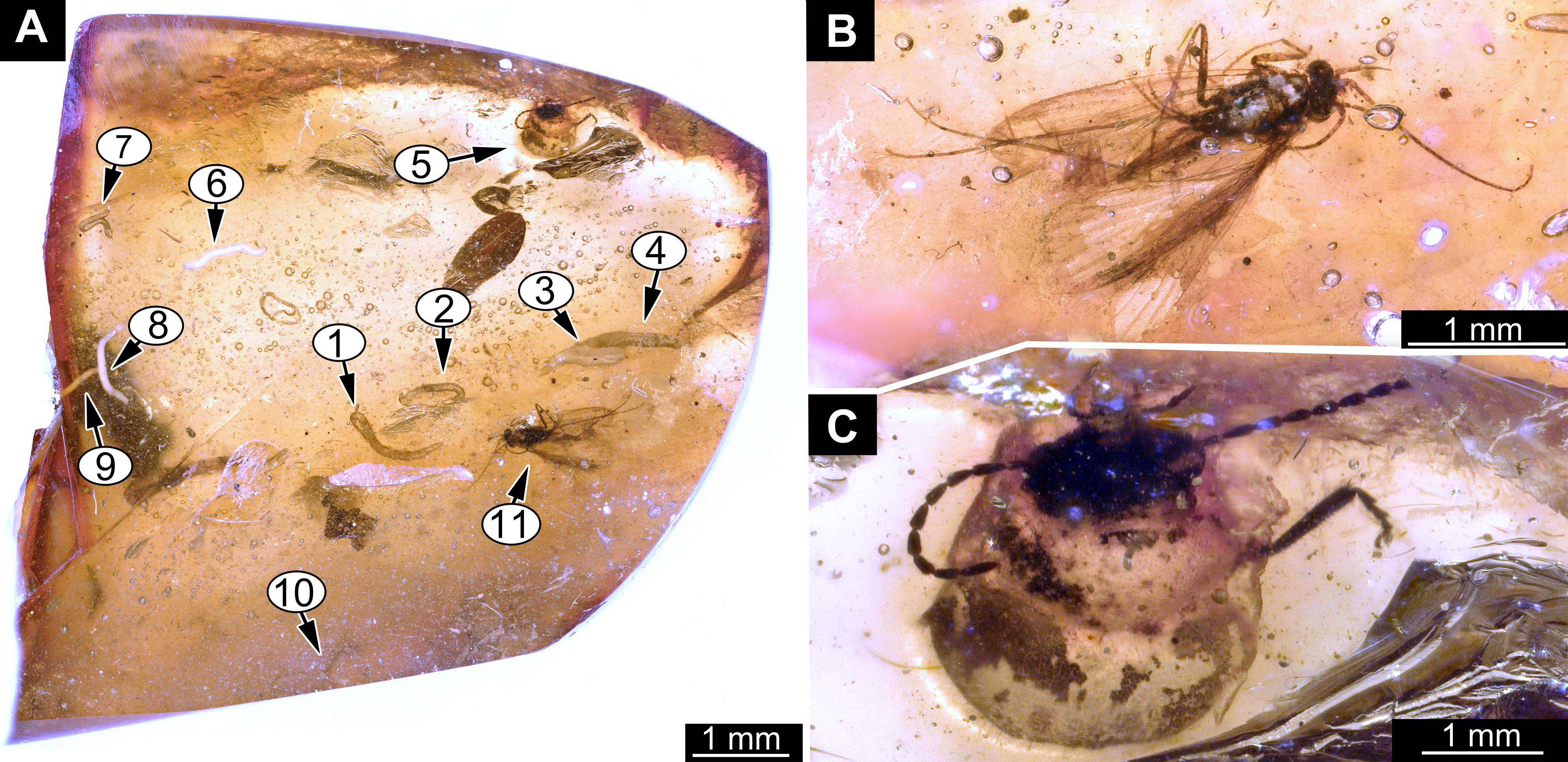

Supplement: Figure S3 — (A) Overview of the inclusions. (B–D) dipterans, non-biting midges (Chironomidae). (B) Rheosmittia pertenuis, male. (C) Orthocladiinae, female. (D) Rheosmittia pertenuis, male, second specimen. (E) partial inclusions of Mycetobia sp. larvae. 1–4 Mycetobia larvae; 5–6 R. pertenuis, males; 7 Orthocladiinae, female. [file peerj-07-7843-s003.jpg]

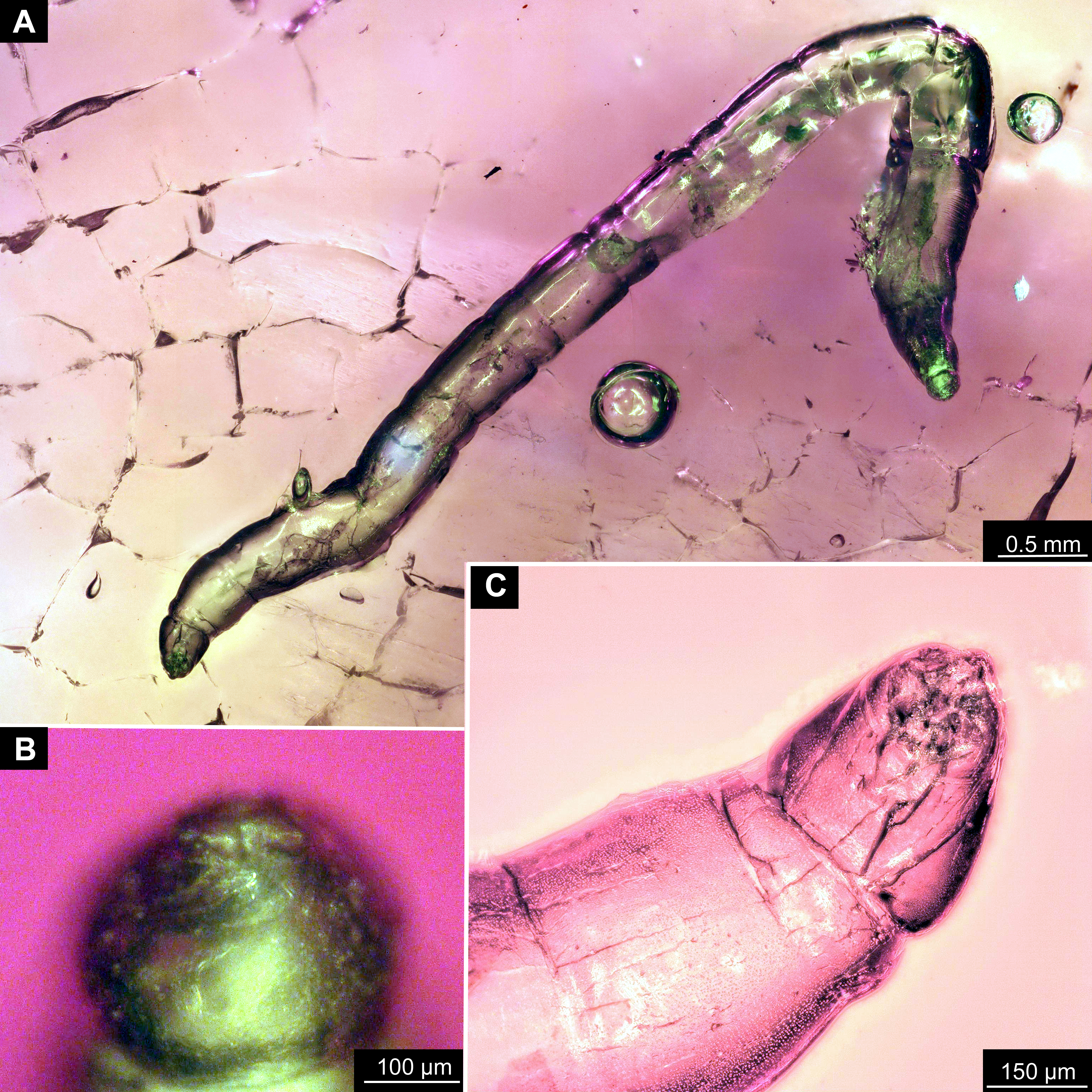

Supplement: Figure S4 — (A) habitus. (B) trunk end, with posterior spiracles. (C) head capsule, ventral view. [file peerj-07-7843-s004.jpg]

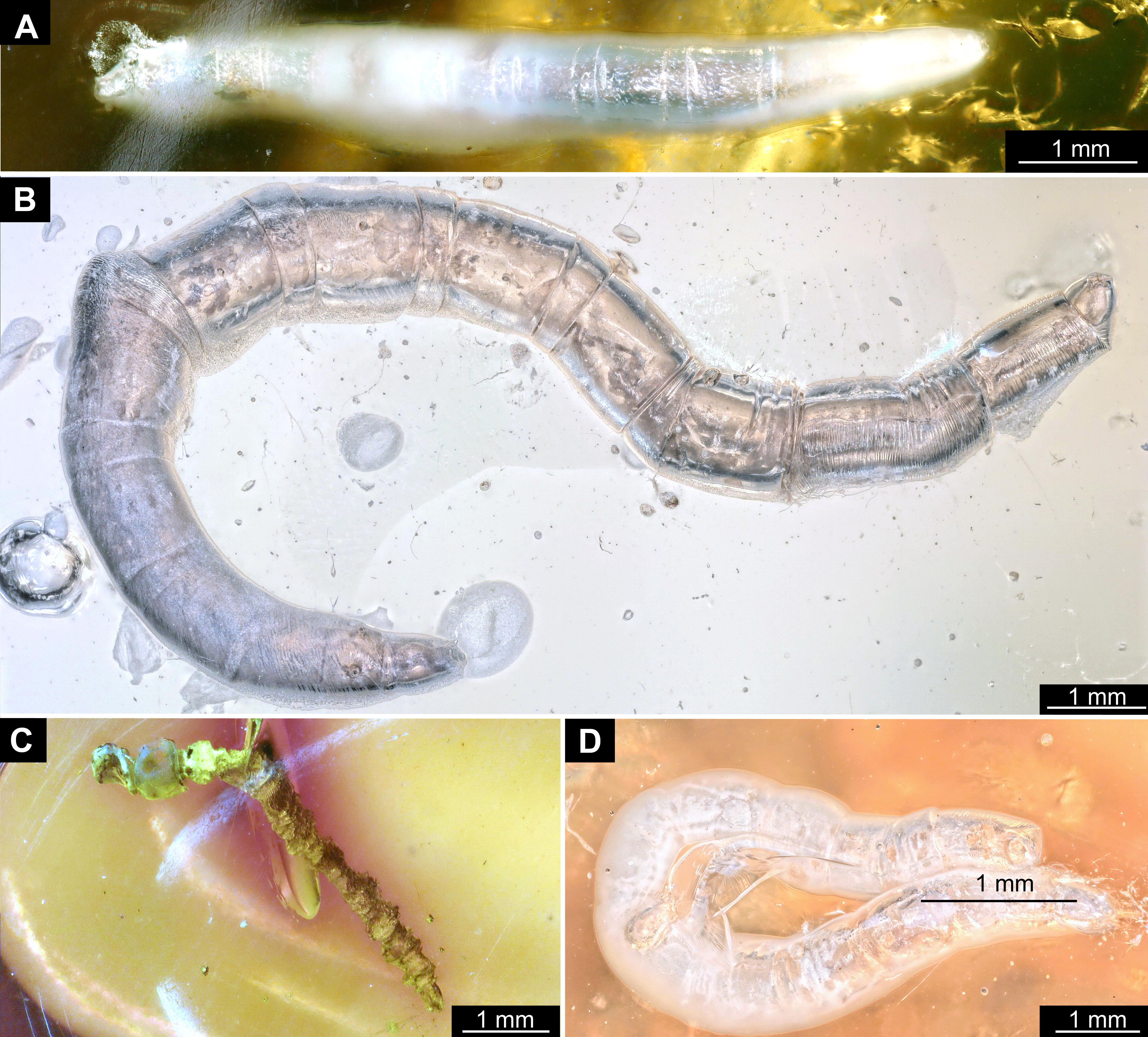

Supplement: Figure S5 — (A) PED-5695. (B) DEI, collection number Dip-00654. (C) GPIH (BI-2350). (D) PED-4965. [file peerj-07-7843-s005.jpg]

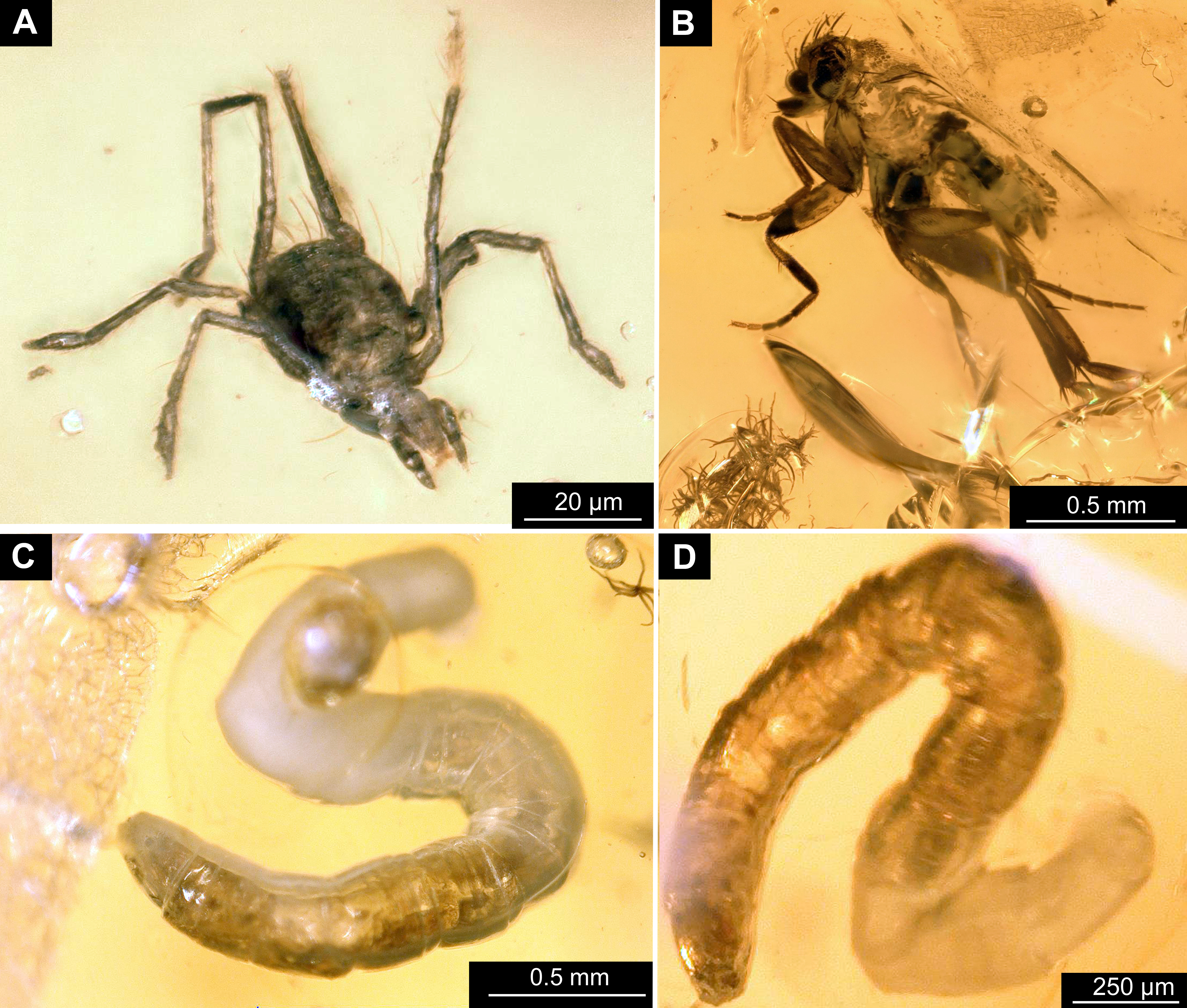

Supplement: Figure S6 — (A) mite. (B) fly, Phroidae. (C, D) larval specimen of Mycetobia. (C) ventral view. (D) dorsal view. [file peerj-07-7843-s006.jpg]

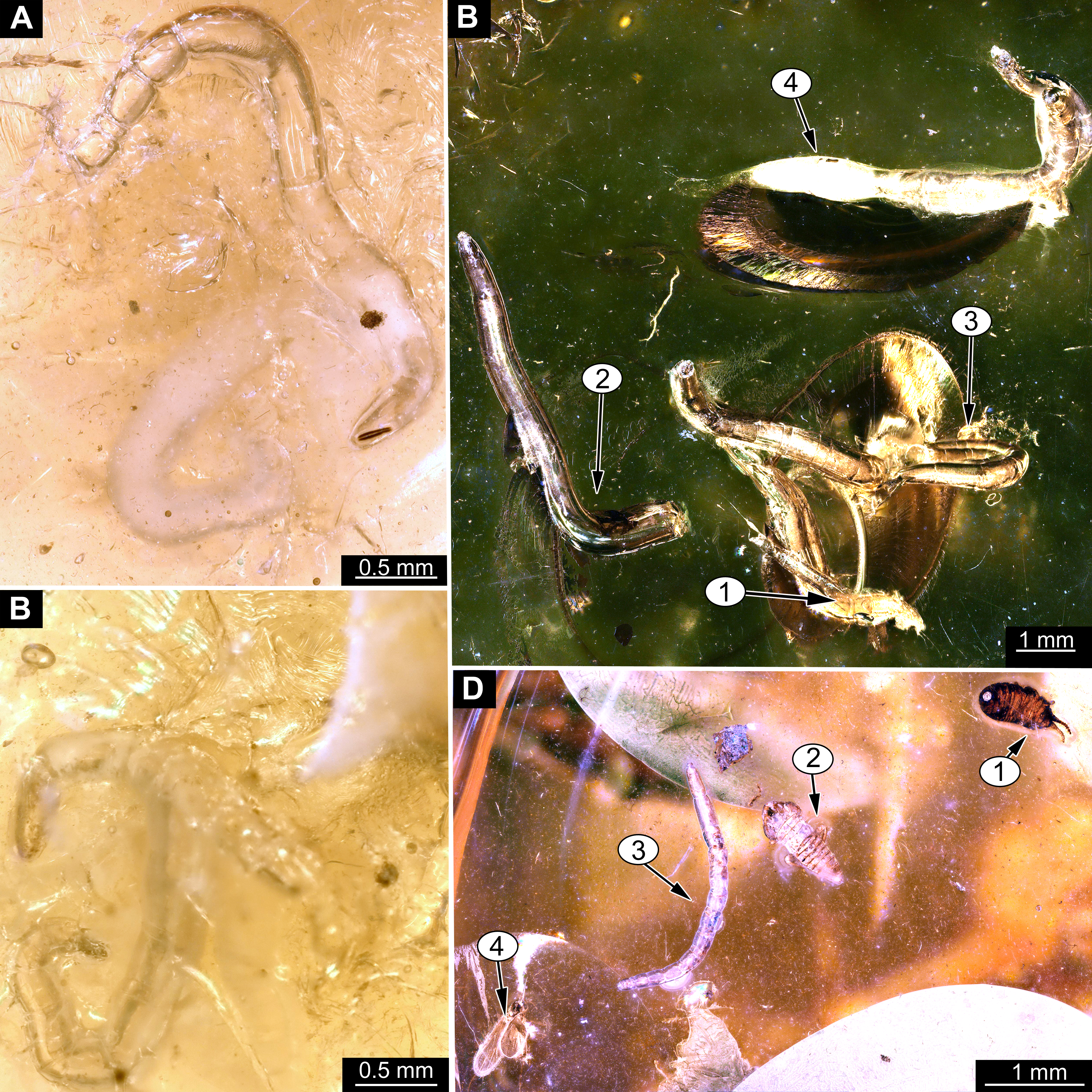

Supplement: Figure S7 — A) Two specimens, GPIH (L-7592). (B) two specimens, GPIH (L-7592). (C) four specimens (1–4), PED, collection number PED-4748. (D) larva with syninclusions, PED, collection number PED-4970. 1, scale insect, (Coccoidea), nymph; 2, leaf hopper (Cicadellidae), nymph; 3, larva, Mycetobia; 4, non-biting midge (Chironomidae), female. [file peerj-07-7843-s007.jpg]

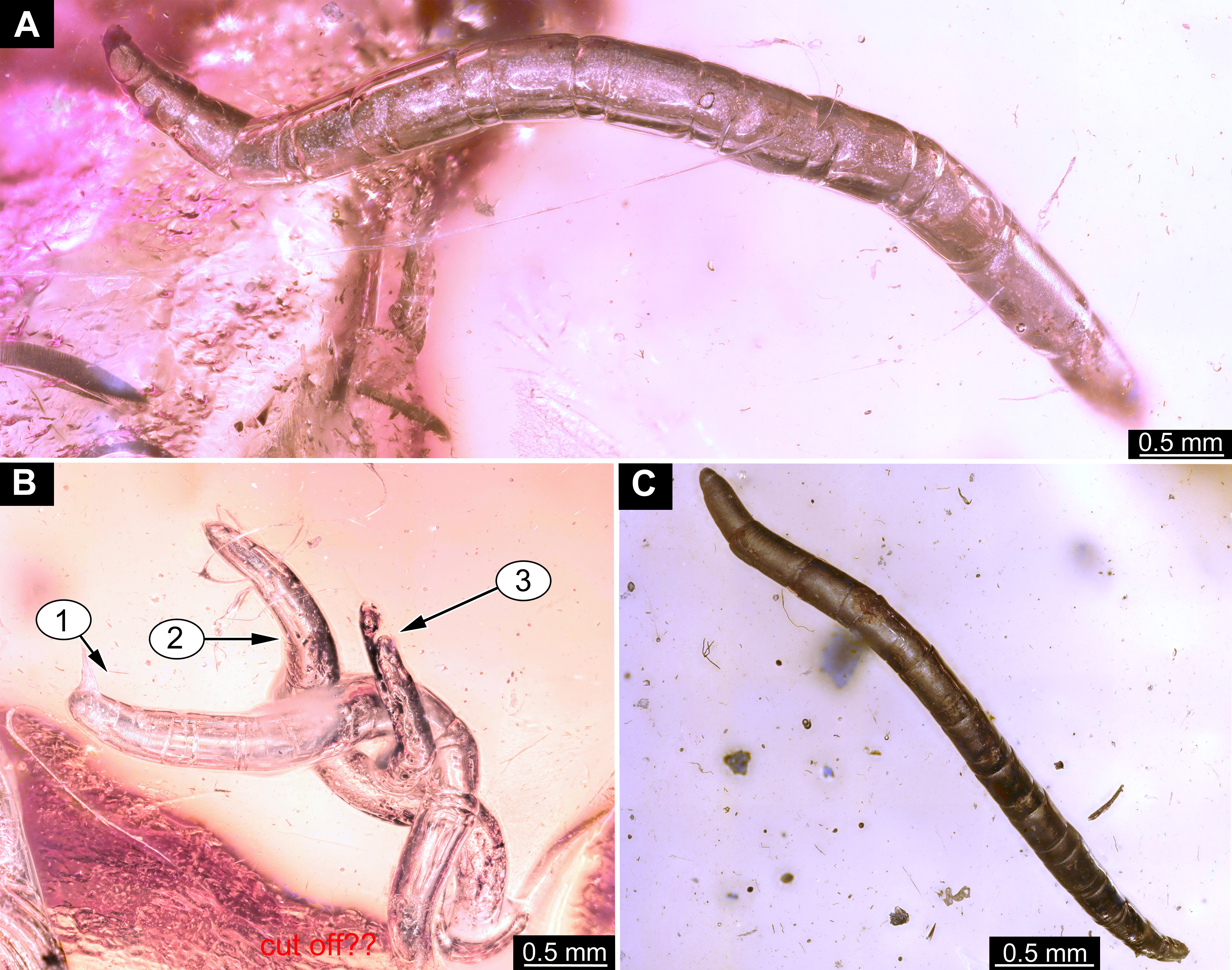

Supplement: Figure S8 — (A) large larva. (B) specimens 1–3. (C) large larva. [file peerj-07-7843-s008.jpg]

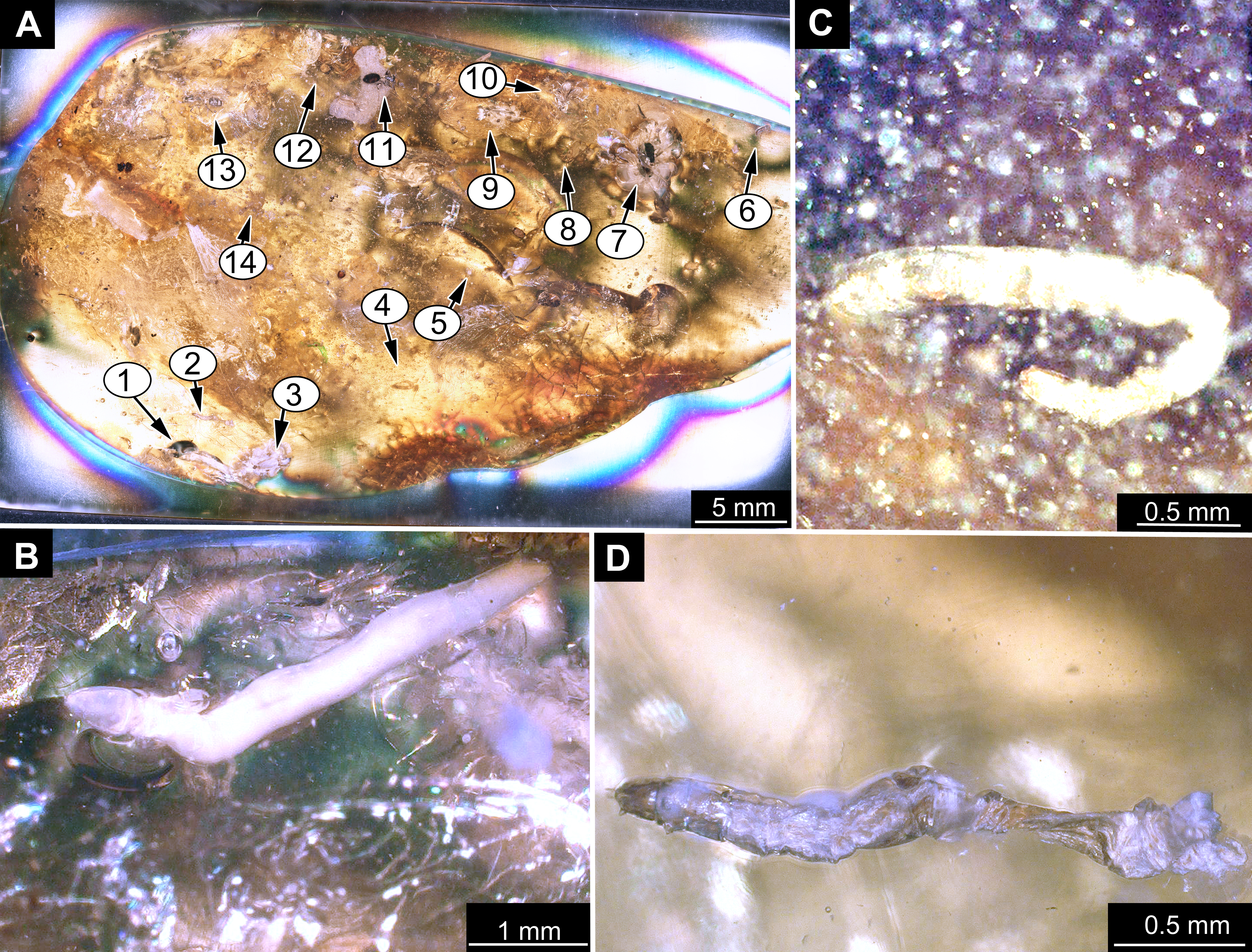

Supplement: Figure S9 — A) Overview of the amber piece Dip-00656from the collection of DEI. (B–D) larvae, Mycetobia. (B) specimen 1. (C) specimen 2. (D) specimen 3. 1, 2, 5, larva, Mycetobia; 3, 8, 10, 14 gall midges (Cecidomyiidae); 4, mite (Acari); 6, fly (“Acalyptrata”); 7, beetle (Coleoptera); 9, 11–13, ants (Fromicidae). [file peerj-07-7843-s009.jpg]

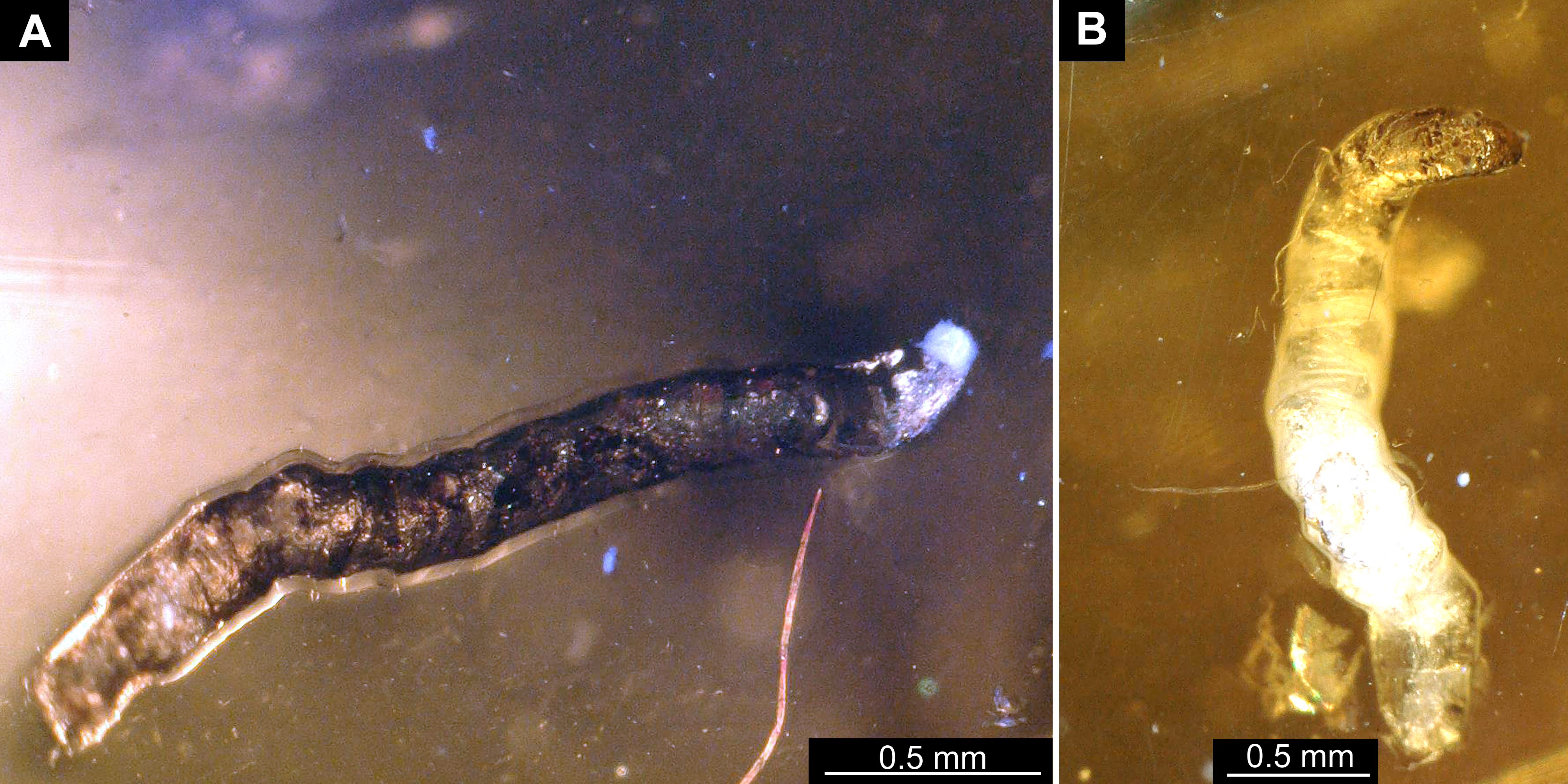

Supplement: Figure S10 — (A) specimen 1. (B) specimen 2. [file peerj-07-7843-s010.jpg]

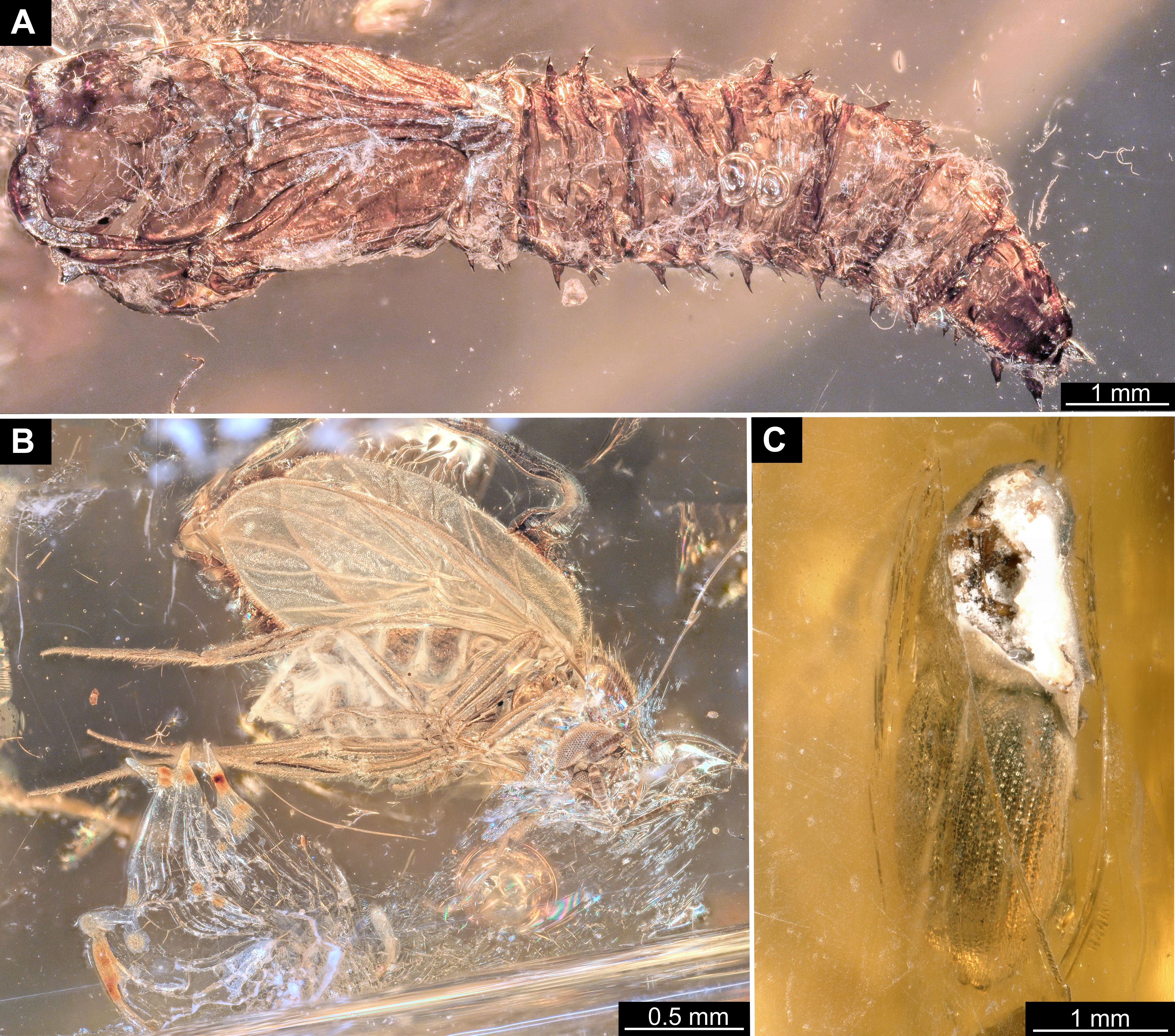

Supplement: Figure S11 — (A) pupal exuvim of Mycetobia “morphotype 1”. (B) Mycetobia connexa, female. (C) partial beetle (Coleoptera). [file peerj-07-7843-s011.jpg]

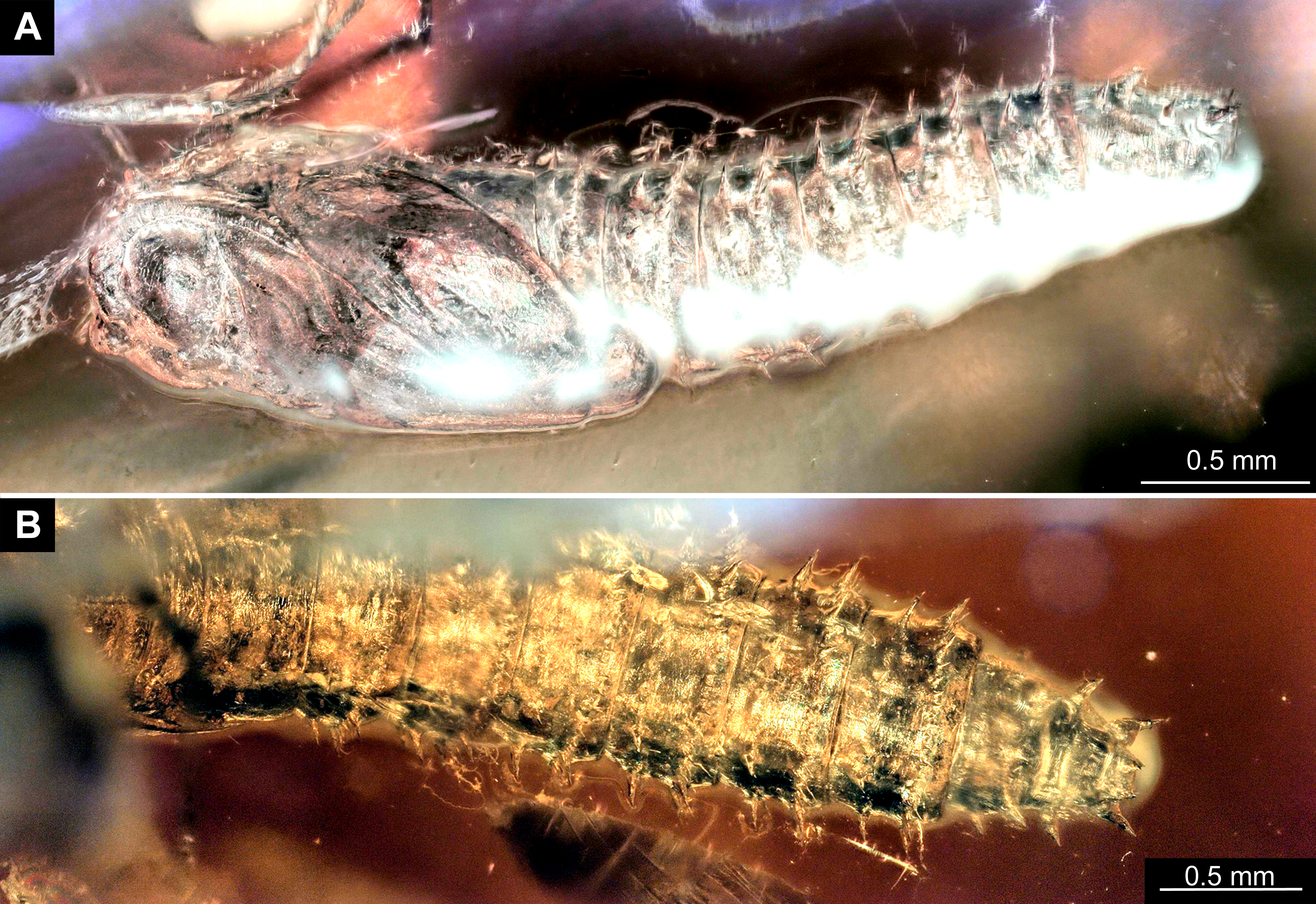

Supplement: Figure S12 — (A) habitus, ventro-lateral view. (B) abdomen, dorsal view. [file peerj-07-7843-s012.jpg]

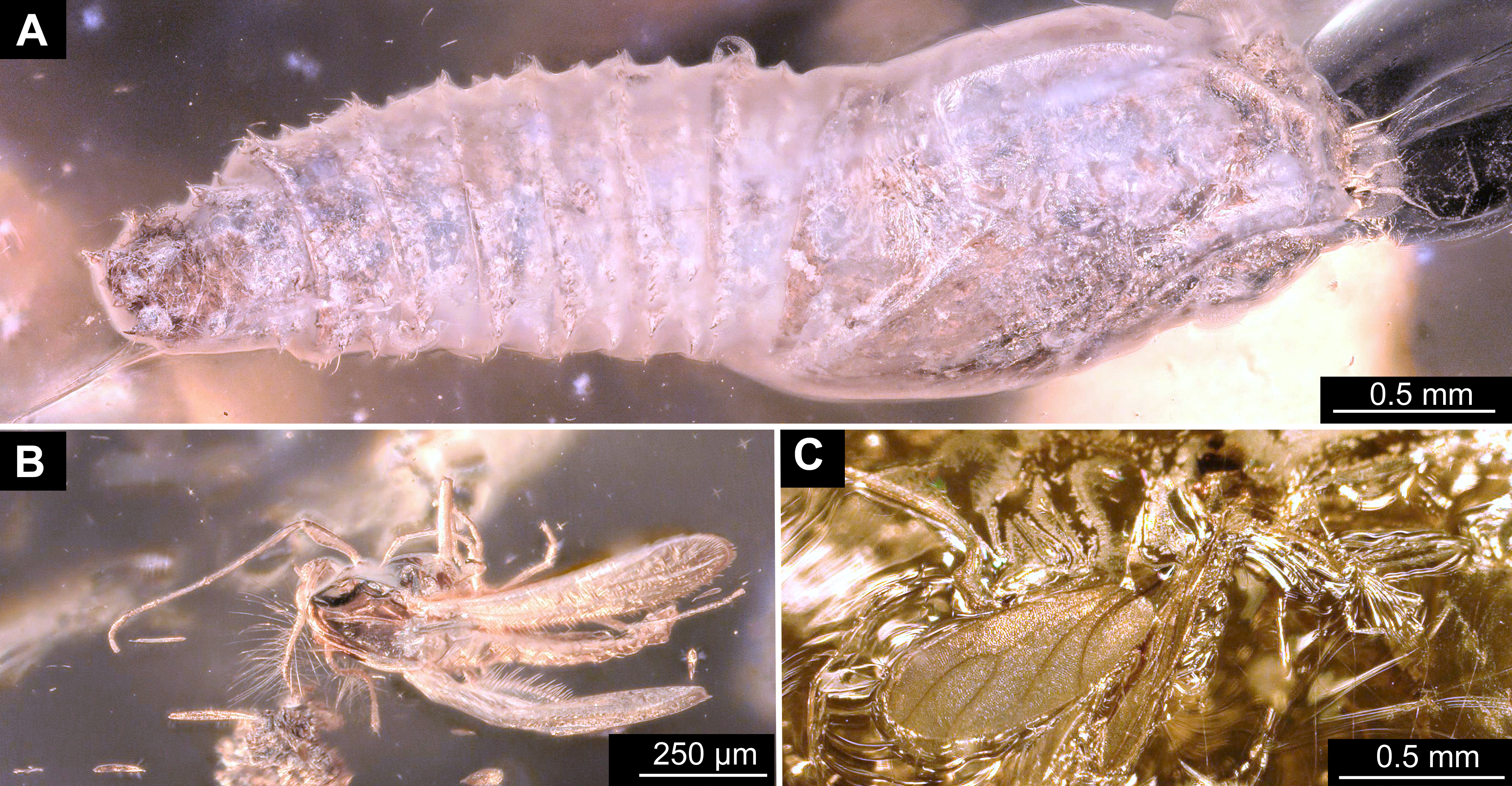

Supplement: Figure S13 — (A) habitus, lateral view. (B) dipteran non-biting midge (Chrionomidae, Orthocladiinae). (C) fly (Sciaroidea). [file peerj-07-7843-s013.jpg]

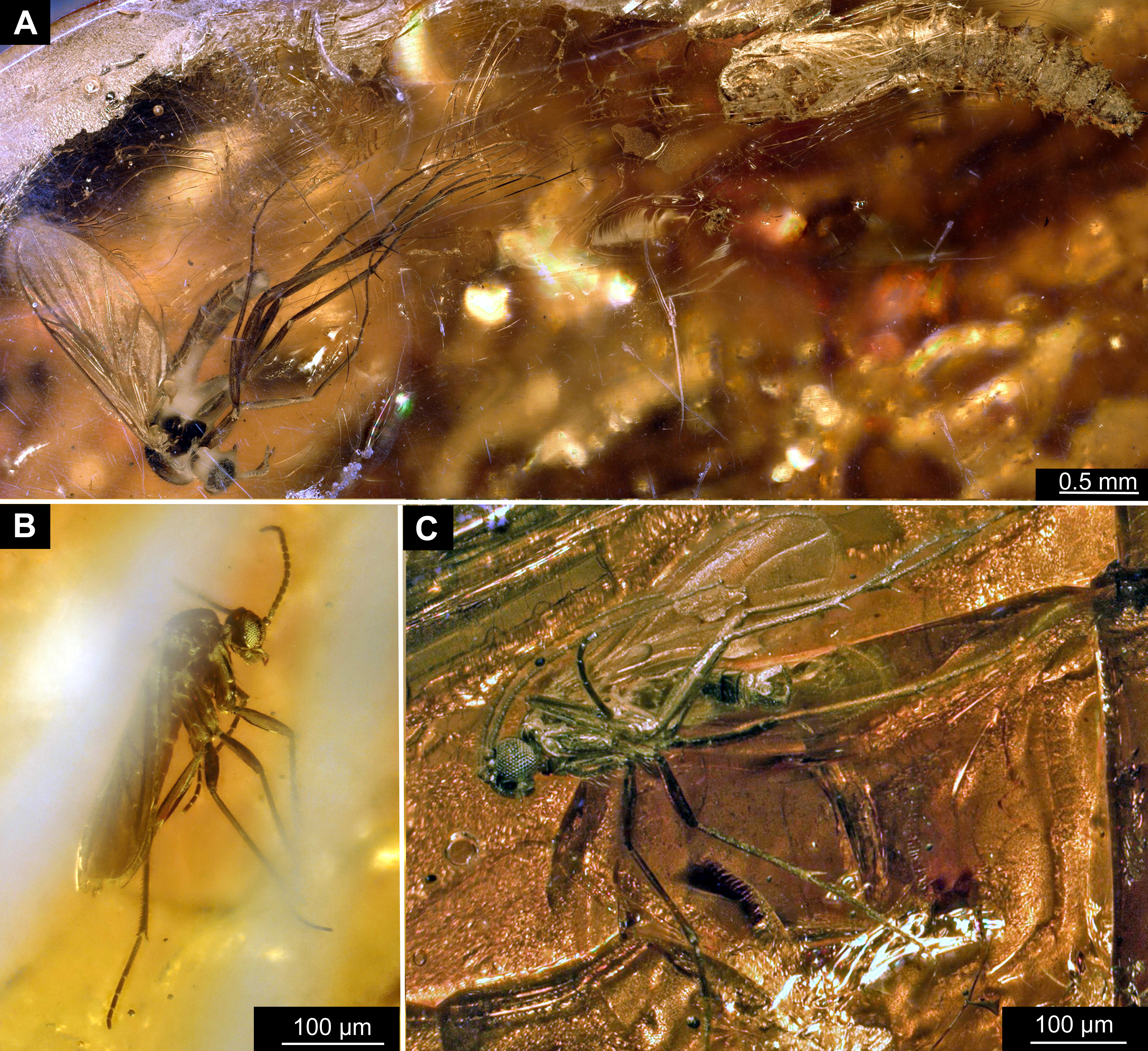

Supplement: Figure S14 — (A) pupa (exuvium), Mycetobia “morphotype 1” and fungus gnat (Keroplatidae) male. (B) fly (Sciaridae) male. (C) fly (Bibionomorpha, probably Anisopodidae). [file peerj-07-7843-s014.jpg]

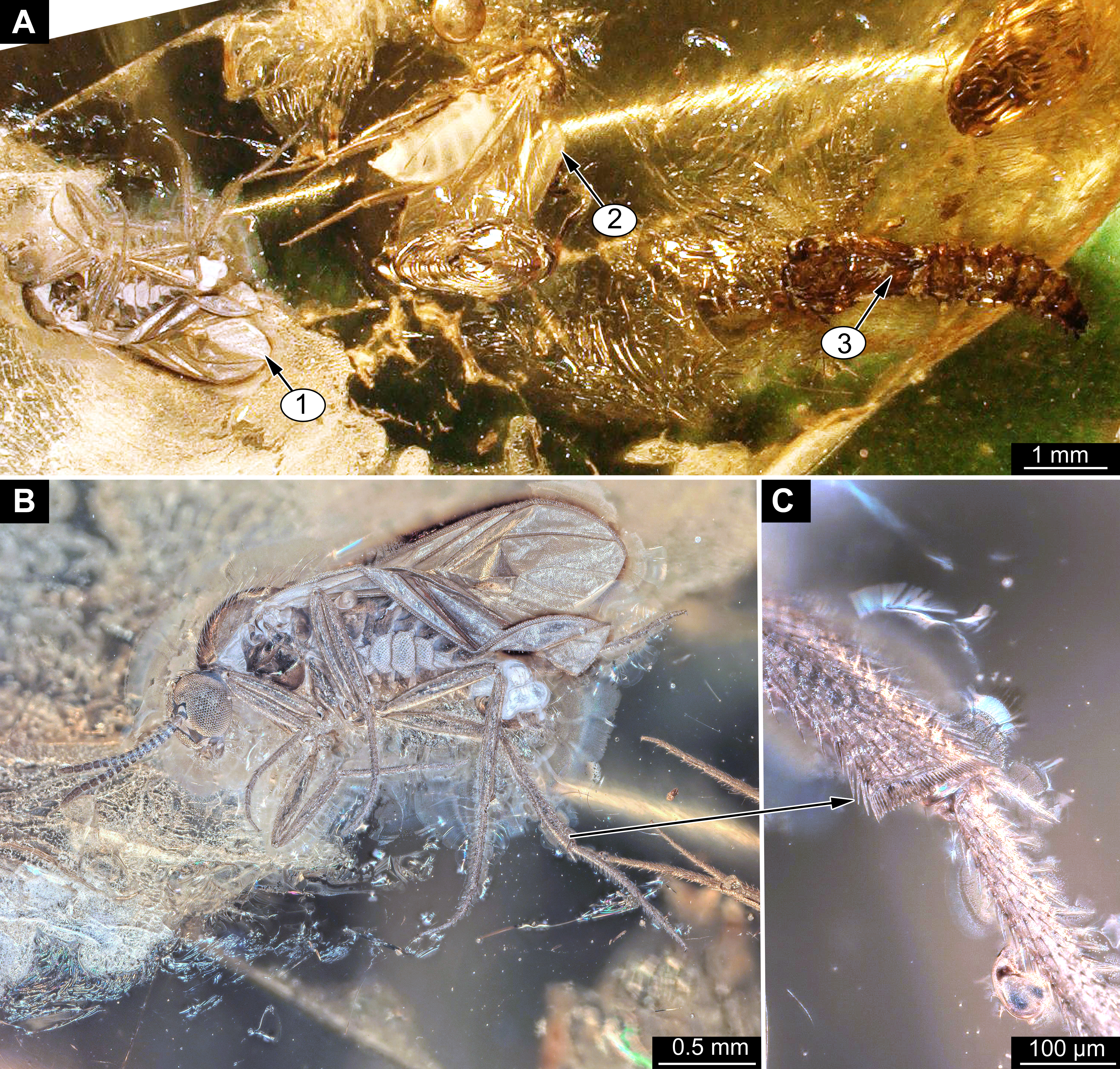

Supplement: Figure S15 — (A) Overview. (B) Mycetobia connexa male. (C) Mycetobia connexa male, distal part of metathoracic tibia. 1, Mycetobia connexa male; 2, Mycetobia connexa female; 3, pupal exuvium of M. connexa. [file peerj-07-7843-s015.jpg]

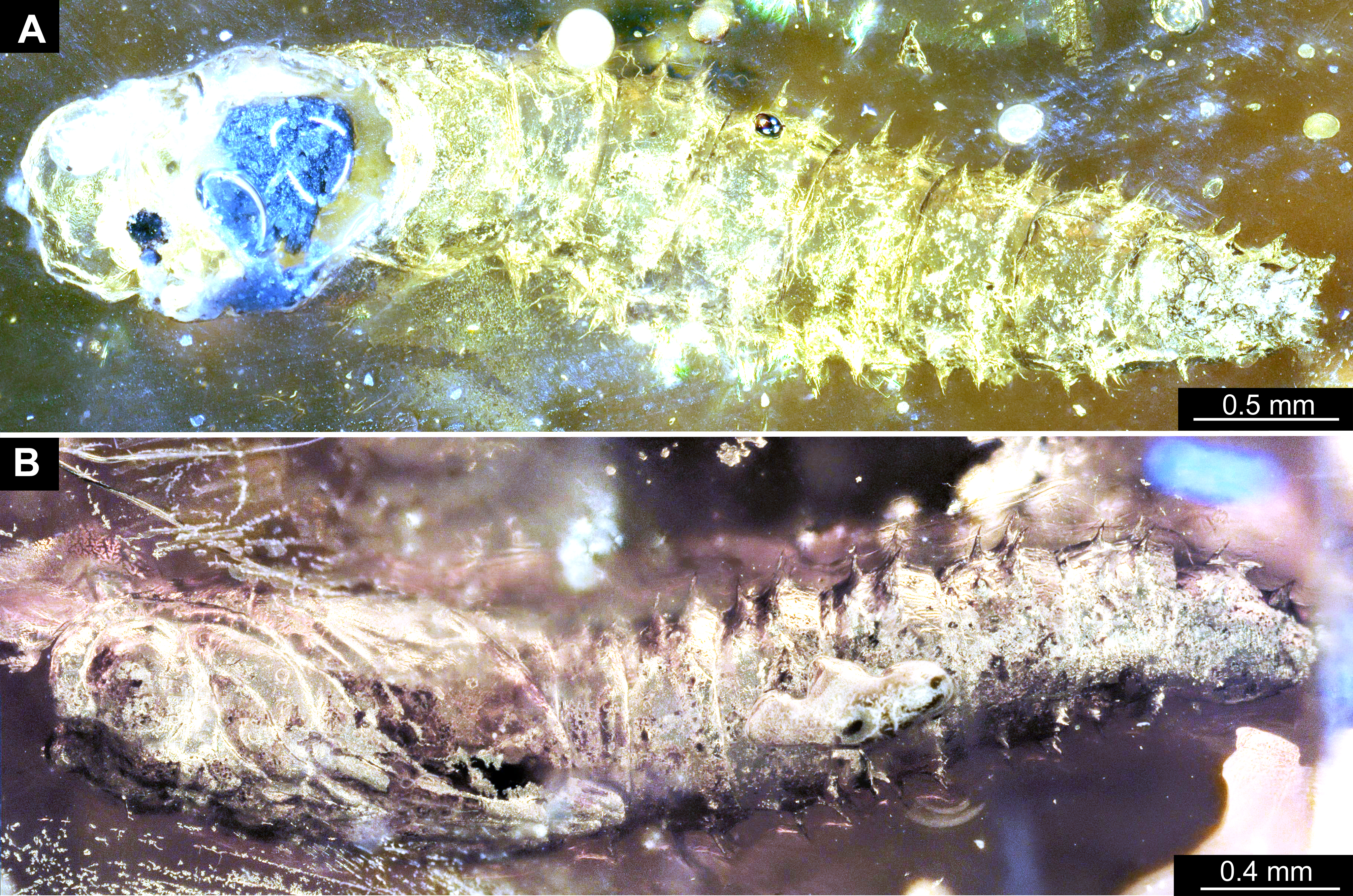

Supplement: Figure S16 — (A) DEI, collection number Dip-00657, dorsal view. (B) DEI, collection number Dip-00659, lateral view. [file peerj-07-7843-s016.jpg]

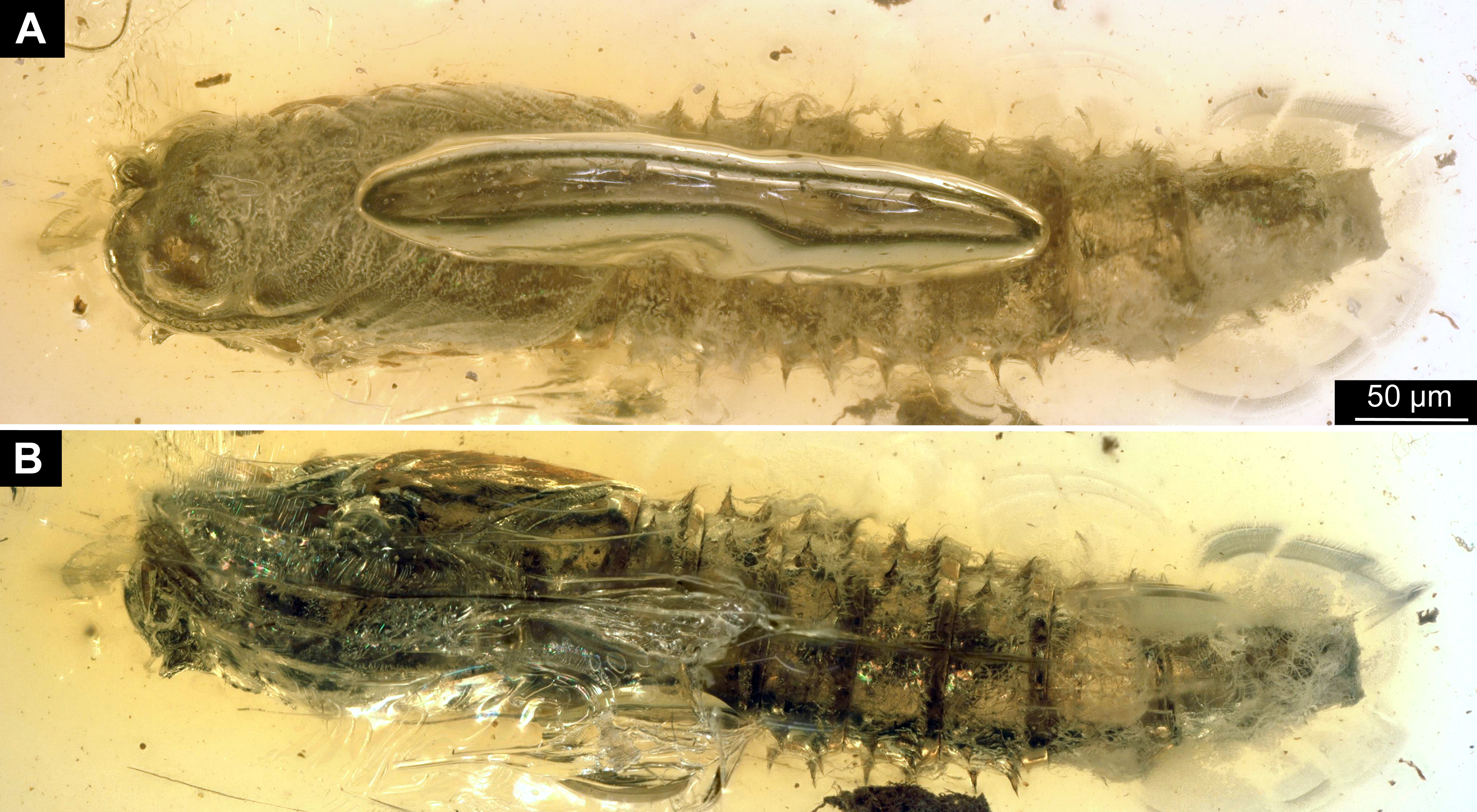

Supplement: Figure S17 — (A) habitus, dorsal view. (B) habitus, ventral view. [file peerj-07-7843-s017.jpg]

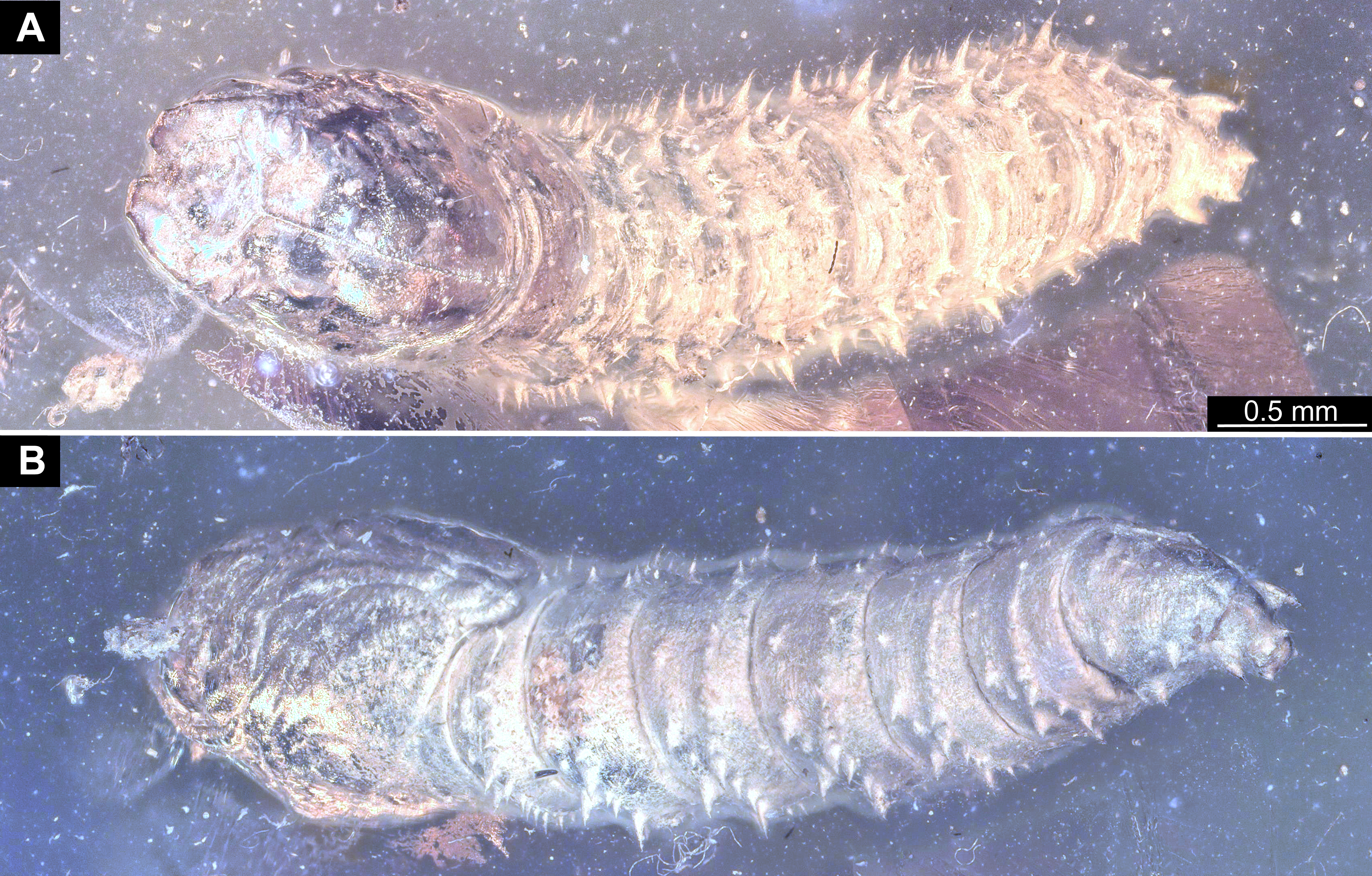

Supplement: Figure S18 — (A) habitus, dorsal view. (B) habitus, ventro-lateral view. [file peerj-07-7843-s018.jpg]

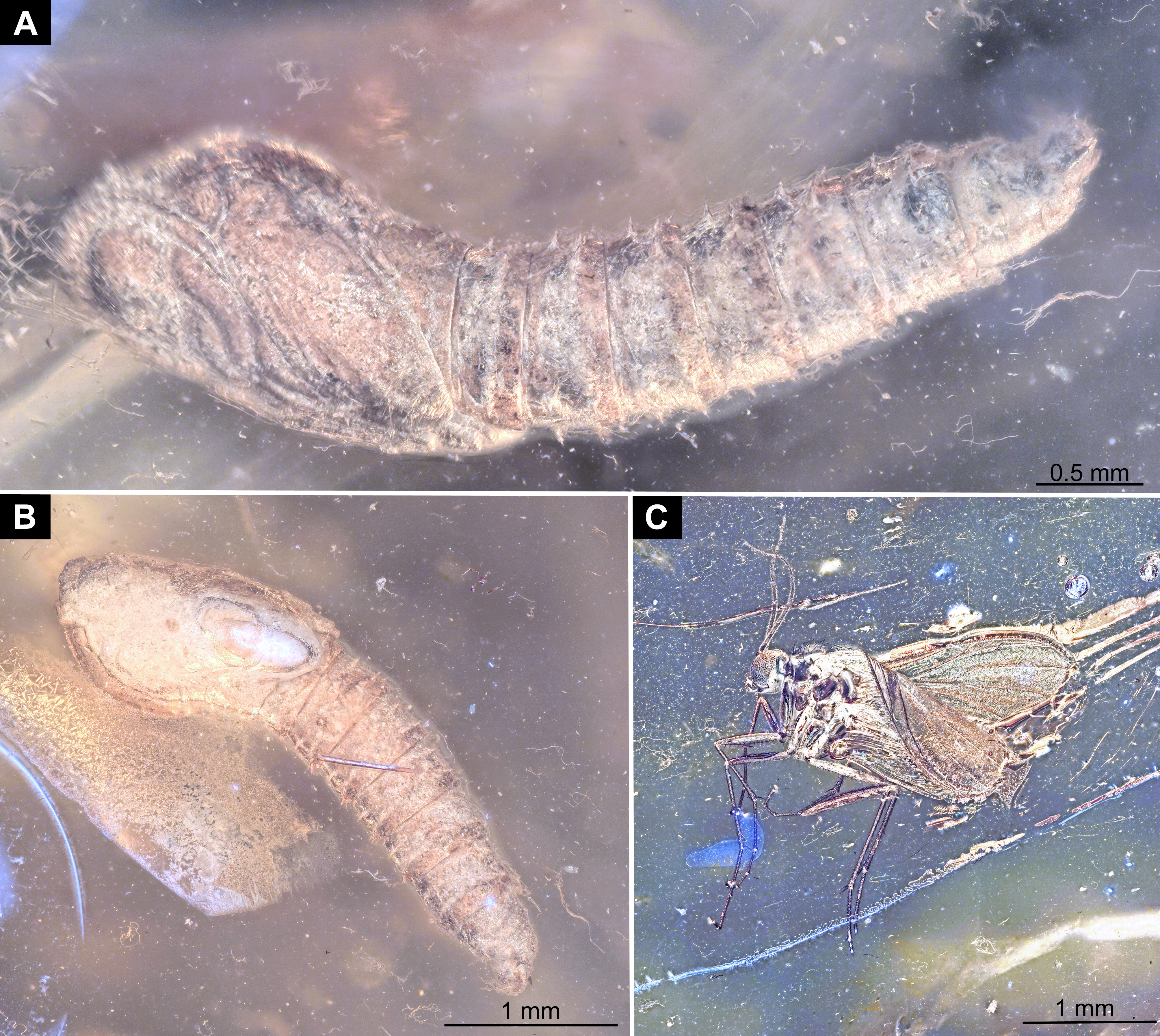

Supplement: Figure S19 — (A) habitus, lateral view. (B) habitus, ventro-lateral view. (C) fly (Diptera, Sciaridae). [file peerj-07-7843-s019.jpg]

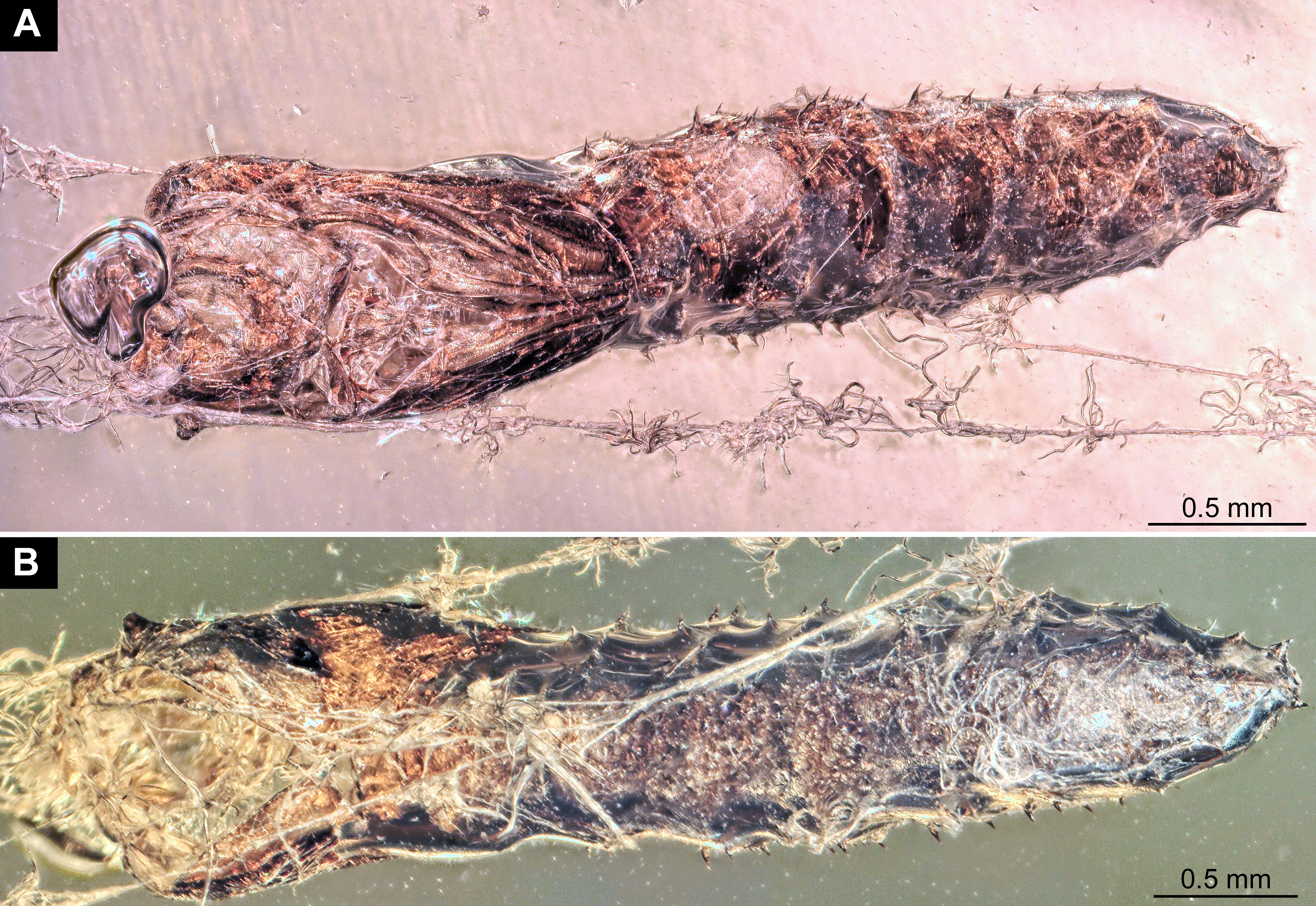

Supplement: Figure S20 — (A) habitus, ventral view. (B) habitus, dorsal view. [file peerj-07-7843-s020.jpg]

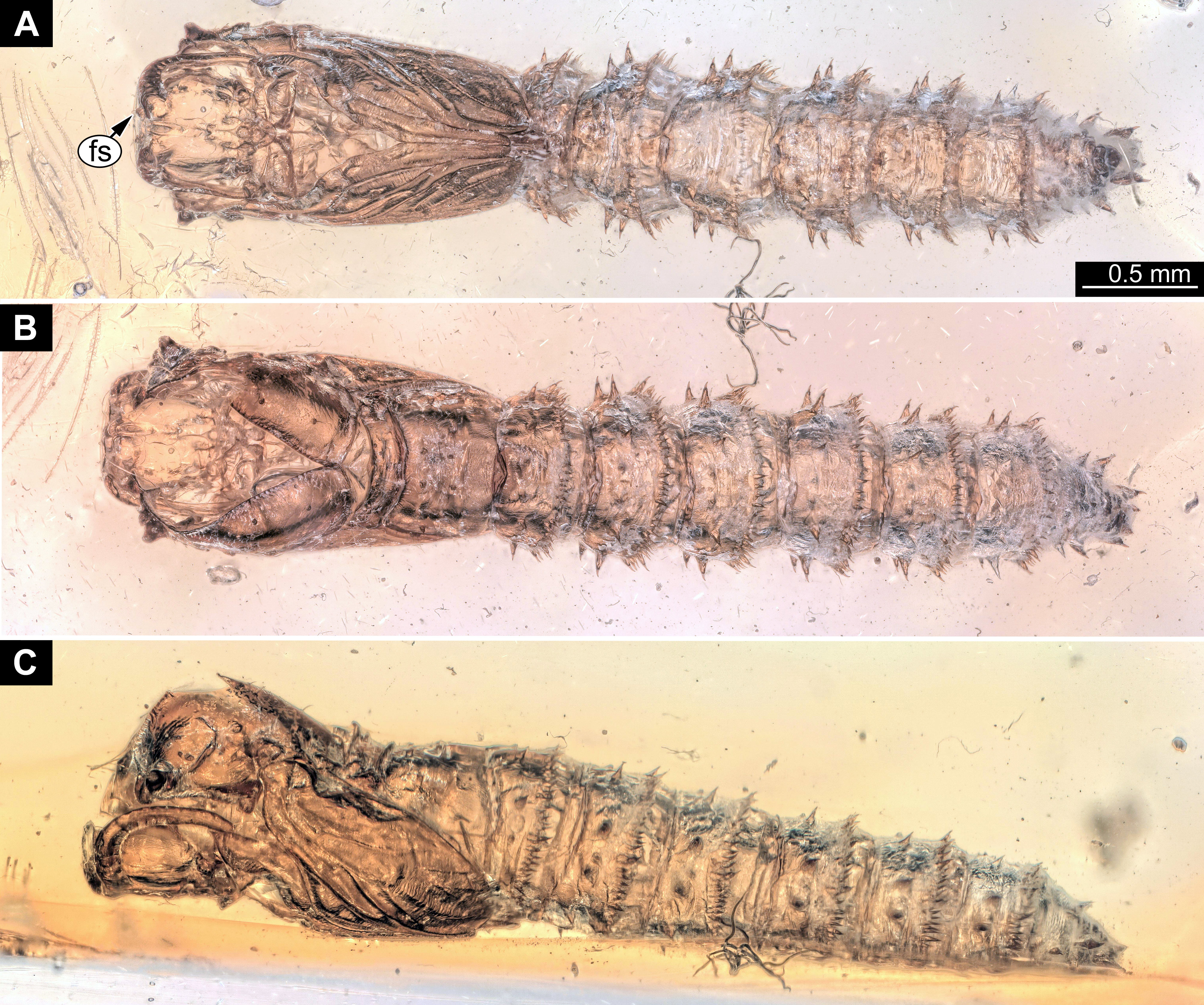

Supplement: Figure S21 — (A) habitus, ventral view. (B) habitus, dorsal view, (C) habitus, lateral view. [file peerj-07-7843-s021.jpg]

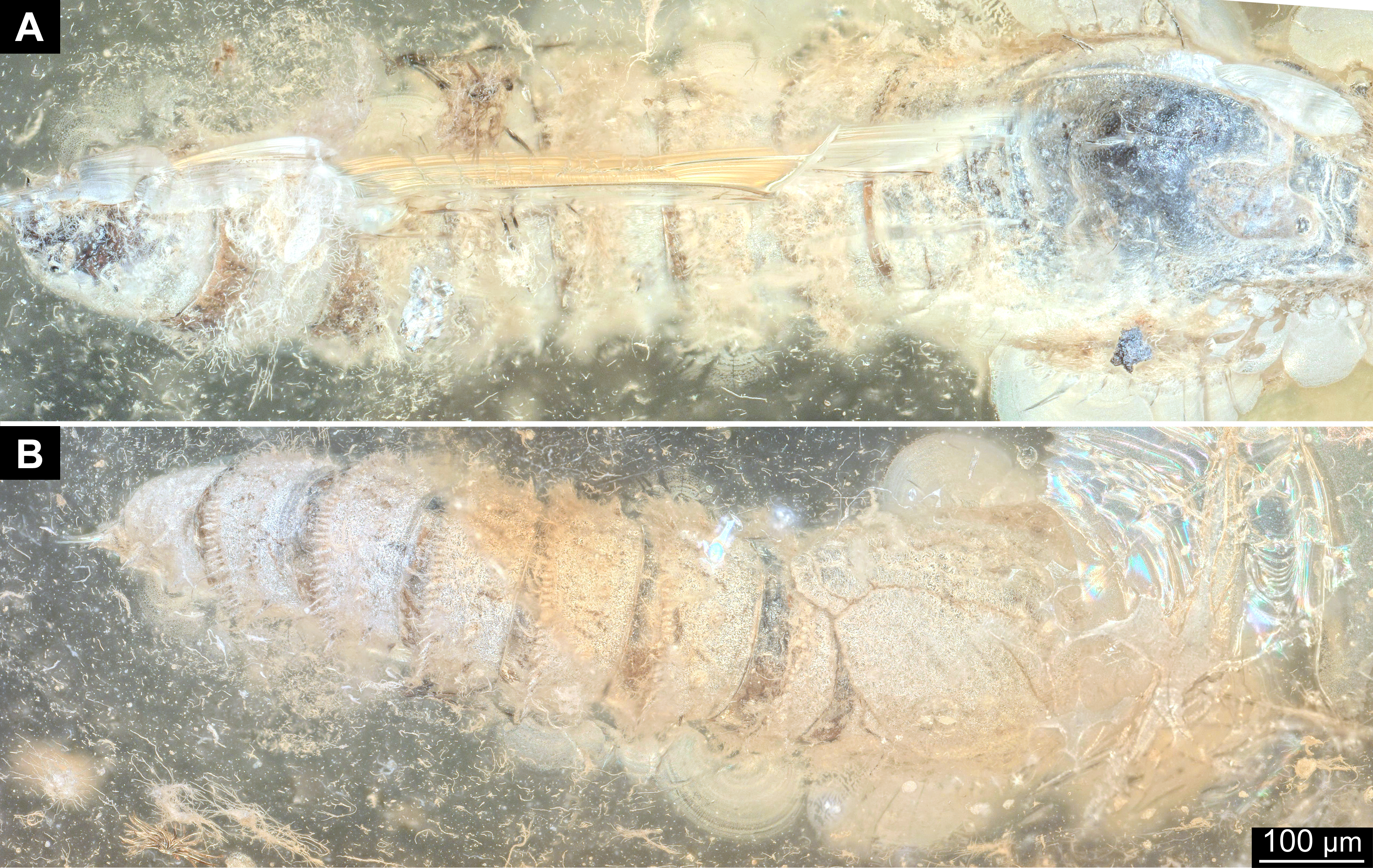

Supplement: Figure S22 — (A) habitus, dorsal view. (B) habitus, ventral view. [file peerj-07-7843-s022.jpg]

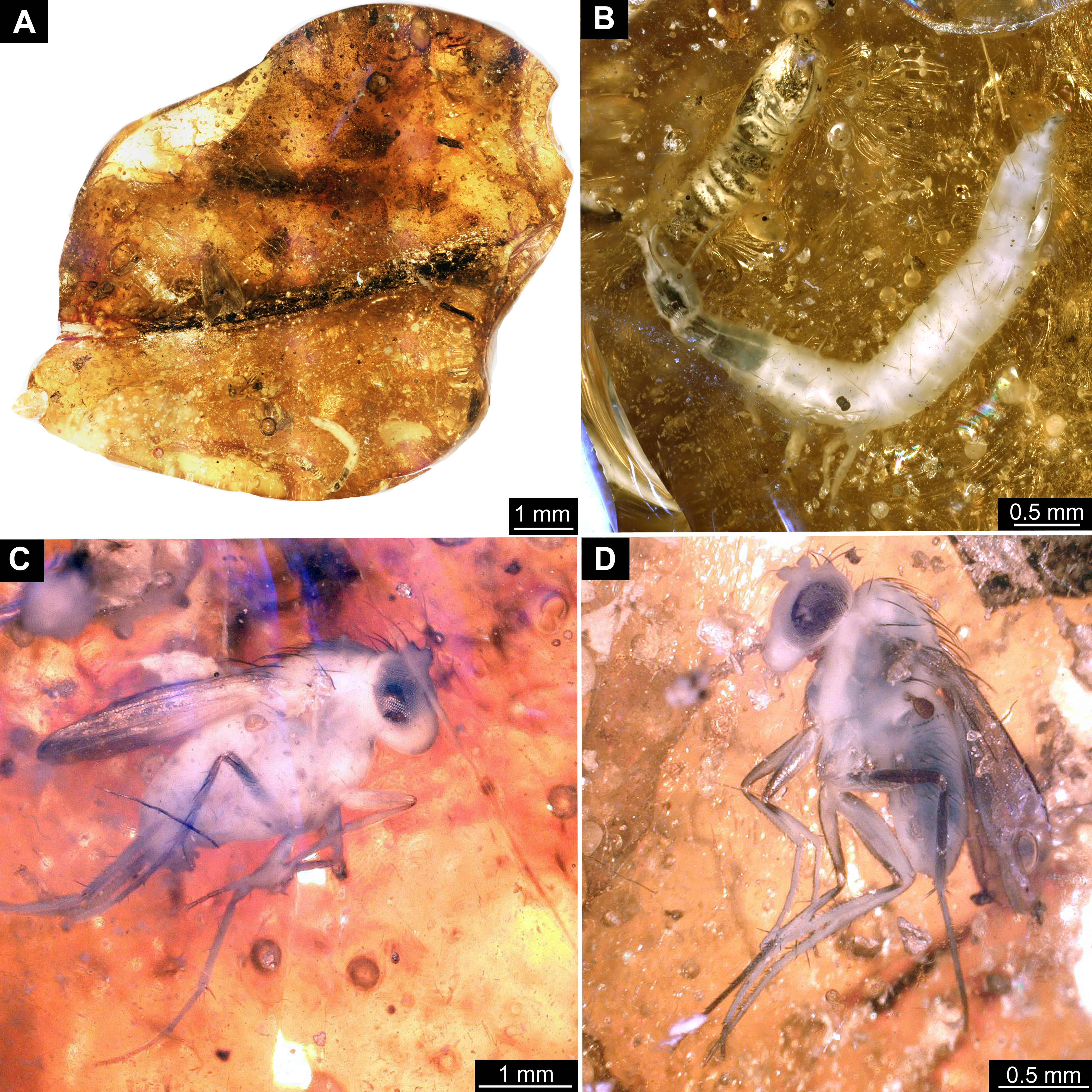

Supplement: Figure S23 — A) overview. (B) pupa (upper left) Mycetobia “morphotype 1”, (upper left) and larva of Neuroptera; lower right). (C, D) adult long-legged fly (Dolichopodidae). (C) specimen 1 (D) specimen 2. [file peerj-07-7843-s023.jpg]

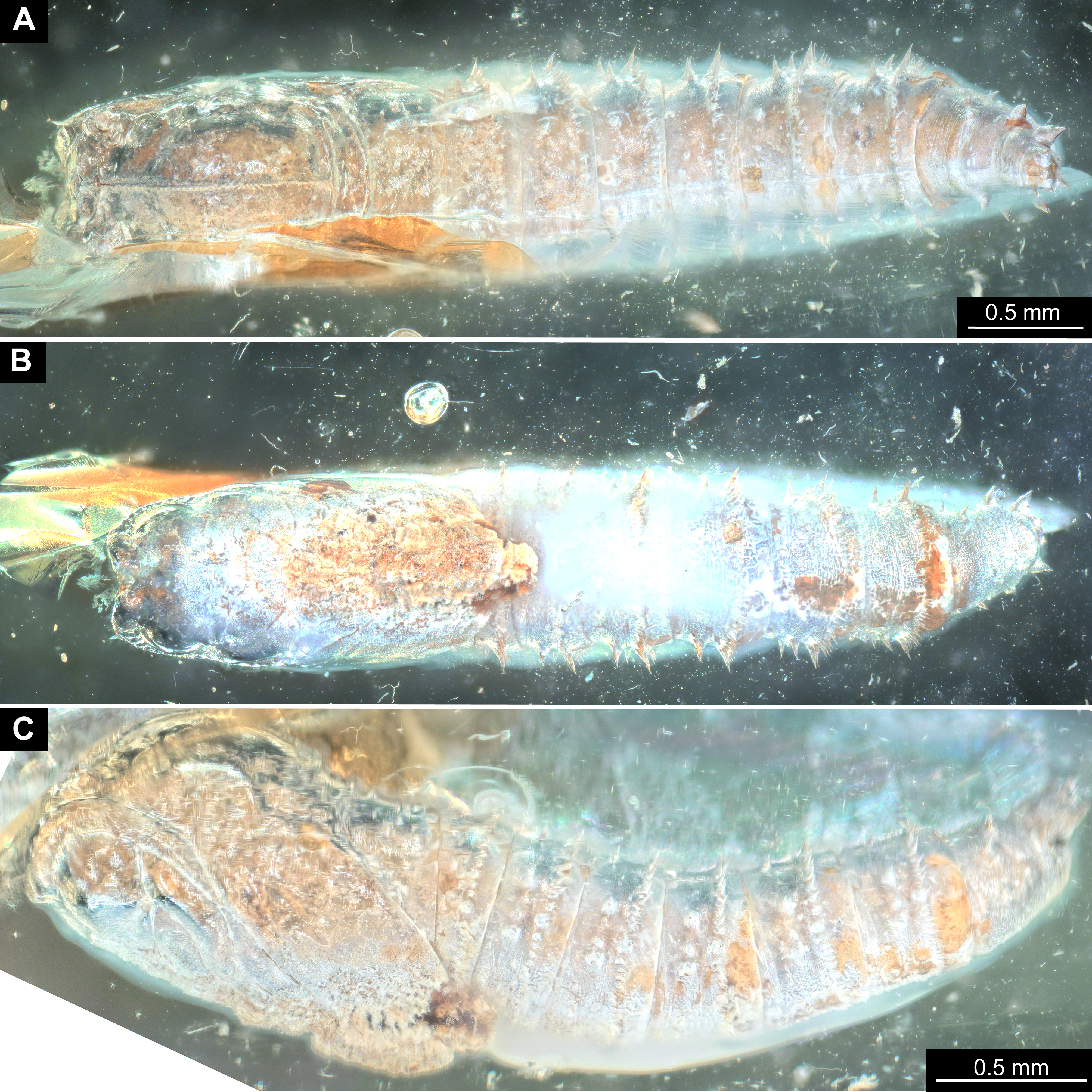

Supplement: Figure S24 — (A) habitus, dorsal view. (B) habitus, ventral view. (C) habitus, lateral view. [file peerj-07-7843-s024.jpg]

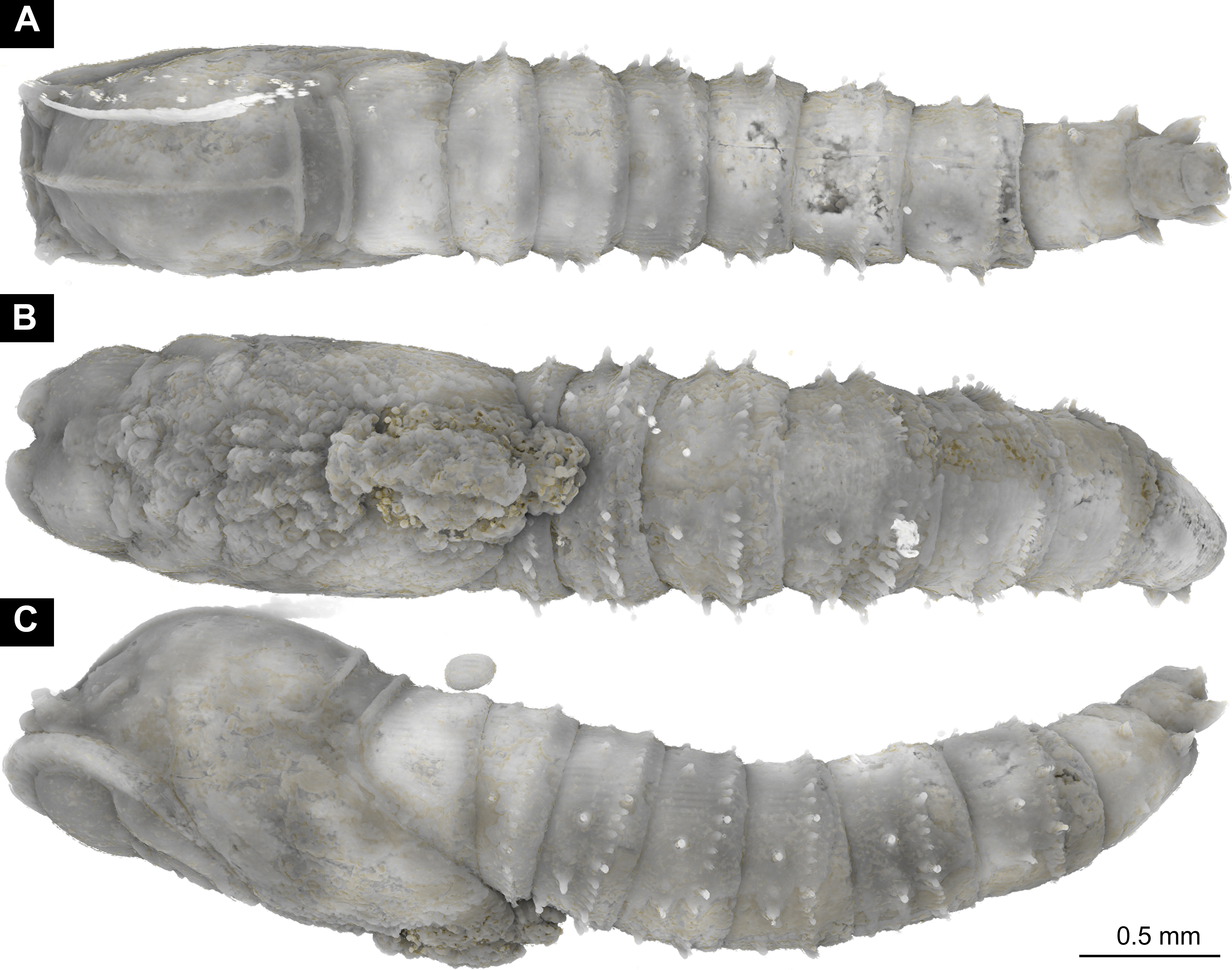

Supplement: Figure S25 — (A) habitus, dorsal view. (B) habitus, ventral view. (C) habitus, lateral view. MicroCT scanning credit: Marie Hörnig. [file peerj-07-7843-s025.jpg]

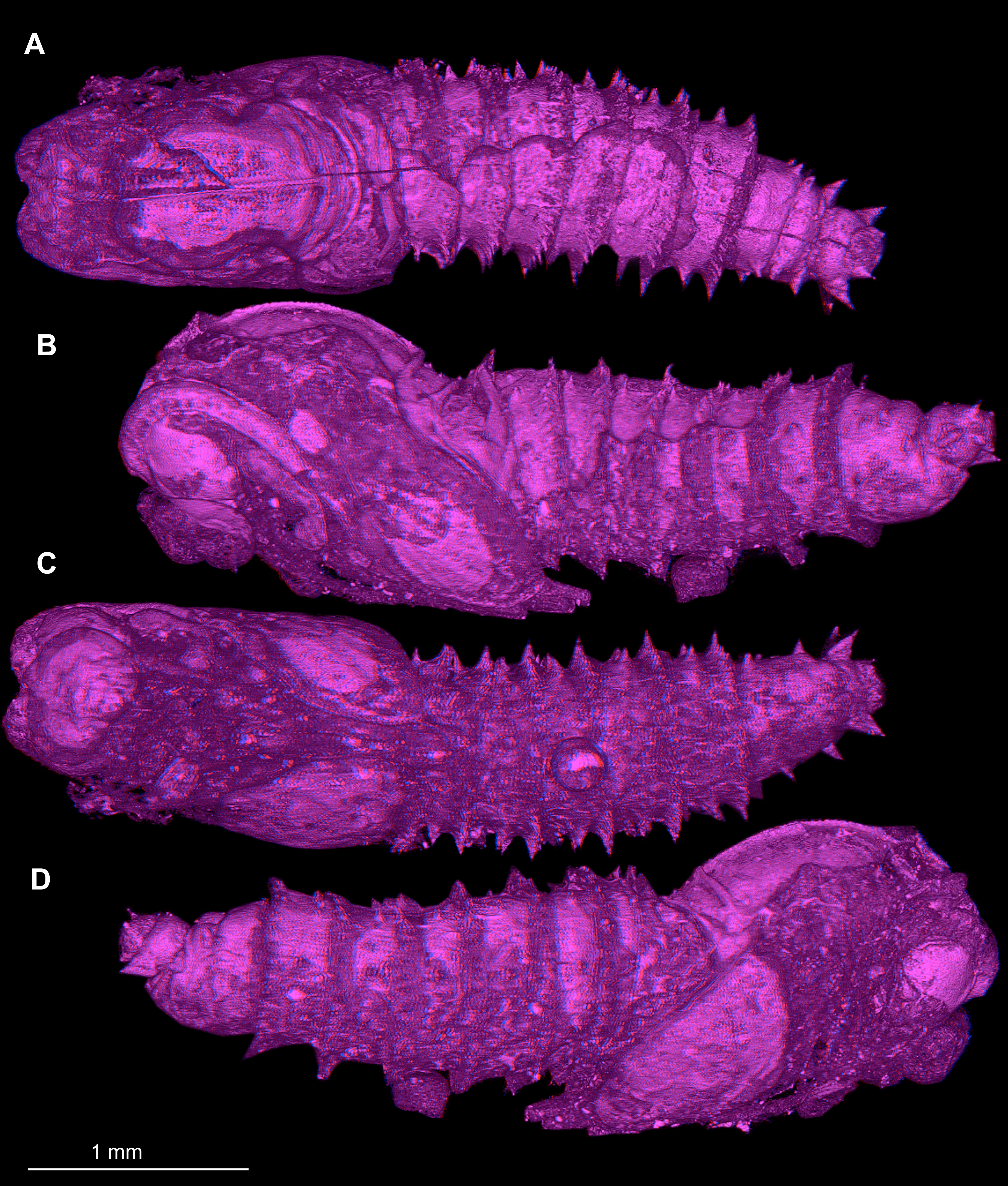

Supplement: Figure S26 — (A) habitus, dorsal view. (B) habitus, lateral view. (C) habitus, ventral view. (D) habitus, lateral view. All images red-blue stereo anaglyphs, please use red-cyan glasses to view. [file peerj-07-7843-s026.jpg]

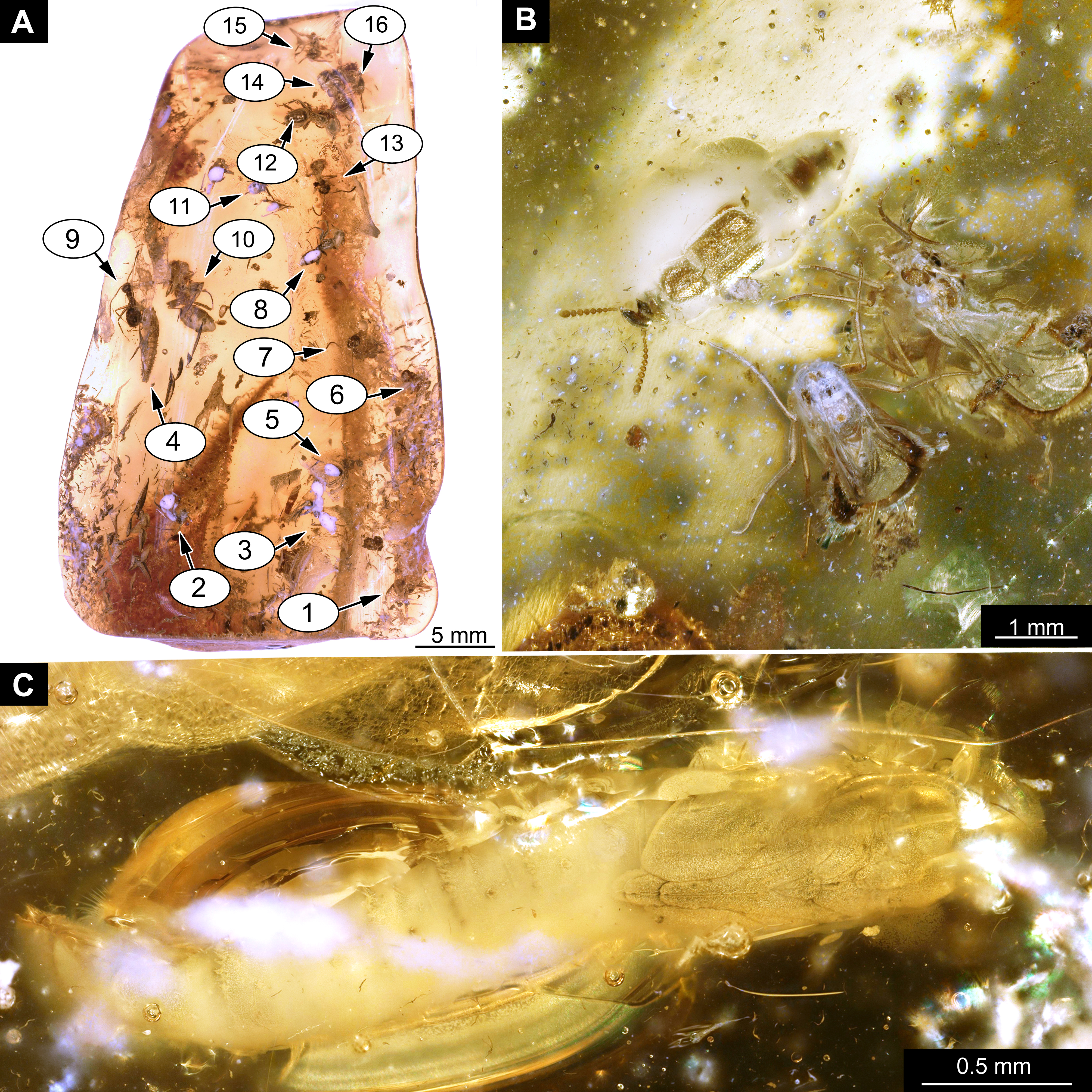

Supplement: Figure S27 — Fossil pupae, Mycetobia and syninclusions. (A)“morphotype 1” and syninclusions, GPIH, collection number AKBS-00071. 1, largely unidentifiable (Insecta); 2, 3, 5–9, 13, 15 ant worker (Lasius schiefferdeckeri Mayr, 1868); 4, Fossil pupa, Mycetobia “morphotype 1”; 10 ant worker (Ctenobethylus goepperti (Mayr, 1868)). (B) syninclusions to “morphotype 2”, PED, collection number PED-4866; adult rove beetle (Coleoptera: Staphylinidae), two adult gall midges (Diptera; Cecidomyiidae). (C) pupa of Mycetobia “morphotype 2”, GPIH, collection number L-7514, habitus, ventral view. [file peerj-07-7843-s027.jpg]

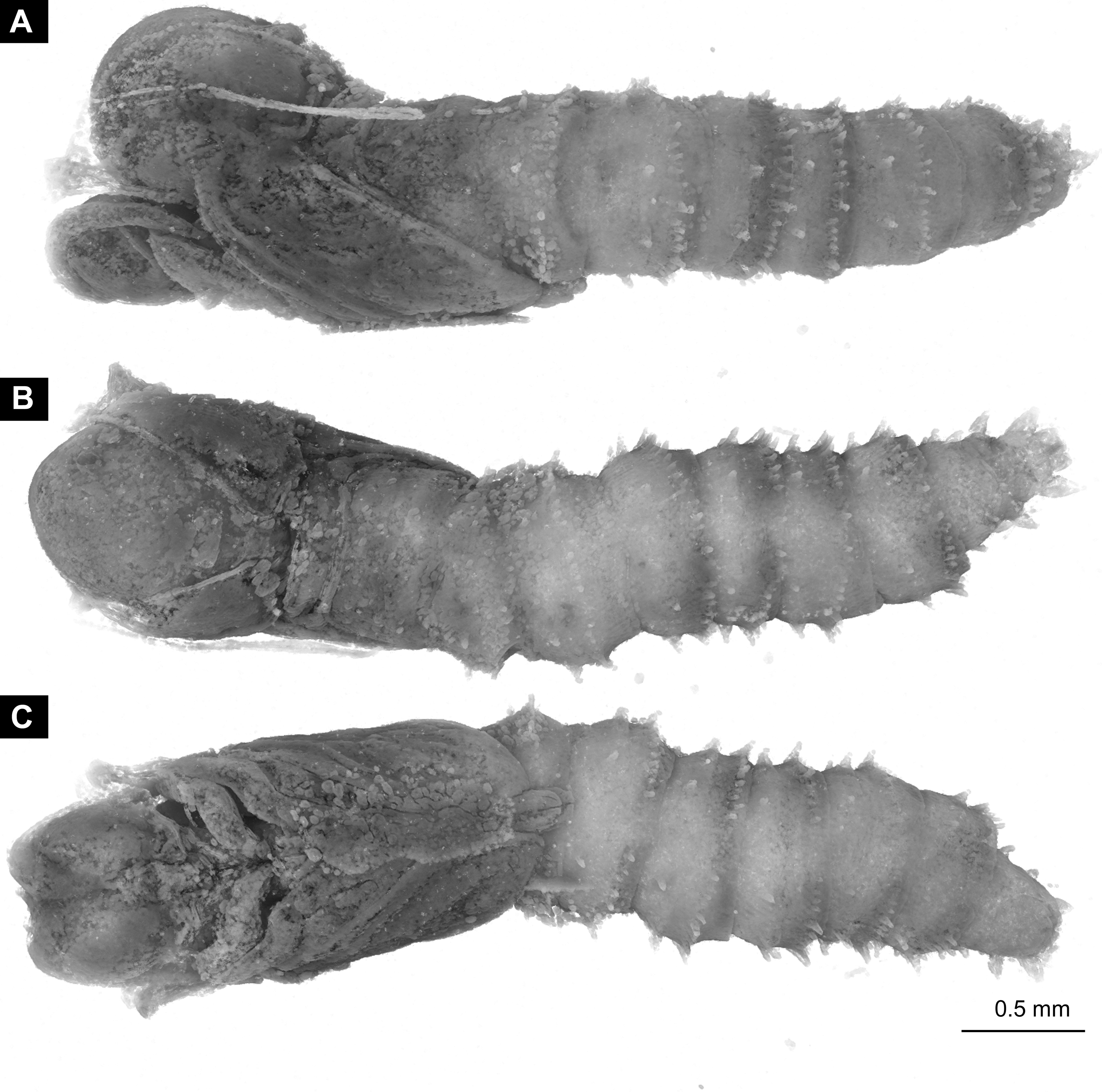

Supplement: Figure S28 — (A) habitus, lateral view, right body side, mirrored. (B) habitus, lateral view, left body side. (C) habitus, dorsal view. (D) habitus, ventral view. MicroCT scanning credit: Marie Hörnig. [file peerj-07-7843-s028.jpg]

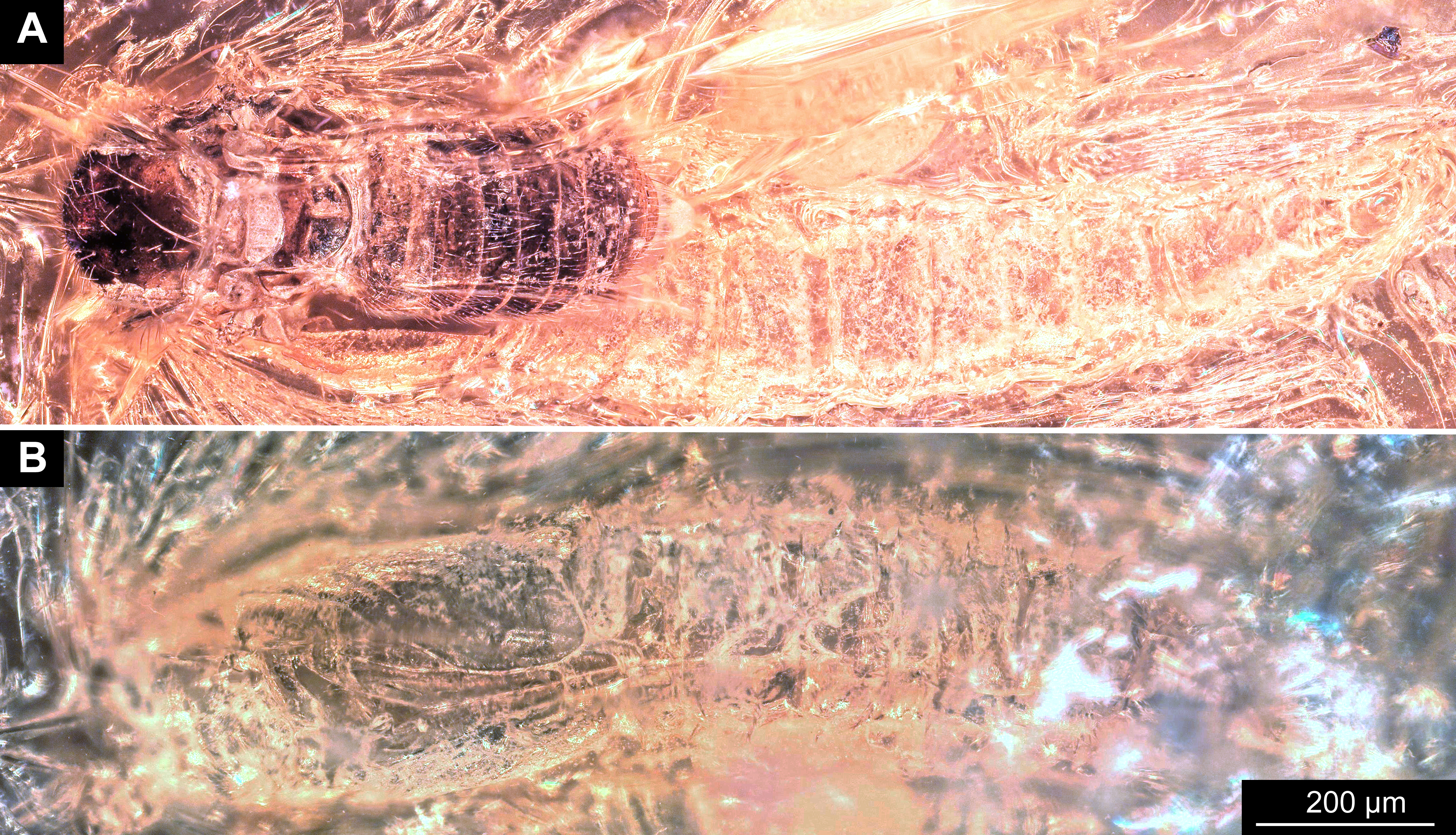

Supplement: Figure S29 — (A) habitus, dorsal view. (B) habitus, ventral view. [file peerj-07-7843-s029.jpg]
